# The Effect of Dietary Adaption on Cranial Morphological Integration in Capuchins (Order Primates, Genus *Cebus*)

**DOI:** 10.1371/journal.pone.0040398

**Published:** 2012-10-26

**Authors:** Jana Makedonska, Barth W. Wright, David S. Strait

**Affiliations:** 1 Department of Anthropology, University at Albany, Albany, New York, United States of America; 2 Department of Anatomy, Kansas City University of Medicine and Biosciences, Kansas City, Missouri, United States of America; Texas A&M University, United States of America

## Abstract

A fundamental challenge of morphology is to identify the underlying evolutionary and developmental mechanisms leading to correlated phenotypic characters. Patterns and magnitudes of morphological integration and their association with environmental variables are essential for understanding the evolution of complex phenotypes, yet the nature of the relevant selective pressures remains poorly understood. In this study, the adaptive significance of morphological integration was evaluated through the association between feeding mechanics, ingestive behavior and craniofacial variation. Five capuchin species were examined, *Cebus apella sensu stricto, Cebus libidinosus*, *Cebus nigritus*, *Cebus olivaceus* and *Cebus albifrons*. Twenty three-dimensional landmarks were chosen to sample facial regions experiencing high strains during feeding, characteristics affecting muscular mechanical advantage and basicranial regions. Integration structure and magnitude between and within the oral and zygomatic subunits, between and within blocks maximizing modularity and within the face, the basicranium and the cranium were examined using partial-least squares, eigenvalue variance, integration indices compared inter-specifically at a common level of sampled population variance and cluster analyses. Results are consistent with previous findings reporting a relative constancy of facial and cranial correlation patterns across mammals, while covariance magnitudes vary. Results further suggest that food material properties structure integration among functionally-linked facial elements and possibly integration between the face and the basicranium. Hard-object-feeding capuchins, especially *C.apella s.s.*, whose faces experience particularly high biomechanical loads are characterized by higher facial and cranial integration especially compared to *C.albifrons*, likely because morphotypes compromising feeding performance are selected against in species relying on obdurate fallback foods. This is the first study to report a link between food material properties and facial and cranial integration. Furthermore, results do not identify the consistent presence of cranial modules yielding support to suggestions that despite the distinct embryological imprints of its elements the cranium of placental mammals is not characterized by a modular architecture.

## Introduction

Morphological integration represents a widespread phenomenon, manifested in the coordinated change among phenotypic characters of an organism (e.g., [1]–[6]). In mechanistic terms, morphological integration can be defined as the covariation structure resulting from “the summed imprint of a succession of events, each of which leaves a distinctive covariation signal [produced by the differential] overlaying of variation introduced by developmental and environmental factors at different stages of development” [7] (p. 164). Numerous findings suggest that developmentally and functionally related traits share higher correlations than unrelated traits, and thus evolve in a coordinated fashion [8], because they are genetically linked, or because they are independent traits selected together [9], [10]. The fact that morphological characters involved in a common function tend to co-vary and thus co-evolve suggests that a proportion of this patterned variation might be a consequence of optimizing the adaptability of complex phenotypes. Functionally-linked characters are ultimately expected to evolve a common genetic basis, because genetic integration among these traits may avoid deleterious independent variation (which would disrupt functional systems), and so facilitate evolution by natural selection [11]. Cheverud [12]–[15], [5], Lande [16]–[18] and Wagner et al. [19] suggest that quantitative genetic theory is consistent with Olson and Miller’s [6] prediction that adaptively favorable coordinated units of evolution exist, and that the underlying pattern of genetic correlation depends on the patterns of stabilizing selection and new variation/covariation produced by mutation.

The most frequently cited causal factors of morphological integration among skeletal traits include heritable developmental effects including pleiotropic effects such as the influence of the transcripts of a single gene on the expression of multiple traits (e.g., structures whose development is driven by a common induction mechanism, such as the development of serially homologous traits) and genetic constraints (i.e., physical linkage among contiguous genetic loci). Architectural constraints might also induce covariation (e.g., the spatial modifications of the lateral basicranium likely have consequences for the spatial arrangements of facial structures [4], [20]–[24]). It is unknown if the structuring of integration is affected by gene-by-environment interactions or phenotypic plasticity, such as a shared muscular influence (e.g., the deltoid muscle attaches to the spine and acromion of the scapula as well as to the clavicle, and thus should affect all three bony elements).

Despite growing interest in integration, the environmental factors that induce functional integration in the skeletal system remain poorly understood. Integration in the primate skull has been extensively studied from a phylogenetic, ontogenetic, architectural, and genetic standpoint (e.g., [25]–[29], [8], [30], [31], [1], [32], [22], [23], [33], [7], [11]), yet this study is one of the few that test whether or not specific selective factors and associated functional demands are a significant source of craniofacial covariation.

### I. Hypotheses

This study is framed as a test of three sets of hypotheses. The first two hypotheses predict that dietary ecology has an impact on morphological integration in the primate cranium. They are named the *food material properties* hypotheses (H_(FMP)_) and the *heterochrony* hypotheses (H_(HET)_), and relate morphological integration to feeding biomechanics and development. A third hypothesis evaluates the evidence for the presence of a facial module in the cranium, and is named the *cranial modularity* hypothesis (H_(CMOD)_). The hypotheses’ predictions are:

**H_(FMP0)_:** *food material properties* null hypothesis; predicts no consistent difference in the patterns and/or intensities of morphological integration between species whose faces and crania experience notably different biomechanical loads associated with feeding.

**H_(FMP1)_:** first alternative; taxa that habitually or periodically rely on foods that are mechanically challenging to eat are expected to have more strongly morphologically integrated faces (with less unconstrained variation) than taxa that do not rely on such foods, either resulting from the action of natural selection, or as a consequence of non-heritable biomechanical remodeling that occurred during the lifespan of the individual.

**H_(FMP2)_:** second alternative; has the opposite expectation of H_(FMP1)_, namely, taxa that consume mechanically resistant foods exhibit less integrated faces as a result of differences in biomechanical remodeling.

**H_(HET0)_:** *heterochrony* null hypothesis; predicts no consistent difference in the patterns and/or intensities of morphological integration between conspecific males and females.

**H_(HET1)_:** first alternative; predicts that males in sexually dimorphic species will display higher levels of facial integration than conspecific females, because their longer growth period provides additional time to adapt to environmental stimuli.

**H_(HET2)_:** second alternative; has the opposite expectation as H_(HET1)_, namely that males have less integrated faces than females as a result of sex-specific growth differences affecting biomechanical remodeling (see explanation following hypotheses).

**H_(CMOD0)_:** *cranial modularity* null hypothesis; predicts the absence of an individualized facial and oral modules within the cranium.

**H_(CMOD1)_:** alternative; predicts the existence of an individualized facial module within the cranium, and an individualized oral module within the face, both being distinct from the cranial base and more integrated than the whole cranium.

#### 1. Diet and cranial variation

The selectively important foods described in H_(FMP1)_ that are mechanically challenging to eat may be fallback foods (resistant, less desirable foods consumed when preferred foods are not available; e.g., [34]), but they could also represent mechanically resistant preferred foods. The key requirement of H_(FMP1)_ is simply that the food items in question are difficult to process but nonetheless selectively important; in other words, the inability to efficiently consume the items would have a deleterious impact on fitness. In such cases, H_(FMP1)_ predicts that morphologies of the feeding apparatus that decrease feeding performance would be selected against, and a functionally advantageous pattern would spread in the population. The latter should result in an increase in integration intensity, because selection reduces variation. In theory, such adaptation could also be reflected in the number of elements recruited in the functional complex under selection. One could hypothesize that such differential recruitment of elements in feeding might be driven by the necessity to produce higher or more frequent bite forces, which results in the propagation of higher magnitude or more repetitive strains in more craniofacial regions.

H_(FMP1)_ predicts integration among traits related to two constraints – the ability to generate appropriately high or repetitive bite forces, necessitating facial morphologies associated with high muscular mechanical advantage, and the need for a craniofacial skeleton able to withstand the forces associated with feeding on resistant foods. These constraints should result in the evolution of craniofacial traits that efficiently absorb reaction forces and/or transmit loads to the food being bitten. As feeding loads either increase in magnitude or become more repetitive, there should be a greater need to evolve functionally advantageous morphological character complexes, and for decoupling feeding functions from other (e.g., sensory) functions performed by the same architectural complex. An analogy to the latter situation is suggested by Young and Hallgrímsson [35], who showed that serially homologous characters in the mammalian fore- and hind limbs are correlated, but in species like bats which possess highly derived forelimbs, such correlation is absent, presumably because selection for flight decoupled development of the forelimb from that of the hind limb.

The link between diet and craniofacial morphology is firmly established (e.g., [36]–[43]), and interspecific cranial differences indicate that the facial skeleton is subjected to strong selection (e.g., [44]). The expectation of H_(FMP1)_ rests on several lines of evidence. Numerous studies have found that the face, and particularly its oral subunit, contributes most extensively to cranial integration in primates (e.g., [27], [29], [30], [45]). Research carried out by Marroig and Cheverud [27] provides the strongest published evidence, to date, that diet might structure integration. In this study, each species was assigned percentages of different food types consumed year round such as fruits, insects and leaves. Then for a group of taxa, a dietary similarity matrix was built using a variable reflecting pair-wise similarity between taxon A and taxon B calculated using the formula:
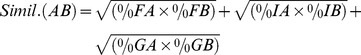
(1)where F stands for fruits, L stands for leaves and I stands for insects. Subsequently, the dietary similarity matrix was correlated with its corresponding morphological integration similarity matrix. However, such analyses do not specifically target the foods whose consumption might compromise the functional activity of the jaws, lead to injury and the inability to feed. Furthermore, such analyses dilute the potential effect of such resistant foods on morphological integration (e.g., by expecting that the proportion of insects consumed might induce similarity in morphological integration). Moreover, such analyses do not acknowledge Liem’s paradox and the evolutionary importance of fallback foods.

Goswami [46] reported a lack of strong and significant correlation between similarly built dietary similarity matrices and morphological integration matrices in marsupials, a result, which might be due to the lack of consumption of mechanically resistant foods by these species, or, as proposed, to the early ossification of facial structures directly involved in feeding, an adaptation stemming from the early birth of underdeveloped offspring combined with the necessity to suckle. Within placental mammals, diet quantified in a way similar to Marroig and Cheverud [27] was shown to be significantly or marginally significantly correlated with morphological integration only in Arctoidea (bears), when controlling for phylogeny whether or not the allometric component of variation is included and when phylogenetic and allometric signals are not excluded from the data, in Mustelidae (weasels) when allometric variation is preserved in the data whether or not phylogeny is accounted for, and in Musteloidea (raccoons, weasels and pandas), regardless of phylogeny and allometry [47]. Notably, these taxonomic groups differ from other Carnivora in that they contain many species that include an important fruit and/or leaf component in their diets. Importantly, at least some species of bears focus on resistant foods such as acorns, hickory nuts, beechnuts, hazelnuts and pine nuts during the autumn when tremendous weight gain is critical to ensure survival during the winter.

The factors responsible for the structuring of dietary integration as well as its underlying mechanism (i.e., heritable, selection-driven characteristic *versus* gene-by-environment interaction effect) remain unknown. Zelditch [25] found that the dietary and behavioral changes in placental mammals associated with the transition from suckling to mastication occur only after neonatal integration has been re-patterned into integration designed to withstand the effects of grinding. Thus, selection may act on a given ontogenetic stage and thereby lead to adult cranial integration in advance of a change in behavior. The elements of the facial skeleton in placental mammals follow a typically somatic postnatal growth pattern that extends into early adulthood [48], [11], which arguably favors the influence of gene-by-environment effects on facial anatomy and function. Skeletal growth can be influenced in many ways by many factors, and mechanical loads are among the best documented [49]. Indeed, numerous reports indicate that craniofacial morphology clearly responds to diet, in other words the physical characteristics of food can influence facial morphology during the lifespan of an individual [50]–[54]. There are no experiments suggesting that cranial integration in species adapted to a soft diet is affected by the adoption of mechanically challenging diet. Thus, it is unknown if traits that jointly experience high strains tend to covary. If this is the case, it would be unknown if a higher integration would result from the same regions experiencing *higher strain* or from *more regions* experiencing strains higher than a particular physiological threshold. An interesting study was carried out by Corruccini and Beecher [55]; [50], [53], who found that groups of placental mammals that had been fed artificially soft diets during development exhibited increased morphological variability and decreased dentognathic integration (including malocclusion) relative to groups that had been fed harder diets. This suggests that a normal loading environment might be essential for the functional integration of gnathic morphologies [56]; (see also [57] for results on limbs), and that functional integration relies at least in part on gene-by-environment interactions.

Vinyard [58] found no association between dietary type expressed as percentages of foods consumed and covariation structure of the mandible of galagos. Despite the varied diet of galagos, it was argued that the ability to obtain and digest gum might be the fundamental adaptation of this group [58]–[64]. If this is the case, the lack of association between diet and covariation structure might not be as surprising. A comparison of biomechanical performance of the skulls of tree-gouging primates indicates little evidence for increased force-production or load-resistance abilities, which led to the prediction that the skulls of tree gougers do not have to generate high biting forces [65].

The second alternative *FMP* hypothesis, H_(FMP2),_ expecting that the faces of species adapted to the consumption of mechanically-resistant foods will be less integrated, is based on a prediction deduced from research carried out by Wood and Lieberman [66]. The authors did not specifically address integration, but suggest that “skeletal structures whose development is strongly influenced by epigenetic stimuli, especially those subject to high and frequent magnitudes of masticatory strain will be more variable within species than those that are routinely subjected to lower strain magnitudes” [66], which conceivably might affect correlated variation in the facial skeleton. Extending this hypothesis from different modules to different species would imply that species whose faces typically experience low strains will have less variable morphology than species whose faces experience higher strains. The Wood/Lieberman hypothesis has engendered a lively debate (e.g., [67], [68], [56]), but its value lies in the fact that it allows one to evaluate the nature of interactions between ecological stimuli and integration from a phenotypic plasticity standpoint.

This review of the existing literature led us to think that broad-scale dietary classifications (e.g., fruits, leaves, insects, etc.) do not target a specific environmental factor that might account for changes in integration. Rather, the material properties of selectively important foods are likely to play a critical role in structuring the patterned variability of the cranium. Clearly, the potential association between food-processing behavior and facial integration requires more attention.

#### 2. Ontogeny and morphological integration

The second set of hypotheses tested here, the *heterochrony* hypotheses, link ecology to development. H_(HET1)_ predicts that males in sexually dimorphic species will display higher levels of facial integration than females. H_(HET2)_, following the Wood/Lieberman [66] prediction, has the opposite expectation. Both predictions imply that integration will be sexually dimorphic due to gene-by-environment interactions in the cranium, and follow from the observations that the face does not terminate its growth until early adulthood and that growth spurts are sex-dependent [69]. Specifically, the ossification pattern of the face is largely driven by soft tissue growth [70]–[72], including instances in which mechanical forces up regulate transcription factors in sutures to induce osteogenesis [7], [73]–[76].

The *heterochrony* hypotheses require that bone remodeling is responsive to biomechanical loading during development, and this premise appears to be well supported. Many studies have demonstrated increased cortical remodeling in skeletal regions experiencing elevated strains under both normal and experimental loading conditions [51], [52], [77]–[86]. Furthermore, evidence suggests that cortical bone is primarily responsive to strain prior to sexual maturity, both in terms of the rate of new bone growth and the rate of turnover [87]. Bouvier and Hylander [52] reported that the distribution of cortical bone remodeling in the macaque face coincided with peak mechanical strains during mastication in immature animals, while remodeling was observed in both high and low strain regions in the adults. A further expectation of H_(HET1)_ would be that integration increases during ontogeny and this argument appears to be supported [25], [30]. Thus, basic principles of bone biology and previous work on morphological integration do not contradict the *heterochrony* hypotheses *a priori*. Note that intraspecific sex-linked differences in craniofacial integration might potentially reflect the action of selection at later stages of development instead of gene-by-environment interaction. However, if such differences are consistently expressed among species characterized by differing dietary adaptations, then the likelihood of heritable sex-dependent differences decreases.

#### 3. Cranial modularity

The *cranial modularity* hypotheses will evaluate the evidence for a modularly organized cranium using both a new landmark protocol that is not reliant on suture intersections but is relevant to biomechanical performance.

The growth and development of the mammalian cranium is a complex process, involving different embryological tissue origins, modes of ossifications and hormonal influences [12]. Although defining integrated suites of characters in complex structures such as the skull is problematic because they tend to exhibit high levels of correlation not only within but also between regions [88], data collected thus far indicate that the skull has a hierarchical modular architecture, in which smaller, relatively independent subsets of highly correlated traits, or modules, are interlocked within larger, less integrated modules. For instance, the oro-facial complex is more tightly integrated than the skull as a whole (e.g., [26], [27], [45]). It has been suggested that the modular structure of the cranium roughly matches its developmental pattern. A large scale analysis of cranial modularity carried out by Goswami [45] in placental mammals identifies three highly integrated modular units, including two facial modules (anterior oro-nasal, molar) and the cranial base, and three weakly integrated modules, two facial modules (orbital, zygomatic-pterygoid) and the cranial vault. Goswami [45] further reports that theoretical correlation matrices testing specific hypotheses of modularity indicate that shared function, tissue origin, and mode of ossification are significantly correlated with observed correlation matrices for the mammalian cranium. Thus, the modular structure of the cranium roughly matches its developmental pattern. The bones of the face, the anterior cranial base, and the frontal bones of the cranial vault differentiate from neural crest-derived mesenchyme, while the posterior cranial base and posterior vault are derived from mesoderm [7], [89], [90]. The occipital region surrounding the foramen magnum differs from the rest of the skull in that it differentiates from the sclerotome of the occipital somites [4], [11], [90]–[92]. Based primarily on the type of ossification and maturation they undergo, the osseous elements forming the braincase can be grouped into cranial vault and cranial base. The basicranium and the bones surrounding the sensory capsules arise via endochondral ossification in which a cartilaginous phase exists before bone formation, while the cranial vault bones and the bones forming around the viscerocranial cartilaginous elements in the face differentiate directly into bone via intramembranous ossification, without a preexisting cartilaginous phase [7].

Despite the early differentiation of the face from the brain case and the bony orbits, growth factors and morphogens intervene in the generation of integration among those developmentally distinct units that ensures the structural integrity of the cranium [93]–[96], [11]. In fact, non-embryological hypotheses of modularity, such as models considering functional interactions coupled with an allometric component were reported to provide the best models for observed cranial phenotypic variation [25]. Furthermore, it was shown that patterns of artificial cranial deformation result in significant indirect effects on the face, the cranial base and mandible [97], [98]. In addition, several previous analyses have repeatedly suggested that the spatial modifications of the lateral basicranium have consequences for the spatial arrangements of facial structures [20]–[22]. The lateral basicranial elements, whose morphological development extends long after birth, are more strongly integrated with the mandible than are midline basicranial elements [22]. Furthermore, the depth of the middle cranial fossae appears to modulate facial length variation [22]. Specifically, an antero-lateral expansion of the middle cranial fossae rotates the face ventrally placing it under the anterior cranial fossa [23], which has been argued to lead to a shortening of the oropharynx and a decrease in projection of the upper face [4], [24].

In summary, during ontogeny integrating effects create inter-relationships among developmentally distinct cranial regions. This study benefits from a sample of closely related species characterized by differing dietary adaptations suitable for addressing questions of cranial and facial modularity in the crania of adult individuals.

### II. *Cebus*: a Model Organism for Examining Ecological Influences on Integration

To test the hypotheses of interest, the cranial architecture of five capuchin species was examined and compared. Capuchins, a radiation of successful south- and central- American platyrhine monkeys all belonging to the genus *Cebus*, are excellent models for studying dietary diversity, the evolutionary role of fallback foods, and their relationship to fine-scale dentognathic variation in closely related species.

The genus *Cebus* contains several primarily soft-fruit-eating taxa known as “non-tufted” or gracile capuchins, *C. olivaceus*, *C. albifrons* and *C. capuchinus*, and several “tufted” or “apelloid” taxa, including *C. apella s.s.*, *C. libidinosus*, and *C. nigritus*, classified as hard-object feeders by many authors (e.g., [99], [100]). Molecular and skeletal comparative analyses indicate that all apelloid capuchins form a clade (in fact until recently all of them were assigned to a single species), while cytogenetic and postcranial data indicate that the gracile *C. olivaceus* is more closely related to the hard-object feeding species than it is to the other gracile capuchins [101], [102], [42]. Thus, these data suggest that the gracile capuchins form a paraphyletic group. However, recent genetic [103] and morphological [104], [105] studies suggest that the robust and the gracile capuchins represent two distinct adaptive radiations (the former group originating in the Atlantic Forest, while the latter group originating in the Amazon) that diverged from a last common ancestor, who lived approximately 6.2 millions of years ago. The identification of two monophyletic capuchin clades led Lynch Alfaro and colleagues [103], [105] to propose a division of the members of the capuchin radiation into two genera, *Cebus* including the gracile capuchins and *Sapajus* including the robust capuchins. This manuscript follows the classification effective before the proposed rehabilitation of *Sapajus* made by Lynch Alfaro et al. [103].

Compared to other platyrhine species, both *C. apella* and *C. olivaceus* exhibit more anteriorly attached *masseter* and *temporalis* muscles, and both species are characterized by thick molar enamel [42]. In fact, *C. apella* was shown to have the thickest molar enamel of all non-human primates [100]. Compared to sample of primates consisting of *Ateles*, *Alouatta, Chiropotes*, *Pithecia* and *Saguinus*, *C. apella* and *C. olivaceus* were shown to exhibit the widest incisor rows, the largest postcanine occlusal areas, and relatively large canine areas (with *C. apella* exceeding all species) [37], [42]. Thus, it is reasonable to hypothesize that some of the adaptations of *C. apella* allowing the generation of high bite forces and the withstanding of high reaction forces were inherited from the last common ancestor of the *C. olivaceus* and the apelloids.

Within the capuchins, *C. apella* has a significantly greater mechanical advantage at the incisors and molars than *C. olivaceus*. Furthermore, *C. apella*’s jaws were found to better resist parasagittal bending, vertical bending at the symphysis, wishboning and lateral torsion [106]–[108], [42]. In addition, *C. apella* is characterized by larger incisor, canine and molar cross-sectional areas than *C. olivaceus*, which help dissipate bending stresses produced when scraping, biting and chewing objects [37], [42].

The differences in cranial and mandibular morphology between the tufted and untufted capuchins have been attributed to the processing of hard and tough foods by the tufted species [109]–[111], [42], [112]. Wright [42] comments: “The opening of mechanically challenging foods [in *C. apella*] with the anterior dentition requires the ability to powerfully scrape or bite with the incisors, to puncture with the canines, and to statically load a tissue between the teeth while it is torn by pulling back with the nuchal and back muscles, or by holding the head still and pulling away from the face with the hands and upper limb. The biomechanics of the capuchin craniodental complex appears to be particularly well-adapted for these functions” (p.490).

In addition to canine and incisor use in *Cebus apella* a number of studies suggest high levels of premolar loading [113]–[115], [42].

The apelloid species *C. apella s.s.*, *C. libidinosus* and *C. nigritus* differ in their food exploitation strategies. *C. apella s.s.* is the most adept at nut-cracking with the mesial teeth, and also occasionally processes tough foods with its molars. *C. libidinosus* also consumes hard palm nuts, but reduces the mechanical load on its face by using tools to initiate cracks in the shells [112]. Even in tool-using capuchins, the first task is to tear the tough, fibrous husk from the palm nut using the incisors and the canines. In contrast, *C. nigritus* is known to frequently cyclically grind the pith of palm leaves, leaf stems of legume plants and woody parts of plant species with the postcanine dentition during the dry season [116]. In some populations of *C. nigritus*, the primary food resource during periods of resource scarcity (up to 73.6%) is the leaf bases of bromeliads [117], [118]. Furthermore, *C. nigritus* populations have been reported to rely on tuberous roots of cassava plants during the dry season [119], as well as on the hearts or the apical meristem of palm trees during the winter season [120], highlighting once more dietary differences between apelloid species.

The gracile, or untufted species *C. olivaceus* is well studied ecologically [121], [122], [118] (see below). Less is known about the ecology of *C. albifrons,* but this species was shown to possess the most gracile phenotype of all capuchins in terms of several biomechanical parameters (i.e., temporal fossa area, relative mandibular ramus area, relative coronoid process area, relative symphyseal area, relative mandibular corpus area at M_2_ and M_3_) [123]. Furthermore, *C. albifrons* is the only gracile capuchin that possesses a significantly simpler sagittal suture pattern compared to *C. apella*. It was hypothesized that remodeling due to elevated strain energy linked to the consumption of mechanically challenging foods is responsible for the ontogenetic modeling of cranial sutures that change from linear at birth to inter-digitating at maturity [124], [123]. These findings suggest that the craniofacial skeleton of *C. albifrons* is poorly designed to, and does not withstand or generate high biting forces.

Consistent with these findings, tufted and untufted capuchins are known to turn to different foraging strategies during seasons of food scarcity. While *C. apella* individuals at many sites turn to a less preferred food still readily available in their core range [125] by “switch[ing] from generalist foragers during the wet season to palm nut specialists during the dry season” [126], [118], *C. olivaceus* and *C. albifrons* increase their day ranges and start travelling widely in search of food [127], [126], [118].

The plant tissues that *C. apella* processes with its incisors and canines have an average toughness of 1110.54 J/m^2^, while plant tissues processed by the incisors and canines of *C. olivaceus* are characterized by an average toughness of 1042.06 J/m^2^ [42]. Similarly, Wright [42] reports that foods processed with the premolars and molars by *C. apella* are characterized by an average toughness of 668.56 J/m^2^, while those processed by the premolars and molars of *C. olivaceus* have an average toughness of 390.04 J/m^2^. The differences in average values were statistically insignificant because of the larger toughness variance associated with the foods eaten by *C. apella*. However, the coefficient of variation of foods processed with the incisors and the canines by *C. apella* is nearly twice as large as this associated with the foods eaten by *C. olivaceus*. In *C. apella*, the maximum toughness values associated with food items opened with the incisors and canines are more than twice the maximum of the toughest fruit opened by *C. olivaceus*, while the maximum toughness values of the food items chewed by *C. apella* is nearly four times larger than the toughness associated with the foods chewed by *C. olivaceus* (*C. apella* was observed to masticate a palm leaf with a toughness of 10809.80 J/m^2^). The implication is that *C. apella* does not restrict its dietary choice to mechanically resistant foods. Rather, the ability to open and chew extremely tough foods allows for dietary niche broadening and thus confers a selective advantage in periods of resource scarcity. In fact, the tufted capuchin prefers weak foods when they are available, and its derived craniodental complex appears to be “an adaptation for the use of a few exceedingly tough items” [42] (p.489), in other words, an adaptation to fallback foods.

*C. libidinosus* individuals living in cerrado-caatinga dry ecotone forests ingest foods that are tougher in terms of median and maximal values (maximum toughness = 12413 J/m^2^) than those ingested by *C. apella s.s.* living in tropical rain forests, yet morphologically the former species is characterized by more gracile absolute mandibular dimensions, with symphyseal dimensions not significantly different from those of *C. olivaceus*. This incongruence between dietary toughness data and jaw morphology data might be the consequence of *C. libidinosus*' use of tools [112].

## Materials and Methods

### I. Data

Data were collected as three-dimensional (3D) landmarks on surface models generated with 3D desktop laser scanner model NextEngine (NextEngine Inc., Santa Monica, CA) from the crania of adult specimens of known sex belonging to capuchin species exhibiting interspecific differences in the exploitation of mechanically resistant foods. Specimens were considered adults if they exhibited the entire set of fully erupted permanent dentition and a fused or closed spheno-occipital suture. The examined species include *Cebus apella sensu stricto, Cebus libidinosus, C. nigritus, C. albifrons* and *C. olivaceus* housed at the American Museum of Natural History, National Museum of Natural History and the Chicago Field Museum (Table 1).

**Table 1:** Sample size and composition.

Species/Subspecies/Sex	Sample size
***Cebus albifrons*** ** total**	**100**
*C. albifrons* males	49
*C. albifrons* females	51
*C. albifrons* ssp.	9
*C. albifrons aequatorialis*	6
*C. albifrons albifrons*	23
*C. albifrons cuscinus*	5
*C. albifrons versicolor*	35
*C. albifrons unicolor*	16
*C. albifrons trinitatis*	6
***Cebus apella s.s.*** ** total**	**97**
*C. apella s.s.* males	49
*C. apella s.s.* females	48
*C. apella* ssp.	11
*C. apella apella*	51
*C. apella fatuellus*	10
*C. apella macrocephalus*	25
***C. libidinosus*** ** total**	**78**
*C. libidinosus* males	39
*C. libidinosus* females	39
*C. libidinosus libidinosus*	41
*C. libidinosus paraguayanus*	37
***C. nigritus*** ** total**	**85**
*C. nigritus* males	40
*C. nigritus* females	45
*C. nigritus nigritus*	41
*C. nigritus robustus*	23
*C. nigritus x libidinosus*	21
***C. olivaceus*** ** total**	**77**
*C. olivaceus* males	48
*C. olivaceus* females	29
*C. olivaceus apiculatus*	39
*C. olivaceus brunneus*	8
*C. olivaceus castaneus*	20
*C. olivaceus nigrivittatus*	10
**Total sample analyzed**	**437**

Many specimens lacked subspecific designations. In such cases, geographic distribution maps from Fragaszy et al. [118] were used to infer subspecies.

### II. Data Acquisition

The NextEngine scanner, calibrated at the appropriate resolution, produces high quality surface scans with clearly detectable detail. Each generated virtual surface represents a “mesh”, composed of hundreds of thousands of triangles drawn between hundreds of thousands of 3D coordinates. A built-in digital camera captures images of the object, which are mapped onto the mesh, resulting in a texture rendering of the model [128]. Finished models were created using the ScanStudio HD software by placing virtual beads on the same spot in two different scan views.

The scanner mode was set to a resolution of 10, 000 points per square inch (associated accuracy = 0.005 inches), while surface texture was recorded as either grayscale or RBG color information with a resolution of 150 dots per inch. Each cranium was positioned on an incremented rotating platform connected to the scanner and held in place by an adjustable, rubber-padded part gripper. The chosen scanning protocol included one 360° scan (complete revolution of the rotating platform) with eight 45° divisions carried out while the cranium’s occiput is resting on the platform, stabilized in this position by the part gripper. The remaining surfaces were captured through manual repositioning of the cranium by: (a) a bracket scan (scans from three consecutive angles) at 14 divisions capturing the anterior view of the face, and its antero-lateral perspectives when the cranium rests on its inferior surface, (b) a single scan of the occipital area, and (c) additional single scans as needed. Estimations of intra-observer precision in digitizer-based and 3D model-based coordinate measurements indicate that the former is associated, on average, with a smaller error (for human crania standard deviations of measurements are 0.79 and 1.05 mm, respectively) [129]. A Microscribe digitizer yields the most precise coordinate data for sutural landmarks, while the digitizing of NextEngine scanner-derived models yields the most precise coordinate data for geometric landmarks. Very marked sutures were used as landmarks in this analysis (i.e., zygomatico-maxillary suture, spheno-occipital suture), thus the NextEngine scanner is an appropriate measurement tool. Once the virtual models were generated, they were exported for virtual digitizing in the Landmark Editor software [130]. Data on the virtual models were collected in the form of 3D coordinates of osteometric landmarks, which can be reliably identified. Prior to analyses, all samples were inspected for outliers due to measurement error in MorphoJ [131].

### III. Geometric Morphometrics and Statistical Approaches

The sampled landmark configurations were analyzed using geometric morphometric procedures. This approach preserves the detailed geometry of objects far better than traditional measurements, allows for precise localization of shape change, for a better quantification of anatomical features difficult to measure conventionally [132], and for an advanced statistical shape analysis.

**Figure 1:**
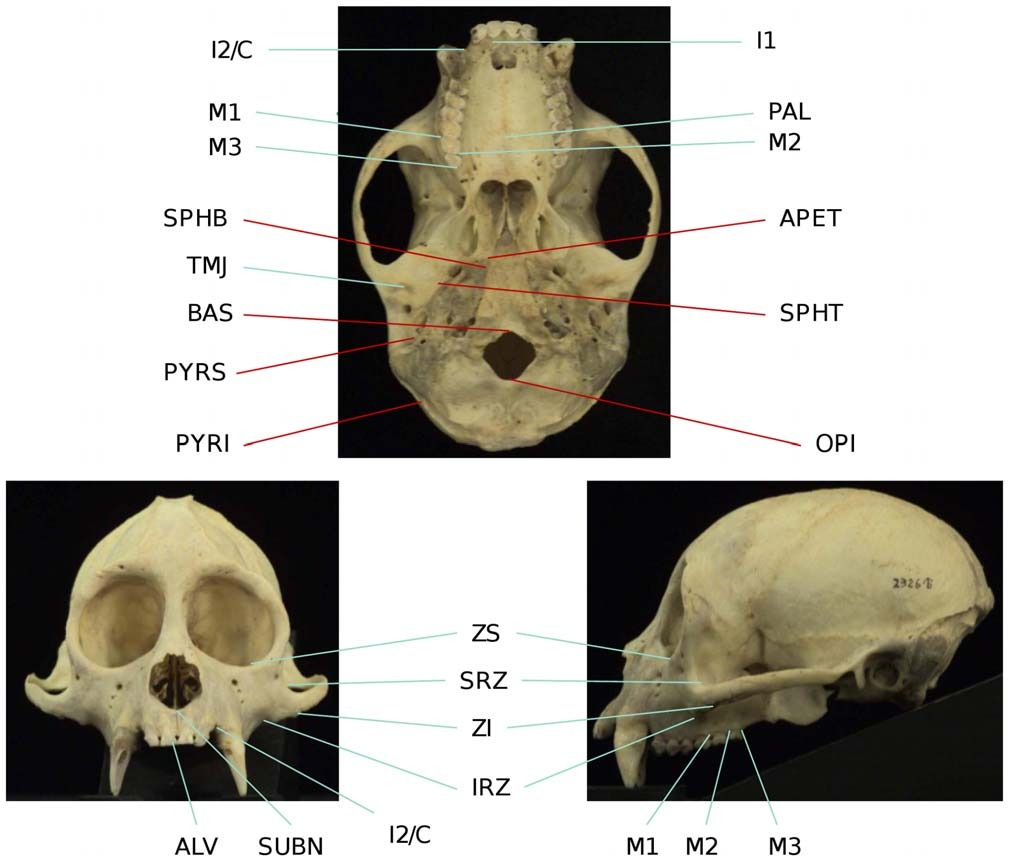
Landmarks used in this study. Facial landmarks in right green; basicranial landmarks in red. Photographs of the cranium of a *Cebus apella* individual obtained from the Mammalian Crania Photographic Archive of the Department of Anatomy, Dokkyo Medical University.

Integration between and within several purported facial and cranial modules (e.g., oral - zygomatic integration, facial and cranial integration, integration between pairs of units producing a maximized between-block modularity) was assessed between capuchin species and sexes. Twenty 3D landmarks (Table 2, Figure 1) were chosen to sample facial regions known to experience high strains during functional activity in anthropoids [133]–[135], or which represent anatomical correlates of muscular mechanical advantage [136], [137], as well as regions belonging to the lateral and midline basicranium. Landmarks positioned at homologous locations, or type I landmarks, and other landmarks easily identifiable based on various geometrical features were preferred.

**Table 2:** List of landmarks used in this study.

*Abbreviation*	*Definition*	*Module*	*Sub-module*	*Justification of choice*	*Type of landmark*
IRZ	Inferior root of zygomatic bone in lateral view	Face	Zygomatic	Origin of masseter muscle. Provides a measure of the height of arch. Area of maximum strain during molar, premolar and postcanine teeth biting (Richmond et al. [160]) in Macaca fascicularis and Au. africanus (Strait et al. [135]).	Type II
SRZ	Superior root of zygomatic bone in lateral view	Face	Zygomatic	Origin of masseter muscle. Provides a measure of the height of arch. Area of maximum strain, during molar, premolar and postcanine teeth biting (Richmond et al. [160] in Macaca fascicularis and Au.africanus (Strait et al. [135]).	Type II
ZI	Zygomaxillare inferior	Face	Zygomatic	Easily identifiable. Zone of maximum strain during molar, premolar and postcanine dentition biting (Strait et al. [135]).	Type I
ZS	Zygomaxillare superior	Face	Zygomatic	Easily identifiable. Zone of maximum strain during molar, premolar and postcanine dentition biting (Strait et al. [135]).	Type I
TMJ	The center of the temporo-mandibular joint	Face	Zygomatic	One of the parameters determining the maximum bite force. TMJ is a load-bearing joint subject to high strains (Hylander [161], Spencer [39]).	Type II
ALV	Alveolare	Face	Oral	Area of high strain during incisor biting.	Type I
I1	The alveolar margin of the first incisor at its buccal surface	Face	Oral	Measures the antero-posterior position of the first incisors, and incisor cross-sectional area.	Type I
SUBN	Nasospinale: meeting of the two premaxillary bones in the midline at the inferior-most margin of the nasal aperture	Face	Oral	Easily identifiable developmental homology. Zone of moderate strain during postcanine dentition biting in Macaca fascicularis (Richmond et al. [160]) and Au. africanus (Strait et al. [135]).	Type I
I2/C	Premaxillary suture at the inferior margin of the nasal aperture in the midline	Face	Oral	Easily identifiable developmental homology. Zone of moderate strain during postcanine dentition biting (Strait et al. [135]).	Type I
PAL	Mid-palatal point at the level of M1	Face	Oral	Provides a measure of palatal depth.	Type II
M1	P4/M1 contact at lingual alveolar surface	Face	Oral	Easily identifiable developmental homology. Indicates the position of a bite point.	Type I
M2	M1/M2 contact at lingual alveolar surface	Face	Oral	Easily identifiable developmental homology. Indicates the position of a bite point.	Type I
M3	Landmark immediately posterior of the M3	Face	Oral	Easily identifiable developmental homology. Indicates the position of a bite point.	Type I
SPHB	Sphenobasion, the meeting of the spheno-occipical suture with the petrous temporal bone	Neurocranium	Basicranium	Easily identifiable developmental homology.	Type I
APET	The apex of the petrous pyramid of the temporal bone	Neurocranium	Basicranium	Easily identifiable developmental homology.	Type I
SPHT	The meeting of the sphenotemporal suture with the petrous pyramid	Neurocranium	Basicranium	Easily identifiable developmental homology.	Type I
PYRS	The contact between the petrous pyramid and the posterior-most aspect of the external auditory meatus	Neurocranium	Basicranium	Easily identifiable developmental homology.	Type I
PYRI	Temporo-parietal-occipital junction	Neurocranium	Basicranium	Easily identifiable developmental homology.	Type I
BAS	Basion, the anterior-most point at the foramen magnum	Neurocranium	Basicranium	Easily identifiable developmental homology.	Type I
OPI	Opisthion, the posterior-most point at the foramen magnum	Neurocranium	Basicranium	Easily identifiable developmental homology.	Type I

In addition to biologically meaningful information, the 3D landmark coordinates encode information about the positioning of the specimens relative to the x, y, and z axes during data collection. To surmount the problem of inter-individual coordinate comparability, the 3D landmark configurations of the specimens were corrected for size differences (i.e., scaled to unit centroid size) and aligned via Generalized Procrustes Analysis (GPA), or superimposition [138]–[140].

Generalized Procrustes superimposition employs an iterative least-squares fitting technique, in which the shape configurations of all specimens from the sample are fitted to a randomly-chosen reference, followed by the computation of a sample mean as the arithmetic average location of all landmarks. In each iteration, the parameters for location and orientation that minimize the sum of squared distances between corresponding landmark coordinates on two configurations are estimated [141], [142]. The procedure is terminated when the change in sum-of-squares differences between configurations from one iteration to the next is negligible [141]. Procrustes-aligned coordinates lie in a hyper-hemispheric shape space known as Kendall’s shape space, which for samples with little variation (such as those containing similarly shaped, or closely-related taxa) is demonstrated to be a reasonable approximation of its orthogonal tangent plane projection [136]. This study uses full Procrustes superimposition in which centroid size of one is allowed to vary to minimize distance between shapes [142]. Klingenberg (MorphoJ users’ guide) notes that the full Procrustes analysis is robust against the influence of outliers. The software used to carry out most of the analyses described here, MorphoJ [131], projects the configurations to the linear space tangent of the Kendall’s shape space.

A correction for cranial asymmetry was performed prior to the Procrustes superimpositions.

It was suggested that the topic of symmetry and asymmetry is relevant to shape analyses even if these organismal properties are not the primary focus of a study, because the symmetry of morphological structures can pose statistical problems if it is not taken into account [143], [144]. For this reason, a mirror image of each configuration was generated, and the Procrustes analysis superimposed simultaneously the combined original and mirrored configurations [144]. Subsequent analyses were performed on the symmetric component of shape variation representing the variation, among individuals, in the average of their actual and reflected configurations [143], [145], [144]. Separate GPAs were performed on each species or sex.

#### 1. Assessment of measurement error

Intra-observer measurement error was assessed using the method developed by Yezerinac et al. [146] implemented in R [147]. Digitizing error was quantified by the repeated measures of a random subsample of 6 *Cebus albifrons* individuals, including males and females, several months after data collection. The sample of repeated measures was aligned via Procrustes superimposition and symmetrized in MorphoJ (symmetrization averages the left and right observations for bilateral characters). Measurement error was calculated as the percentage of total variance within a sample attributable to within-individual variation in landmark coordinates. An ANOVA was used to partition the variance of each landmark coordinate into within- and between-individual components, and the percentage measurement error was calculated as the ratio between the within-individual variance and the total variance multiplied by one hundred:
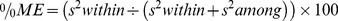
(2)


The measurement error yielded by the comparison protocol including all cranial coordinates was 2.17%, while this associated with the protocol including only facial coordinates was 3.7%; a measurement error under 5% is widely judged as acceptable. Furthermore, the actual measurement error affecting the entire sample of each species (between 77 and 100 individuals in this study) is certainly smaller, as Yezerinac et al. [146] point out that when sample size increases, the proportion of total variance due to imprecision associated with data collection decreases.

#### 2. PLS: the study of covariation between modules

Partial Least-Squares analyses (PLS) were performed to test if pairs of taxa and sexes show similar patterns and degrees of oral-zygomatic, facial-basicranium and maximum modularity (not limited to spatially contiguous subsets of landmarks) correlation. Furthermore, PLS allows assessing whether correlation between facial blocks is stronger than correlation between facial and basicranial measurements, which addresses the question of cranial modularity.

PLS aims to determine the correlated pairs of linear combinations of variables within one of the *a priori* chosen blocks that express the greatest proportion of covariance between blocks [140], [142], [148], [1]. For each sample PLS was carried out in MorphoJ on 3D coordinates which contain an allometric component and on residuals, in which the effect of size on shape was removed, by pooled within-group regression (for subspecies and sex) of the 3D shape coordinates on centroid size (i.e., the square root of the sum of the squared distances of the set of landmarks from their centroid). In many cases, allometry is the most important source of covariation among morphological characters; therefore, simply discarding allometric shape change leads to the analysis of non-realistic integration patterns and phenotypes, that contribute to a small percentage of covariation and that are likely not the principal target of selection. Thus, PLS was used to compare allometric and non-allometric covariation axes, in terms of both integration magnitudes and integration patterns.

It has been noted that sources of variation that induce unequal levels of population heterogeneity and population sub-structuring should be held constant in integration studies [149]. To ensure a controlled comparison of integration magnitudes across samples being compared, which contain different numbers of differently related subspecies, the data were transformed prior to all analyses into pooled within-groups correlation or variance-covariance matrices. Thus, subsequent analyses focus on the covariation between the 3D coordinate residuals after removing sub-specific differences in means. In the case of the PLS analysis carried out on residuals from regression on centroid size, the matrices were also pooled within-sex.

PLS was carried out within a single configuration rather than on shapes of parts considered separately as spatially distinct blocks (which is the most frequent use of PLS), because it is more appropriate to study covariation between the purported modules of the cranium in the context of the structure as a whole [150]. Choosing PLS within configuration (e.g., [151], [152], [28]) for cranial analyses rather than the more commonly used two-block PLS is justified, because the orientation of the chosen blocks relative to each other has important architectural and arguably even functional consequences.

The permutation tests offered in the PLS procedure concern the null hypothesis of complete independence between the two blocks. The p-values of all variables used to infer integration magnitudes were obtained through 10,000 randomization rounds. The RV coefficient [153]; [150] was used as a measure of overall covariation between the two landmarks partitions. It represents a multivariate analogue of the squared correlation coefficient between two sets of variables. The RV coefficient is calculated as the sum of the squared covariances between the variables in the two blocks divided by the squared variances and covariances within the two blocks [150].

Maximum cranial modularity two-block landmark partitions, or partitions yielding the lowest between-block covariances expressed as RV coefficients were obtained through the modularity function in MorphoJ. Subsequently, these partitions were used in PLS analyses.

Shape change along axes of covariation (or pairs of singular axes for each block) allowed a visual assessment of whether or not the vectors at landmarks are indicative of similar correlation trends between the groups of interest. In all figures containing shape changes illustrated by vectors at landmarks, shape change was magnified three times, to facilitate the reading of the graphs.

In addition to PLS analyses, Principal Components Analyses (PCA) were carried out to compare patterns and magnitudes of shape change at vectors at landmarks.

#### 3. Assessment of modularity with cluster analysis

Five cluster analyses (one for each species) using Ward’s method of linkage were used to determine whether conserved cranial modules exist. This approach is an alternative to the maximum modularity method developed by Klingenberg [131].

Hierarchical clustering using Ward’s method of linkage was carried out on individual species distance matrices derived from corresponding correlation matrices obtained from pooled within subspecies and sex residuals from regression on centroid size. The variables subjected to hierarchical clustering were landmark coordinates. The p-values at each of the dendrogram’s nodes were estimated using a bootstrap procedure with 1000 permutation rounds using the “pvclust” package in R. Two types of p-values were computed: approximately unbiased (AU) p-values, which are obtained via multiscale resampling and Bootstrap Probability (BP) p-values, computed using normal bootstrap resampling. In this work, discussion will focus on the AU p-values. When a bootstrap value is larger than 95 the hypothesis that a particular cluster does not exist can be rejected with an alpha level of 0.05.

#### 4. Comparison of integration indices calculated as the variance in eigenvalues of a correlation matrix. Assessment of modularity

The variance in eigenvalues [154] was used as an alternative metric of overall facial and cranial integration magnitudes, as a quantification of integration within smaller purported modules (i.e., oral, zygomatic) and within modules obtained through a maximum modularity test in MorphoJ. Values of eigenvalue variances of purported cranial modules were compared within species, to determine whether a hierarchical organization of the cranium exists (test of the *cranial modularity* hypothesis), and between species and sexes, to test the *FMP* and the *HET* hypotheses. Eigenvalue variances were computed on configurations excluding redundant bilateral landmarks for the following modules: oral (K (number of landmarks) = 8), zygomatic (K = 5), rostral (K = 8), molar (K = 5), basicranial (K = 8), facial (K = 13), cranial (K = 20) and maximum cranial modularity blocks (number of landmarks differs depending on the species, see results on maximum modularity). The specific prediction of the *cranial modularity* hypothesis is that since in a modularly organized structure integration within modules is higher than integration between modules, the integration magnitudes of at least some of the purported facial modules (e.g., molar, oral, zygomatic, rostral), will be higher than integration magnitudes within the entire face, while the face and the basicranium themselves are expected to individually be characterized by higher magnitudes than the cranium as a whole, which includes both of them.

The analysis aiming at comparing integration magnitudes among species calculated the eigenvalue variance (EV) from a pooled-within sex correlation matrix obtained from the Procrustes coordinates residuals from a regression on centroid size while controlling for sex and subspecies. The analysis comparing differences in integration between the sexes calculated the EV from a correlation matrix from the residuals from a regression on centroid size while controlling for subspecies. In structures characterized by weak correlations between variables, the variance in the dataset will be distributed across many orthogonal axes of covariation each explaining a limited proportion of overall variance, resulting in a low EV. Conversely, in highly integrated structures numerous traits will covary strongly, thus the majority of the variance will be summarized by a small number of orthogonal axes accounting for most of the total covariation, resulting in a high EV [154], [155].

A separate Procrustes superimposition, regression on centroid size, and correlation matrix was obtained for each module, species or sex. For each EV, a 95% confidence interval (95% CI) was obtained by resampling with replacement of the dataset for 1000 iterations, and a correlation matrix was obtained for each replicate. If the correlation between the actual correlation matrix and the correlation matrix of a replicate was greater than 0.95 (and greater than 0.90 for the cranial configuration analysis), this replicate was used to compute an integration index. All computations were performed in R.

#### 5. Comparison of integration indices at a common level of sampled population variance

Finally, an alternative approach founded on the rationale behind the use of eigenvalue variances was implemented to quantify and compare integration magnitudes across species and sexes at a common level of sampled population variance. The method developed by Young et al. [156] was implemented. Young et al. [156] found that if two traits are highly correlated, then increasing the sampled variance will improve their estimated correlation (resulting in a positive association between measures of overall covariation among measurements and the average trait coefficient of variation (CV), whereas if the correlation is low, then increasing the sampled variance will not improve correlation estimates. The implication of Young et al.’s [156] finding for the comparative analysis of integration is that when the covariance structures of the samples being compared are broadly similar, this relationship can be taken into account in order to make appropriate comparisons among integration magnitudes at a common level of sampled population variance. On the other hand, if no relationship exists between correlation and average trait variance (measured in this study by the average trait coefficient of variation), then it is reasonable to infer that the examined variables (inter-landmark distances from the right half of the cranium in this study) are not strongly and thus consistently correlated.

To correct for variance artifacts in integration, each species was resampled with replacement 1000 times and for each replicate a pooled within-sex correlation matrix was obtained. If the correlation between the actual correlation matrix and the correlation matrix of a replicate is greater than 0.95, this replicate was used to compute an integration index and an average trait CV. The generated sample of replicates was used to plot the relationship among the values of the two computed parameters, and trace its 95% confidence ellipses. Thus, the 95% confidence intervals of the integration indices can be compared inter-specifically in view of their associated 95% intervals of the average trait CV. The coefficient of variation of the eigenvalues (ICV) [157], [158], [149] calculated as the square root of the variance in eigenvalues divided by the mean eigenvalue was the preferred integration index.

ICVs were calculated for the cranial, the facial, the basicranial, the oral, the zygomatic, the rostral and the molar blocks. For each block, data used for the ICV computation included all inter-landmark distances from the right side of the cranium obtained from the 3D coordinates transformed into residuals from pooled within-groups regression on centroid size (regression pooled within subspecies and sex to test the *FMP* hypotheses, and within subspecies to test of the *HET* hypotheses).

ICV and EV are only partially redundant measures of integration. ICV differs from EV in that 1) the use of inter-landmark distances allows comparing integration magnitudes at a particular average trait CV level, and in that 2) the use of all inter-landmark distances that can be obtained from a set of landmarks results in many highly correlated measurements sharing common endpoints and redundantly measuring identical cranial aspects, which does not allow a comparison of integration magnitudes between blocks.

It was noticed that resampling with replacement produces a distribution of integration indices whose corresponding mean is always higher than the actual ICV or EV mean associated with a particular species or sex, and whose upper and lower 95% confidence limits are often higher than the ICV and the EV means of the actual sample. This is expected because resampling with replacement creates bootstrap replicatesin which single individuals are sampled more than once which leads to higher correlations.

An adjustment of the 95% confidence intervals of the ICVs and EVs was performed to include the actual sample mean within these intervals. The 95% confidence intervals adjustment proceeded by the calculation of the difference between the bootstrap sample mean and the actual mean for the ICVs, the EVs and the average trait CVs, followed by the subtraction of this difference from the 95% confidence interval obtained from independent resampling with replacement for 1000 iterations for each species or sex. The results from 95% confidence intervals adjustment are presented in the Supplementary Information and discussed in the “Results” section of the paper.

## Results

### I. Testing the Food-Material-Properties (*FMP*) and the Cranial Modularity (*CMOD*) Hypotheses: Results from PLS and Maximum Modularity Tests

#### 1. Facial integration magnitudes as measured by the RV coefficient

In all five capuchin species, the PLS analyses carried out on shape coordinates containing allometric shape change or on residuals from a regression on centroid size, be it in the framework of an oral-zygomatic modularity hypothesis or in the framework of a maximum facial modularity hypothesis, yield significant RV coefficients (used here as a multivariate generalization of the squared correlation coefficient), indicating that the permutation test against the null hypothesis of facial units independence is falsified, and that the face represents an integrated structure. Furthermore, the apelloid capuchins are characterized by higher between-block RV coefficients than *C. albifrons* and *C. olivaceus* under the oral-zygomatic PLS containing an allometric component or carried out on residuals regressed on centroid size, and under the maximum facial modularity PLS containing an allometric component.

**Figure 2:**
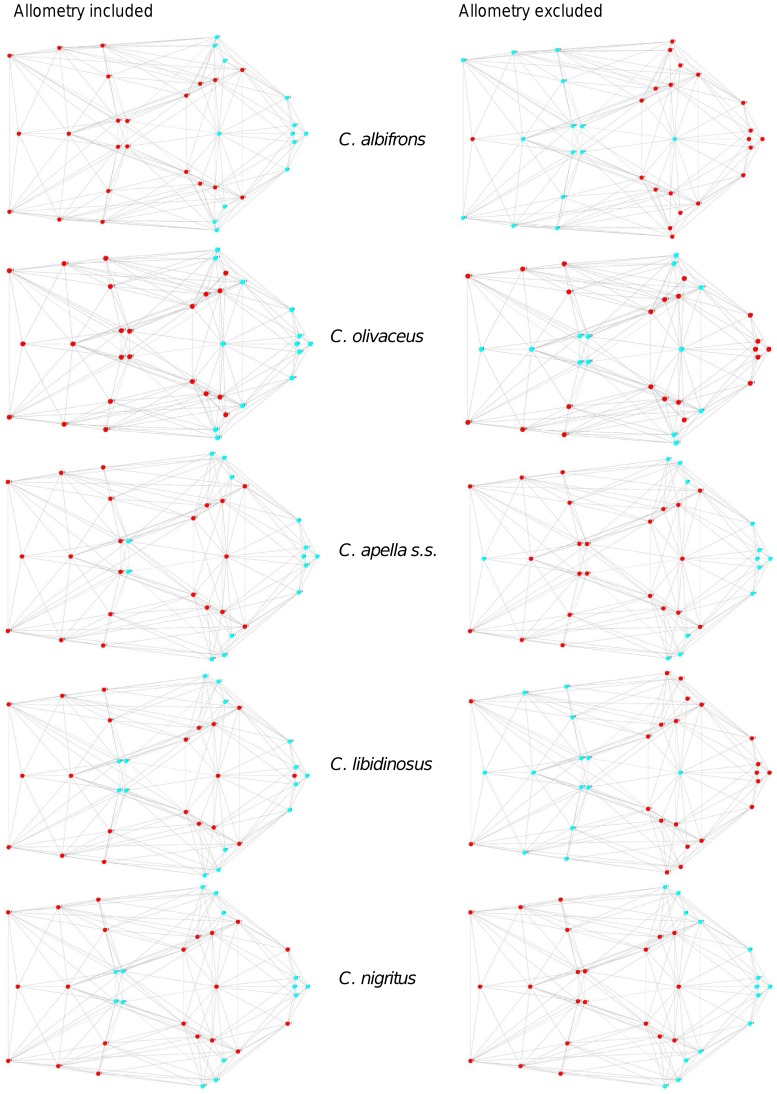
Two-block partitions yielding the minimum between-block covariance (maximum cranial modularity partition).

In particular, the overall strength of association between the oral and the zygomatic allometry-containing blocks of the three apelloid capuchins is higher than this of the gracile capuchins by approximately 0.20 (*Cebus apella s.s.*: RV = 0.59 (p<0.0001); *Cebus libidinosus*: RV = 0.57 (p<0.0001); *Cebus nigritus*: RV = 0.53 (p<0.0001); *Cebus albifrons*: RV = 0.39 (p<0.0001); *Cebus olivaceus*: RV = 0.38 (p<0.0001)) (Table 3). When allometric variation in the landmark coordinates is preserved, in all capuchins, the landmark subdivision yielding the smallest between-module integration includes a unit associating the three molar landmarks with the TMJs, and variably including landmarks near the zygomatic roots (especially zygomaxillare superior in all species but *C. apella s.s.*) (Figure 2). It is interesting to note that the molar-TMJ module of *C. olivaceus* contains all zygomatic landmarks, while this of *C. albifrons* contains only zygomaxillare superior. Contrarily to the situation in the apelloids, no rostral landmarks are strongly integrated with the molar-TMJ module in both gracile species. This observed independence of 1) the molar-TMJ module from the oral-zygomatic module in *C. albifrons* and 2) the molar-TMJ-zygomatic module from the rostral module in *C. olivaceus*, contrasts with the existing inter-relationships and coordinated shape change between the molar-TMJ, the rostral and the zygomatic modules observed in the apelloids, and indicates that, in the latter group, the face as a whole is a relatively integrated structure, at least under an allometric maximum modularity scenario.

**Table 3:** Distribution of facial integration magnitudes under an allometric PLS, as measured by the RV coefficient.

Species/sex	Face (oral-zygomatic)	Face (max. modularity)	Cranium (face-base)	Cranium (max. modularity)
*C. albifrons*	0.39	0.22	0.40	0.26
*C. olivaceus*	0.38	0.23	0.44	0.27
*C. apella s.s.*	0.59	0.40	0.64	0.42
*C. libidinosus*	0.57	0.33	0.62	0.36
*C. nigritus*	0.53	0.31	0.53	0.35

All RV coefficients are significant at p<0.0001.

When allometric shape change is preserved, the overall strength of between-block association under the maximum modularity hypothesis (not limited to spatially contiguous subsets of landmarks) is higher in the tufted capuchins compared to the gracile capuchins (RV (*C. apella s.s.*) = 0.40 (p<0.0001); RV (*C. libidinosus*) = 0.33 (p<0.0001); RV (*C. nigritus*) = 0.31(p<0.0001); RV (*C. albifrons*) = 0.22 (p<0.0001); RV (*C. olivaceus*) = 0.23 (p<0.0001)).

In a PLS based on an oral-zygomatic block subdivision after accounting for allometric variation, the RV coefficients of all apelloid capuchins are also higher than these of the gracile capuchins, although the numerical distinction between these groups is more limited in comparison with the allometric scenario (RV (*C. apella s.s.*) = 0.383 (p<0.0001); RV (*C. libidinosus*) = 0.404 (p<0.0001); RV (*C. nigritus*) = 0.41 (p 0.0001); RV (*C. albifrons*) = 0.33 (p<0.0001); RV (*C. olivaceus*) = 0.32 (p<0.0001)) (Table 4).

**Table 4:** Distribution of facial integration magnitudes under a non-allometric PLS, as measured by the RV coefficient.

Species/sex	Face (oral-zygomatic)	Face (max. modularity)	Cranium (face-base)	Cranium (max. modularity)
*C. albifrons*	0.33	0.24	0.20	0.196
	p<0.0001	p<0.0001	p = 0.0004	p = 0.0003
*C. olivaceus*	0.32	0.26	0.29	0.264
	p<0.0001	p<0.0001	p<0.0001	p<0.0001
*C. apella s.s.*	0.383	0.26	0.28	0.231
	p<0.0001	p<0.0001	p<0.0001	p<0.0001
*C. libidinosus*	0.404	0.30	0.27	0.263
	p<0.0001	p<0.0001	p<0.0001	p<0.0001
*C. nigritus*	0.41	0.24	0.24	0.224
	p<0.0001	p<0.0001	p = 0.0001	p<0.0001

**Figure 3:**
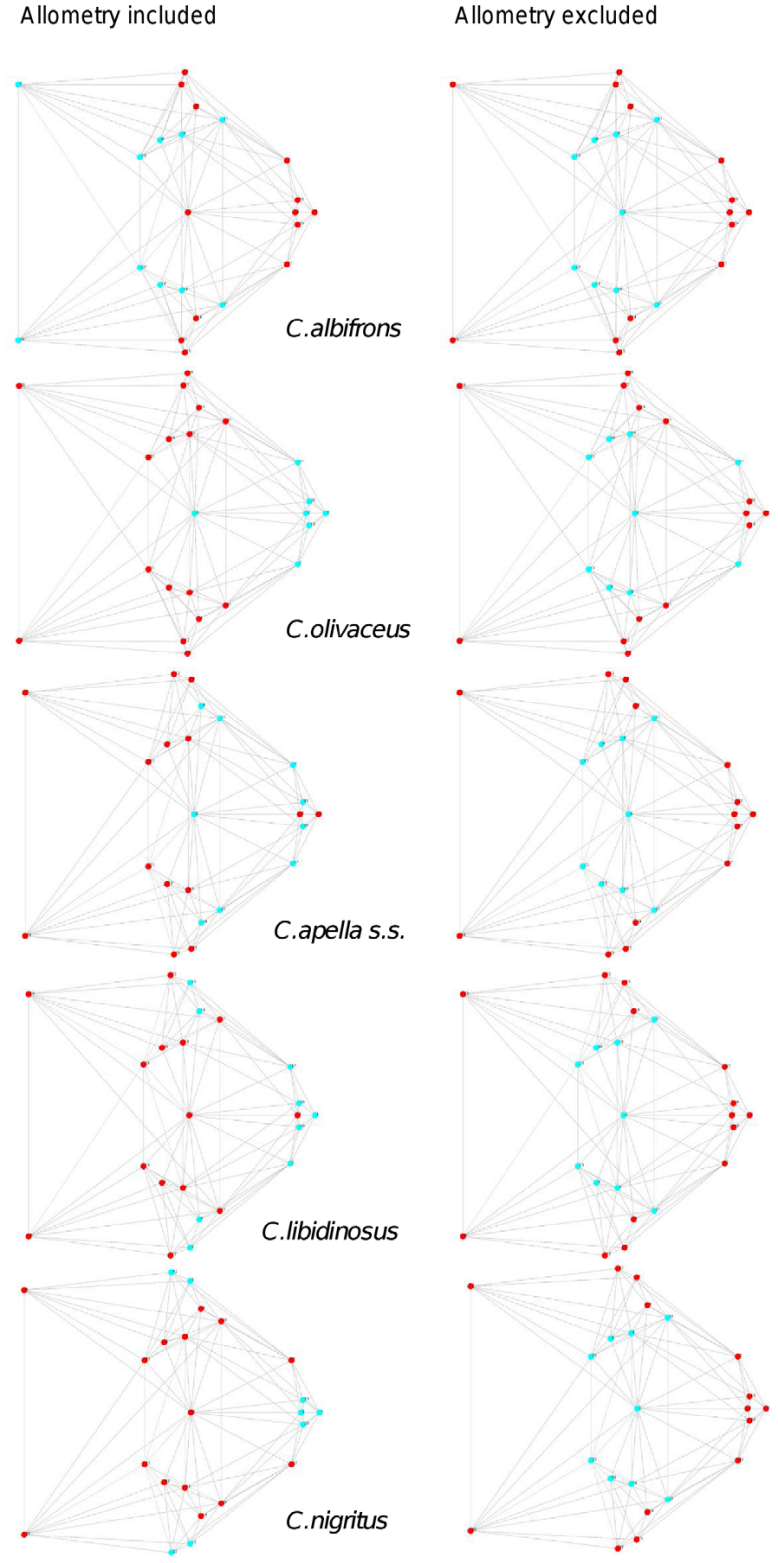
Sets of landmarks producing blocks that minimize the between-block covariance (maximum facial modularity partition).

When controlling for allometry, the landmark subdivision yielding the smallest between-module integration is identical in all species except for *C.olivaceus*, and includes a unit associating the three molar landmarks with the palatal landmark and zygomaxillare superior (Figure 3). In *C. olivaceus*, the three molar landmarks are associated with the palatal landmark and with the landmark at the meeting of the canine and the lateral incisor at the alveolus.

Thus, under both the allometric and the non-allometric scenarios, the oral region does not represent an individualized module, and instead can be broken down into two distinct modules: a rostral module and a molar module.

The non-allometric maximum modularity PLS analysis differs from the three previous analyses in that it yields RV coefficients that do not allow for a distinction between tufted and gracile capuchins (*C. apella s.s.*: RV = 0.26 (p<0.0001), *C. libidinosus*: RV = 0.30 (p<0.0001), *C. nigritus*: RV = 0.24 (p<0.0001), *C. albifrons*: 0.24 (p<0.0001), *C. olivaceus*: RV = 0.26 (p<0.0001)) (Table 4).

In summary, non-allometric shape variation in the oral and the zygomatic blocks is coordinated to a greater extent in the apelloids, but correlation magnitudes do not support the existence of integration differences based on dietary groups under a PLS subdivision yielding minimal between-module covariation.

#### 2. Cranial integration magnitudes as measured by the RV coefficient

Under a face-cranial base PLS landmark partition including allometric variation, the RV coefficients of all apelloid capuchins are also higher than these of the gracile capuchins. The overall strength of association between the facial and the basicranial blocks is higher in the tufted species and, for each species, equal or slightly higher than its corresponding oral-zygomatic RV (*C. apella s.s.*: RV = 0.64 (p<0.0001); *C. libidinosus*: RV = 0.62 (p<0.0001); *C. nigritus*: RV = 0.52(p<0.0001); *C. albifrons*: RV = 0.41(p<0.0001); *C. olivaceus*: RV = 0.44 (p<0.0001)) (Table 3).

The maximum cranial modularity partition for data preserving allometry, in all species, involves a block including the molar, the lateral basicranial and the foramen magnum landmarks, and a second block including all but one of the zygomatic landmarks, excluding in four of the five cases zygomaxillare superior and all (or most in the case of *C. nigritus*) of the rostral landmarks. The details of this pattern vary (Figure 2). In all tufted capuchins, some midline basicranial landmarks (sphenobasion or the anterior tips of the petrous pyramid) are excluded from the molar-cranial base block.

Under a maximum modularity PLS partition of coordinates preserving allometry, the RV coefficients of all apelloid capuchins are also higher than these of the gracile capuchins (*C. apella s.s.*: RV = 0.42 (p<0.0001); *C. libidinosus*: RV = 0.36 (p<0.0001); *C. nigritus*: RV = 0.35 (p<0.0001); *C. albifrons*: RV = 0.25 (p<0.0001); *C. olivaceus*: RV = 0.27 (p<0.0001)) (Table 3). When preserving allometric variation, the overall strength of association between the maximum modularity cranial blocks in all capuchin species is slightly higher than the RV coefficients under the facial maximum modularity subdivision.

The modular architecture obtained from the analysis of non-allometric data is more variable. In all tufted species, the anterior block always includes all rostral landmarks and all zygomatic landmarks (without zygomaxillare superior in *C. nigritus*), while the posterior block includes most or all basicranial landmarks (along with the molar landmarks in *C. apella s.s.* and *C. nigritus* but not in *C. libidinosus*). In *C. albifrons*, the maximum modularity analysis creates a relatively good partition between the face and the basicranium, with a block including all facial landmarks without the palatal landmark, and opisthion. An almost complete face-cranial base separation exists in *C. libidinosus* as well. In *C. olivaceus,* the face is not an individualized module; instead, all dental landmarks (molar and rostral), the inferior zygomatic root and all lateral basicranial landmarks are combined in a module, while all midline basicranial landmarks group with the rest of the zygomatic landmarks and the palatal landmark.

The strict face-cranial base PLS on non-allometric data produces RV coefficients, which are higher in all apelloid capuchins and *C. olivaceus* compared to that of the gracile *C. albifrons*. The overall strength of association between the facial and the basicranial blocks in the capuchin species is as follows: *C. apella s.s.*: RV = 0.28 (p<0.0001); *C. libidinosus*: RV = 0.27 (p<0.0001); *C. nigritus*: RV = 0.24 (p = 0.0001); *C. albifrons*: RV = 0.20 (p = 0.0004); *C. olivaceus*: RV = 0.29 (p<0.0001) (Table 4).

In each species, the non-allometric face-cranial base RVs are numerically very similar to the maximum modularity RVs, indicating that the face-cranial base landmark partition optimizes cranial modularity relatively well. The RV coefficients from the PLS, which maximizes modularity are statistically significant and slightly lower than these of the face-cranial base subdivision, and also indicate that *C. albifrons* has the least integrated face (*C. apella s.s.*: 0.231(p<0.0001), *C. libidinosus*: 0.263 (p<0.0001), *C. nigritus*: 0.224(p<0.0001), *C. albifrons*: 0.196(p = 0.0003), *C. olivaceus*: 0.264 (p<0.0001)). The proportions of partitions yielding RV coefficients smaller than the face-cranial base partitions are very small (*C. apella s.s.*: 0.0003, *C. libidinosus*: 0.000016, *C. nigritus*: 0.000008, *C. albifrons*: 0.000008, *C. olivaceus*: 0.000111).

The non-allometric face-basicranium RV values are numerically very similar to both the maximum cranial modularity RV values and the RV values produced by PLS on a non-allometric facial maximum modularity partition subdividing the face into a molar-palatal block and a rostral-zygomatic block. A comparison of RV coefficients derived from facial and cranial maximum modularity PLS analyses carried out on residuals from regression on centroid size, however indicates that between-block correlation is slightly stronger between the facial modules rather than between the cranial modules in all species but *C. olivaceus*. This finding suggests that the face is not a very strongly individualized module within the cranium, but suggests that the face may be slightly more integrated than the cranium as a whole. Analyses presented in the following sections of the article assessing statistical significance of differences in integration magnitudes between blocks allow firmer conclusions.

In addition to the suggested covariation between the facial and cranial measurements not containing allometry, allometric variation further increases integration between the face and the basicranium through correlation between the molar landmarks, the lateral basicranial landmarks and foramen magnum landmarks.

#### 3. Facial integration magnitudes of the allometric PLS1 patterns

**Figure 4:**
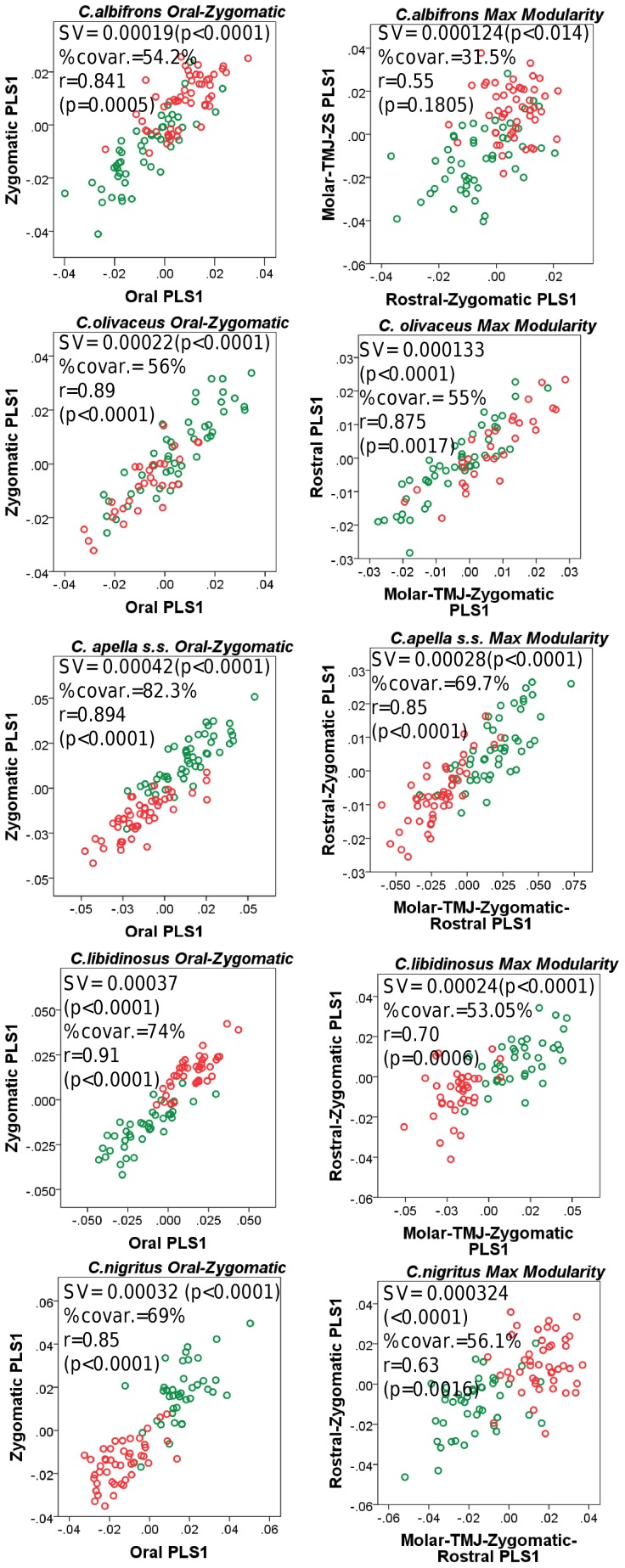
Distribution of the individuals’ loadings along the oral, zygomatic and maximum facial modularity allometric PLS1s. Abbreviations : SV = singular value, %covar. = percentage of between-block total squared covariance explained by the set of PLS1 axes, r = between-block correlation coefficient.

Under an allometric scenario, the first singular axes (PLS1) of the oral-zygomatic and maximum modularity PLS are significant in all species and summarize size-related shape covariation between the blocks in all species with the exception of *C. olivaceus* (Figure 4).

The strength of association between the oral and zygomatic blocks is very high and does not allow for a distinction on the basis of diet (*C. apella s.s.*: r = 0.894 (p = 0.0008), *C. libidinosus*: r = 0.91 (p = 0.0005), *C. nigritus*: r = 0.85 (p = 0.0022), *C. albifrons*: r = 0.841 (p = 0.0008), *C. olivaceus*: r = 0.89 (p = 0.0004)). The PLS1 of *C. albifrons* is characterized by a non-significant between-block correlation under a maximum modularity hypothesis, while *C. olivaceus* possesses the highest significant between-block PLS1 association (*C. apella s.s.*: r = 0.85 (p<0.0001), *C. libidinosus*: r = 0.70 (p = 0.0006), *C. nigritus*: r = 0.63 (p = 0.0016), *C. albifrons*: r = 0.55 (p = 0.1805), *C. olivaceus*: r = 0.875 (p = 0.0017)).

Despite the similarity in the magnitude of oral-zygomatic allometrically-driven correlations, PLS1 explains a smaller percentage of the total squared between-block covariance in the gracile capuchins (*C. apella s.s.*: 82.3%, *C. libidinosus*: 74%, *C. nigritus*: 69%, *C. albifrons*: 54.2%, *C. olivaceus*: 56%), indicating that allometric variation plays a more prominent role in the apelloids.

Under a maximum modularity hypothesis, the percentage of the total squared between-block covariance explained by PLS1 is smaller and particularly small in *C. albifrons* (*C. apella s.s.*: 69.7%, *C. libidinosus*: 53.05%, *C. nigritus*: 56.1%, *C. albifrons*: 31.5%, *C. olivaceus*: 55%). Thus, allometric shape change is less canalizing in *C. albifrons*’s face as it accounts for less covariation.

#### 4. Facial integration magnitudes of the non-allometric PLS1 patterns

**Figure 5:**
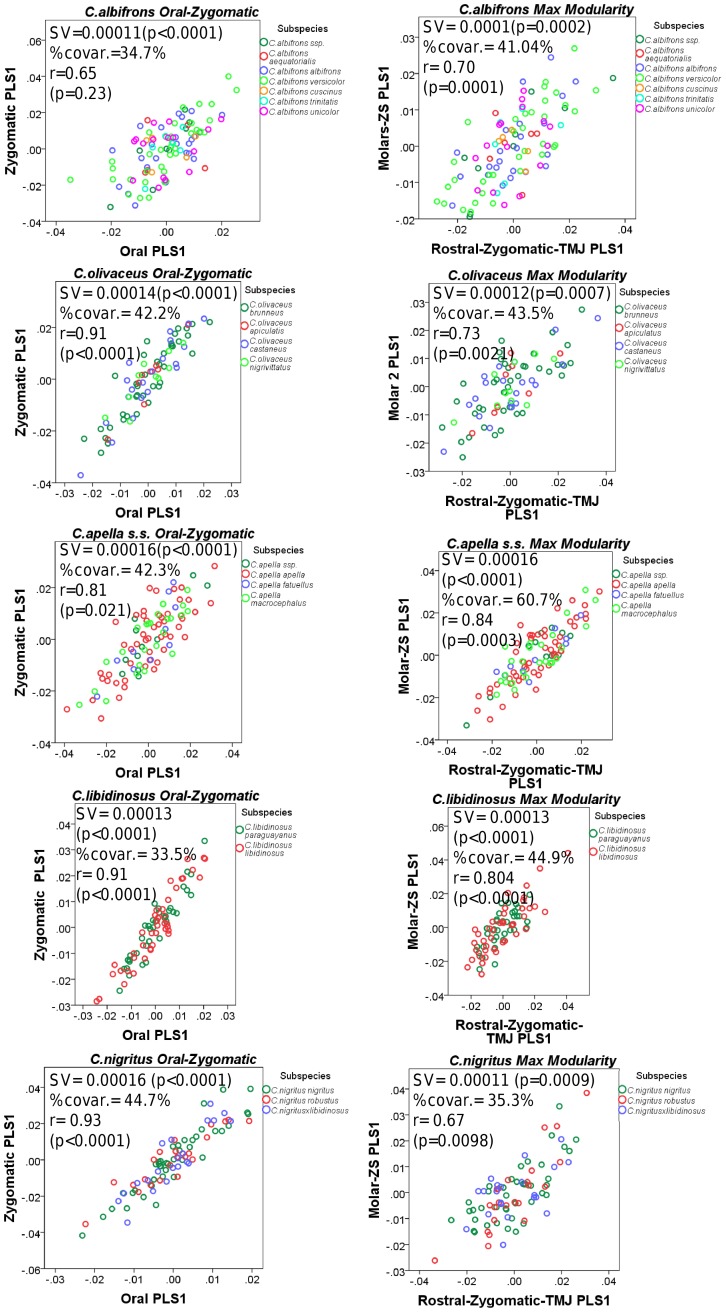
Distribution of the individuals’ loadings along the oral, zygomatic and maximum facial modularity non-allometric PLS1s. Abbreviations : SV = singular value, %covar. = percentage of between-block total squared covariance explained by the set of PLS1 axes, r = between-block correlation coefficient.

All non-allometric oral-zygomatic and maximum modularity PLS1 axes are statistically significant (Figure 5). In all apelloid species and in *C. olivaceus*, the oral-zygomatic correlation coefficients are quite high and higher than the allometric oral-zygomatic PLS1 correlations (*C. apella s.s.*: r = 0.81 (p = 0.021), *C. libidinosus*: r = 0.91 (p<0.0001), *C. nigritus*: r = 0.93 (p<0.0001), *C. albifrons*: r = 0.65 (p = 0.23), *C. olivaceus*: r = 0.91 (p<0.0001)) (Figure 5). Noticeable is the non-significant oral-zygomatic correlation coefficient of *C. albifrons*.

The maximum modularity correlation coefficients are statistically significant in all species, and lower than those of the allometric maximum modularity PLS1. *C. nigritus*, *C. albifrons* and *C. olivaceus* are characterized by the lowest correlations (*C. apella s.s.*: r = 0.84 (p = 0.0003), *C. libidinosus*: r = 0.804(p<0.0001), *C. nigritus*: r = 0.67 (p = 0.0098), *C. albifrons*: r = 0.70 (p = 0.0001), *C. olivaceus*: r = 0.73 (p = 0.0021)).

In all tufted species, the PLS1s under the non-allometric oral-zygomatic and maximum modularity partitions explain a percentage of the total squared between-block covariance that is approximately twice smaller than the allometric oral-zygomatic and maximum modularity PLS1s, respectively (Oral-zygomatic: *C. apella s.s.*: 42.3%, *C. libidinosus*: 33.5%, *C. nigritus*: 44.7%, *C. albifrons*: 34.7%, *C. olivaceus*: 42.2%; Maximum facial modularity.

*C. apella s.s.*: 60.7%, *C. libidinosus*: 44.9%, *C. nigritus*: 35.3%, *C. albifrons*: 41.04%, *C. olivaceus*: 43.5%). No numerical trend in the percentages of explained covariation distinguishes the tufted from the gracile capuchins.

#### 5. Cranial integration magnitudes of the allometric PLS1 patterns

**Figure 6:**
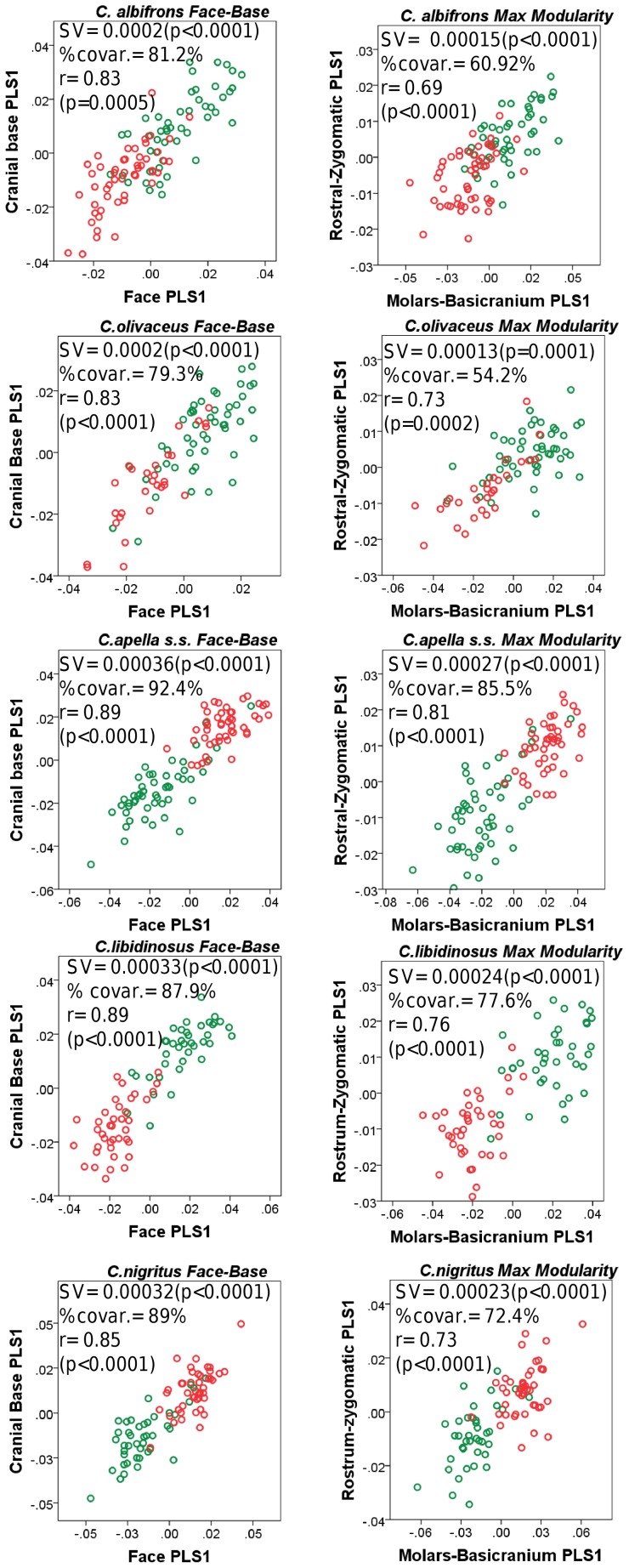
Distribution of the individuals’ loadings along the facial, basicranial and maximum cranial modularity allometric PLS1s. Abbreviations : SV = singular value, %covar. = percentage of between-block total squared covariance explained by the set of PLS1 axes, r = between-block correlation coefficient.

The PLS1 axes under both the face-cranial base and the maximum modularity scenarios of all species are significant (Figure 6). The face-cranial base and the maximum modularity PLS1 correlation coefficients are statistically significant and high in all species, and slightly higher in the apelloids or in the apelloids - *C.olivaceus* group (Face-Cranial Base PLS1: *C. albifrons:* r = 0.83 (p = 0.0004), *C. olivaceus:* r = 0.83 (p<0.0001), *C. apella:* r = 0.89 (p<0.0001), *C. libidinosus:* r = 0.89 (p<0.0001), *C. nigritus:* r = 0.85 (p<0.0001), Maximum modularity PLS1: *C. albifrons*: r = 0.69 (p<0.0001), *C. olivaceus:* r = 0.73 (p = 0.0002), *C. apella:* r = 0.81 (p<0.0001), *C. libidinosus:* r = 0.76 (p<0.0001), *C. nigritus:* r = 0.73 (p<0.0001)).

The proportion of explained squared between-block covariance is slightly higher in the apelloids (Face-Cranial base: *C. albifrons* 81.2%, *C. olivaceus* 79.3%, *C. apella* 92.4%, *C. libidinosus* 87.9%, *C. nigritus* 89%, Maximum modularity: *C. albifrons:* 60.92%, *C. olivaceus:* 54.2%, *C. apella:* 85.5%, *C. libidinosus:* 77.6%, *C. nigritus:* 76.5%) (Figure 6).

#### 6. Cranial integration magnitudes of the non-allometric PLS1 patterns

**Figure 7:**
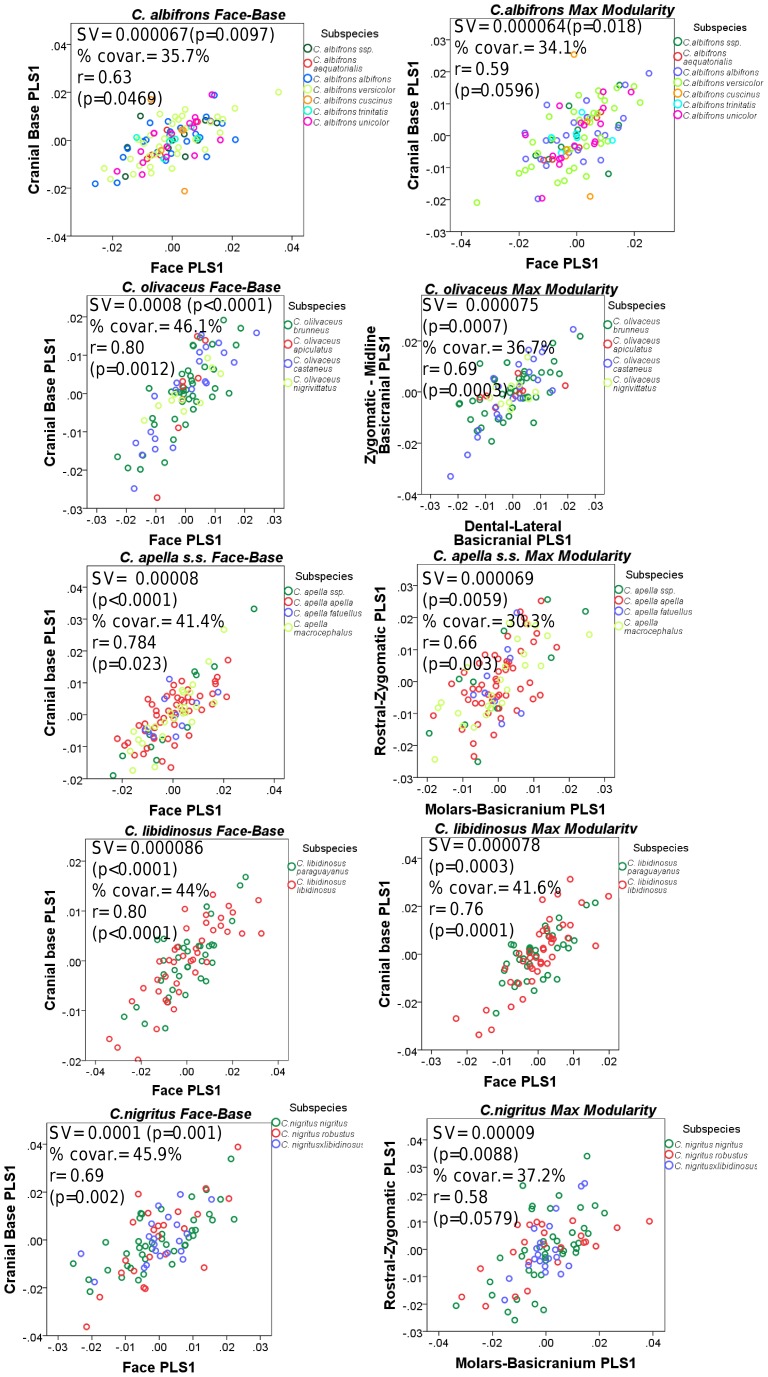
Distribution of the individuals’ loadings along the facial, basicranial and maximum cranial modularity non-allometric PLS1s. Abbreviations : SV = singular value, %covar. = percentage of between-block total squared covariance explained by the set of PLS1 axes, r = between-block correlation coefficient.

The non-allometric PLS1 axes of all species under both PLS partitions are significant (Figure 7). Compared to the other capuchins, the non-allometric face-cranial base PLS1 of *C. albifrons* explains a smaller percentage of between-block covariance (*C. albifrons:* 35.7%, *C. olivaceus:* 46.1%, *C. apella s.s.*: 41.4%, *C. libidinosus:* 44%, *C. nigritus:* 45.9%) and has the lowest between-block correlation coefficient (*C. albifrons:* r = 0.63 (p = 0.0479), *C. olivaceus:* r = 0.80 (p = 0.0012), *C. apella s.s.*: r = 0.784 (p = 0.023), *C. libidinosus:* r = 0.80 (p<0.0001), *C. nigritus:* r = 0.69 (p = 0.0017)).

Under the non-allometric maximum cranial modularity scenario, the proportion of explained between-block covariance contained in the PLS1axes does not differentiate any of the gracile species from the rest of the capuchins (*C. albifrons:* 34.1%, *C. olivaceus:* 36.7%, *C. apella:* 30.3%, *C. libidinosus:* 41.6%, *C. nigritus:* 39%). The non-allometric PLS1 between-block correlation coefficient is not statistically significant at the 0.05 level in *C. nigritus* and is marginally significant in *C. albifrons* (*C. albifrons*: 0.59 (p = 0.0596), *C. olivaceus:* 0.69 (p = 0.0003), *C. apella:* 0.66 (p = 0.003), *C. libidinosus:* 0.78 (p = 0.0001), *C. nigritus:* 0.58 (p = 0.058)). The high correlation coefficient of *C. olivaceus* is not surprising, because each of its blocks includes many landmarks from both the face and the cranial base. *C. libidinosus* and *C. albifrons* are characterized by very similar maximum modularity partitions in which the face and the cranial base are well distinct. Yet, consistent with the H_(FMP1)_ hypothesis, *C. libidinosus*’ correlation coefficient is higher by 0.18 units. Thus, compared to *C. libidinosus*, *C. albifrons* has a more modular cranium. Similarly, the maximum modularity partitions of *C. nigritus* and *C. apella s.s.* are very similar and both include all molar landmarks, the palatal landmarks and all basicranium landmarks with the exception of opisthion. Yet, between-block correlations indicate that *C. nigritus*, compared to *C. apella s.s.*, is characterized by less coordinated variation between the molar-basicranial unit and the rostral-zygomatic unit. *C. nigritus* has been reported to include a high proportion (up to 73.6%) of leaves in its diet during food scarcity periods, which might imply that selective pressures related to the opening of hard-shelled nuts with the anterior dentition and associated biomechanical constrains on facial anatomy are lower in this species [113], [115].

#### 7. The role of allometry in facial PLS1

Shape change contained in the oral and zygomatic PLS1 correlates significantly (p<0.0001) with centroid size in all species. Furthermore, centroid size has a higher predictive power on the variation in each block in the tufted species. (Oral, % predicted: *C. apella s.s.* = 60.76%; *C. libidinosus* = 70.3%; *C. nigritus* = 63.5%; *C. albifrons* = 49.3%; *C. olivaceus* = 50.5%); Zygomatic, % predicted: *C. apella s.s.* = 66.32%; *C. libidinosus* = 79.5%; *C. nigritus* = 63.7%; *C. albifrons* = 61.9%; *C. olivaceus* = 50%).

Under a maximum modularity scenario which minimizes allometrically-driven between-block correlation, in all species except for *C. olivaceus*, the PLS1 axis of each block is significantly correlated with centroid size, and in all instances the molar-TMJ module contains a larger proportion, approximately two-thirds of the allometric variation, than the module containing the rest of the landmarks (% of variation predicted by centroid size: *C. apella s.s.*: 69%(p<0.0001), *C. libidinosus*:77% (p<0.0001), *C. nigritus*: 66% (p<0.0001), *C. albifrons*: 60% (p<0.0001)). In *C. olivaceus*, centroid size predicts only 6.6% (p = 0.023) of the total variation contained in the PLS1 scores of the molar-TMJ-zygomatic module, while 36% (p<0.0001) of the shape change contained in the PLS2 scores of the molar-TMJ-zygomatic block is allometric. The *C. olivaceus*’ maximum facial modularity PLS2 explains a markedly smaller proportion of the total squared covariance contained in the landmark coordinates compared to the maximum facial modularity PLS1 (17% in PLS2 versus 55% in PLS1), but its shape change is identical to the allometric change contained in the PLS1 axes of the other species (see last section).

In all species except for *C. olivaceus*, shape change contained in the rostral-zygomatic PLS1 is also partly allometric and explains only approximately one third of the variation (% variation prediction by centroid size: *C. apella s.s.*: 37% (p<0.0001), *C. libidinosus*: 32% (p<0.0001), *C. nigritus*: 33% (p<0.0001), *C. albifrons*: 36% (p<0.0001)). In *C. olivaceus*, centroid size does not predict variation in the PLS1 scores of the rostral module (% predicted: 2.3% (p = 0.1813)). The PLS2 of the rostral module of *C. olivaceus* does not contain allometric variation either (% predicted: 1.11%, p = 0.36).

The results presented above demonstrate (a) that allometry plays a much less important role in *C. olivaceus* than in the other examined capuchins and (b) that the molar -TMJ module contains two times more size-related shape change than the rostrum. In fact, the allometric molar-TMJ shape change is among the most pronounced shape changes in the face even under the maximum modularity hypothesis PLS analysis.

#### 8. The role of allometry in cranial PLS1

In a face-cranial base partition, in all species, approximately two thirds to three fourths of the PLS1 variation in both blocks is allometric in origin. The PLS1 axes of the faces of the tufted capuchins appear to be slightly more accurately predicted by centroid size than the PLS1 axes of the faces of the untufted species (*C. albifrons*: 68.3%, *C.olivaceus*: 60.3%, *C. apella*: 74.2%, *C. libidinosus*: 82.4%, *C. nigritus*, 72.1%, (p<0.0001)). The proportions of variation in the PLS1 of the cranial base block explained by centroid size do not allow for a distinction between apelloids and graciles (*C. albifrons*: 68.6%, *C. olivaceus*: 59.5%, *C. apella*: 71.6%, *C. libidinosus*: 79.6%, *C. nigritus*, 59.7%, (p<0.0001)).

Under a maximum modularity scenario, in all species, 66 to 85 per cent of the variation contained in the PLS1 of the molar-cranial base block is allometric in origin (% predicted by centroid size: *C. albifrons*: 74.44%, *C. olivaceus*: 66.8%, *C. apella s.s.*: 77.9%, *C. libidinosus*: 84.8%, *C. nigritus*: 69.2%, (p<0.0001), while only between 35 and 60 per cent of the variation contained in the rostral-zygomatic block can be accurately predicted by centroid size (% predicted by centroid size: *C. albifrons*: 36.1%, *C. olivaceus*: 59.5%, *C. apella s.s.*: 54%, *C. libidinosus*: 56.6%, *C. nigritus*, 38.1%, p<0.0001). In all species with the exception of *C. olivaceus*, the molar-cranial base block contains at least 24% more allometric variation than the rostral-zygomatic block.

The overall allometric variation in the cranial 3D landmark coordinates also indicates that allometric change is more canalizing in the tufted species (*C. apella s.s.:* 26.7%, *C. libidinosus:* 27.2%, *C. nigritus:* 22%, *C. albifrons:* 17.44%, *C. olivaceus:* 16.5% (p<0.0001)).

#### 9. Comparison of facial and cranial allometric and non-allometric PLS1 and PC1

In contrast with the high percentage of the total between-block covariation of PLS1 explained by allometry, allometry in the first facial and cranial PC1 axes accounts for a small percentage of overall variation in the dataset that is smaller in the gracile species (Table 5). Compared to the allometric facial PC1, a larger proportion of variation contained in the cranial PC1 is predicted by centroid size in all species (Table 5). Interspecific variation in the predictive power of centroid size on variation in the coordinates contained in the facial and cranial PC1s confirms the less prominent role of allometry in *C. olivaceus*.

**Table 5:** Variance explained and allometry contained in the first principal component axis of the facial and cranial configuration.

Species	Facial PC1			Cranial PC1		
	Allometric		Non-allometric	Allometric		Non-allometric
	Variance explained	Predicted from CS	Variance explained	Variance explained	Predicted from CS	Variance explained
*C.albifrons*	17.37%	64.4%	18.1%	22.7%	73%	12.9%
*C.olivaceus*	18.1%	45.4%	17.4%	23.5%	66.6%	13.4%
*C. apella s.s.*	30.4%	66.4%	16.3%	34.1%	77.6%	12.2%
*C. libidinosus*	27.3%	75.6%	15.3%	31.6%	85.1%	14.9%
*C. nigritus*	25.1%	65.2%	17.1%	29.8%	70.6%	15.2%

All percentages of variance explained by centroid size are significant at the p<0.0001 level.

Abbreviations: CS: centroid size.

The variance explained by the non-allometric facial PC1 is lower than the variance explained by the allometric facial PC1 in the apelloids but not in the gracile species. Variation contained in the non-allometric cranial PC1 is lower than variation contained by the allometric cranial PC1 in all species.

The allometric cranial PC1 accounts for slightly more variation than its corresponding facial PC1, while the non-allometric cranial PC1 is slightly lower than the non-allometric facial PC1.

#### 10. PLS2: allometric and non-allometric facial integration magnitudes

The PLS2 axes of all species are significant (under the allometric and the non-allometric oral-zygomatic and maximum modularity partition) (Table 6). All species possess very high and significant oral-zygomatic PLS2 correlations obtained from an analysis preserving allometry (note that it is the first axis which contains the allometric shape change) (*C. albifrons*: 0.93 (p<0.0001), *C. olivaceus*: 0.82 (p<0.0001), *C. apella s.s.*: 0.93 (p = 0.0052), *C. libidinosus*: 0.96 (p = 0.0005), *C. nigritus*: 0.93 (p<0.0001)). These high correlations are combined with relatively low proportions of explained squared covariance by the PLS2 axes (Table 6). Between-block correlations are lower under an allometric maximum modularity scenario and non-significant in *C. olivaceus* (*C. albifrons*: 0.80 (p<0.0001), *C. olivaceus*: 0.50 (p = 26), *C. apella s.s.*: 0.76 (p = 0.0052), *C. libidinosus*: 0.708 (p = 0.015), *C. nigritus*: 0.74 (p = 0.0003)).

**Table 6:** Distribution of facial integration magnitudes as measured by the between-block correlation coefficient of PLS2.

Species/sex	Allometric face (oral-zygomatic PLS 2)				Non-allometric face (oral-zygomatic PLS 2)				
	SV		% Cov.	r	SV		% Cov.		r
*C. albifrons*	0.000106		16.34%	0.93	0.000095		24.1%		0.65
	p<0.0001			p<0.0001	p<0.0001				p = 0.095
*C. olivaceus*	0.000137		22.15%	0.82	0.0001		21.9%		0.67
	p<0.0001			p = 0.0052	p<0.0001				p = 0.1885
*C. apella s.s.*	0.000123		7.2%	0.93	0.000128		28%		0.93
	p = 0.0001			p<0.0001	p<0.0001				p = 0.0004
*C. libidinosus*	0.000134		9.9%	0.96	0.00012		27.2%		0.74
	p<0.0001			p = 0.0005	p<0.0001				p = 0.27
*C. nigritus*	0.00015		15%	0.93	0.000125		28.1%		0.94
	p<0.0001			p<0.0001	p<0.0001				p<0.0001
**Species/sex**	**Allometric face (maximum modularity PLS2)**				**Non-allometric face (maximum modularity PLS2)**				
	**SV**	**% Cov.**		**r**	**SV**	**% Cov.**		**r**	
*C. albifrons*	0.00012	29.9%		0.80	0.000069	18%		0.67	
	p<0.0001			p<0.0001	p = 0.0002			p = 0.0018	
*C. olivaceus*	0.00073	16.5%		0.50	0.00008	19.3%		0.67	
	p = 0.017			p = 0.26	p<0.0001			p = 0.007	
*C. apella s.s.*	0.000106	10.14%		0.76	0.000084	18%		0.67	
	p<0.0001			p<0.0001	p = 0.0001			p = 0.009	
*C. libidinosus*	0.000148	20.9%		0.708	0.000095	23.16%		0.69	
	p<0.0001			p = 0.015	p<0.0001			p = 0.007	
*C. nigritus*	0.00016	24.4%		0.74	0.000094	24.56%		0.63	
	p<0.0001			p = 0.0003	p<0.0001			p = 0.1	

Legend: SV: singular value, r: between-block correlation coefficient,

% Cov.: percentage of squared between-block covariance explained by this axis.

Under a non-allometric scenario, oral-zygomatic PLS2 correlations are not statistically significant at the 0.05 level in the two gracile species and in *C. libidinosus* (*C. albifrons*: 0.65 (p = 0.095), *C. olivaceus*: 0.67 (p = 0.19), *C. apella s.s.*: 0.93 (p = 0.0004), *C. libidinosus*: 0.74(p = 0.27), *C. nigritus*: 0.94 (p<0.0001)).

Under a non-allometric maximum modularity scenario, the PLS2 between-block correlations are numerically very similar in all species (*C. albifrons*: 0.67 (p = 0.0018), *C. olivaceus*: 0.67 (p = 0.007), *C. apella s.s.*: 0.67 (p = 0.009), *C. libidinosus*: 0.69(p = 0.007), *C. nigritus*: 0.63 (p = 0.1)).

#### 11. PLS2: allometric and non-allometric cranial integration magnitudes

In comparison with the facial PLS2, the allometric face-cranial base PLS2 axes of all species with the exception of *C. libidinosus* are not significant (Table 7). Furthermore, the allometric face-cranial base PLS2 correlations of *C. apella s.s.* and *C. libidinosus* are the only statistically significant correlations at the 0.05 level, while the correlation of *C. albifrons* is marginally significant and lower than these of the aforementioned apelloids (*C. albifrons*: 0.68 (p = 0.056), *C. olivaceus*: 0.71 (p = 0.13), *C. apella s.s.*: 0.80 (p = 0.0009), *C. libidinosus*: 0.80 (p = 0.0001), *C. nigritus*: 0.60 (p = 0.118)). Similarly to the results from the face, the allometric maximum cranial modularity PLS2s correlations are numerically very similar in all species (*C. albifrons*: 0.65 (p = 0.0047), *C. olivaceus*: 0.68 (p = 0.0017), *C. apella s.s.*: 0.70 (p<0.0001), *C. libidinosus*: 0.65(p = 0.024), *C. nigritus*: 0.59 (p = 0.166)).

**Table 7:** Distribution of cranial integration magnitudes as measured by the between-block correlation coefficient of PLS2.

Species/sex	Allometric cranium (face-base PLS 2)			Non-allometric cranium (face-base PLS 2)		
	SV	% Cov.	r	SV	% Cov.	r
*C. albifrons*	0.000054	6%	0.68	0.00006	24.33%	0.73
	p = 0.38		p = 0.056	p = 0.0006		p = 0.002
*C. olivaceus*	0.000057	6.65%	0.71	0.00005	18.9%	0.73
	p = 0.52		p = 0.13	p = 0.0008		p = 0.084
*C. apella s.s.*	0.000064	2.89%	0.80	0.00006	20.83%	0.75
	p = 0.734		p = 0.0009	p<0.0001		p = 0.02
*C. libidinosus*	0.00009	6.48%	0.80	0.00005	17.3%	0.067
	p = 0.009		p = 0.0001	p = 0.009		p = 0.004
*C. nigritus*	0.000071	4.3%	0.60	0.00007	22.5%	0.591
	p = 0.671		p = 0.118	p = 0.0003		p = 0.3
**Species/sex**	**Allometric cranium(maximum modularity PLS2)**			**Non-allometric cranium (maximum modularity PLS2)**		
	**SV**	**% Cov.**	**r**	**SV**	**% Cov.**	**r**
*C. albifrons*	0.000062	12.7%	0.67	0.00006	23.3%	0.65
	p = 0.0011		p = 0.002	p = 0.0011		p = 0.0047
*C. olivaceus*	0.000069	15.84%	0.70	0.00006	25.1%	0.68
	p = 0.0004		p = 0.0114	p<0.0001		p = 0.0017
*C. apella s.s.*	0.00007	5.55%	0.70	0.00006	23.6%	0.70
	p = 0.0007		p = 0.0004	p<0.0001		p<0.0001
*C. libidinosus*	0.000094	1.6%	0.72	0.00005	18.6%	0.65
	p<0.0001		p<0.0001	p = 0.004		p = 0.024
*C. nigritus*	0.000084	9.3%	0.68	0.000075	25.4%	0.59
	p = 0.0001		p<0.0001	p<0.0001		p = 0.166

Legend: SV: singular value, r: between-block correlation coefficient,

% covariance: percentage of squared between-block covariance explained by this axis.

The between-block correlation coefficients of the non-allometric PLS2s range between approximately 0.60 and 0.75, are significant in most species, and do not exhibit a difference in values that corresponds to dietary groupings (Table 7).

### II. Testing the *FMP* Hypotheses: Integration Indices (ICVs) Compared at a Common Level of Sampled Population Variation

The results of the interspesific comparison of the adjusted 95% confidence intervals of the ICVs and the mean CVs are equivalent to these obtained from the unadjusted ICV/mean CV comparison (Tables S1, S2, S3, S4, S5, S6, S7, Figures S1–S2), although minor differences are noticeable and reported below.

**Figure 8:**
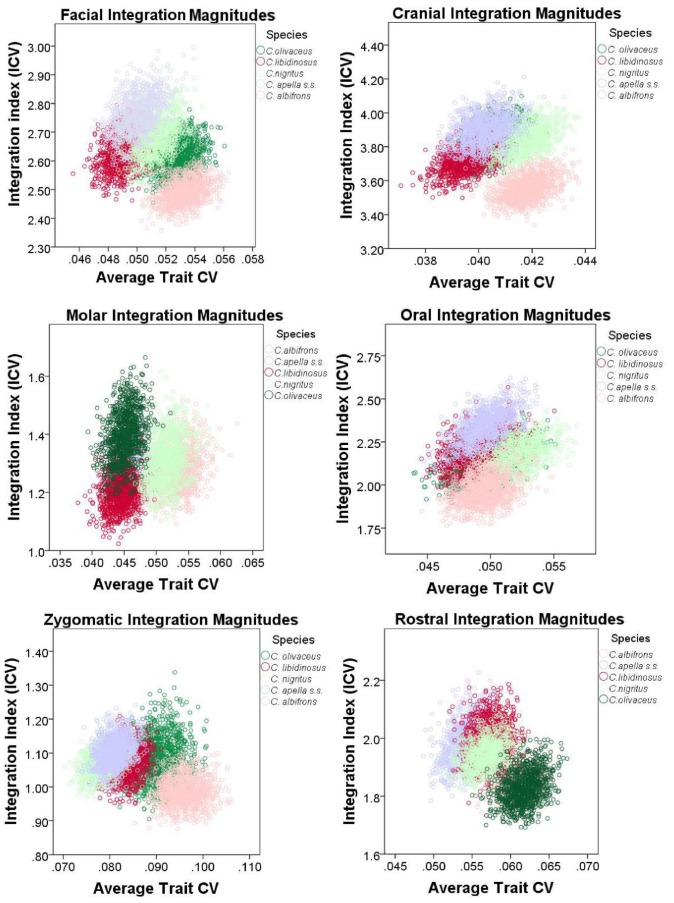
Inter-specific variation in integration indices values (ICVs) with regard to sample average trait CVs.

The H_(FMP1)_ hypothesis is supported by the interspecific comparison of the bootstrap-generated ranges for the integration indices (ICVs) and average trait CV values and the assessment of their 95% confidence intervals (95% CIs), obtained from the eigen analysis of 78 facial inter-landmark distances. *C. albifrons*’ and *C. olivaceus*’ mean CV 95% CIs encompass higher values while their ICV 95% CIs contain lower interval boundaries compared to the apelloids. Yet, in all between-species pair comparisons except for the *C. apella s.s.* – *C. albifrons* pair, these 95% CIs overlap (Table 8, Figure 8, Table S1, Figure S1). Comparisons of the unadjusted facial ICV 95% CIs at an identical level of average CV (CV = 0.051) indicate that the ICV range of *C. albifrons* is non-overlapping with the ranges of the apelloids (and non-overlapping with the ranges of *C. apella s.s.* and *C. libidinosus* in the case of the adjusted intervals), while the facial ICV range of *C. olivaceus* overlaps with these of *C. libidinosus* and *C. nigritus* but not with this of *C. apella s.s.*

**Table 8:** Inter-specific variation in facial ICV integration indices.

Species	95% CI ICV	95% CI Mean CV	Actual ICV	Actual mean CV	ICV at a mean CV of 0.051
*C. albifrons*	2.414–2.577	0.051–0.0554	2.385	0.053	2.43–2.52
*C. olivaceus*	2.516–2.721	0.0509–0.0548	2.49	0.049	2.50–2.67
*C. apella s.s.*	2.636–2.888	0.0479–0.052	2.653	0.05	2.67–2.92
*C. libidinosus*	2.519–2.743	0.0472–0.0508	2.498	0.049	2.6–2.73
*C. nigritus*	2.557–2.817	0.0493–0.0533	2.56	0.052	2.53–2.81

The ICV values obtained from the analysis of the 30 inter-landmark distances of the oral block are in part consistent with the predictions of the H_(FMP1)_ hypothesis. At a mean CV value of 0.05 *C. albifrons*’ ICV range is non-overlapping with this of *C. apella s.s.*, while the ranges of the rest of the species extensively overlap (Table 9, Figure 8, Table S2, Figure S1). The ICV values from the actual samples are associated with a mean CV level equal to or slightly higher than 0.05, and also suggest that *C. albifrons* has particularly low oral integration magnitudes, that *C. olivaceus* is characterized by an average oral ICV lower than this of all apelloids, and that *C. apella s.s.* has an average oral ICV markedly higher than these of all other capuchin species.

**Table 9:** Inter-specific variation in oral ICV integration indices.

Species	95% CI ICV	95% CI Mean CV	Actual ICV	Actual mean CV	ICV at a mean CV of 0.05
*C. albifrons*	1.816–2.13	0.0467–0.0524	1.92	0.0498	1.8–2.16
*C. olivaceus*	1.975–2.297	0.046–0.0531	2.07	0.0498	1.97–2.32
*C. apella s.s.*	2.164–2.528	0.0466–0.0526	2.32	0.0499	2.19–2.53
*C. libidinosus*	2.0154–2.39	0.046–0.0525	2.13	0.049	1.98–2.42
*C. nigritus*	2–2.343	0.491–0.055	2.13	0.052	1.95–2.28

The comparative assessment of 10 zygomatic inter-landmark distances does not allow for a comparison of ICV values at a common mean CV level, yet it indicates that *C. albifrons* is again associated with the lowest 95% CI ICV range and with the highest mean CV 95% CI range (Table 10, Figure 8, Table S3, Figure S1). Identical results are obtained from the comparison of the actual ICV and mean CV values. *C. apella s.s.* is associated with the highest actual ICV value and with one of the lowest actual mean CV values.

**Table 10:** Inter-specific variation in zygomatic ICV integration indices.

Species	95% CI ICV	95% CI Mean CV	Actual ICV	Actual mean CV
*C. albifrons*	0.909–1.077	0.09–0.1027	0.952	0.097
*C. olivaceus*	0.972–1.22	0.083–0.0976	1.045	0.09
*C. apella s.s.*	1.024–1.193	0.0763–0.086	1.08	0.082
*C. libidinosus*	0.99–1.155	0.0785–0.0884	1.036	0.0084
*C. nigritus*	1–1.4	0.0736–0.0838	1.048	0.08

No potential diet-driven trend is observed when examining interspecific variation in integration magnitudes of the molar block (Figure 8, Table 11, Figure S1, Table S4). Integration in the rostral-zygomatic block (abbreviated in the diagrams as “rostral”) is significantly higher in *C. apella s.s.* compared to both gracile species. The gracile species’ rostral-zygomatic ICVs 95% CIs are lower than but slightly overlap with these of *C. nigritus* and *C. libidinosus* (Figure 8, Table 12, Figure S1, Table S5). These results are consistent with the H_(FMP1)_ hypothesis.

**Table 11:** Inter-specific variation in molar ICV integration indices.

Species	95% CI ICV	95% CI Mean CV	Actual ICV	Actual mean CV	ICV at a mean CV of 0.048
*C. albifrons*	1.15–1.427	0.0494–0.0579	1.264	0.054	1.13–1.34
*C. olivaceus*	1.233–1.564	0.0416–0.0485	1.375	0.0455	1.30–1.59
*C. apella s.s.*	1.191–1.426	0.043–0.04896	1.278	0.0463	1.19–1.43
*C. libidinosus*	1.093–1.342	0.041–0.048	1.167	0.045	1.14–1.33
*C. nigritus*	1.162–1.48	0.0477–0.05606	1.286	0.052	1.14–1.41

**Table 12:** Inter-specific variation in rostral-zygomatic ICV integration indices.

Species	95% CI ICV	95% CI Mean CV	Actual ICV	Actual mean CV	ICV at a mean CV of 0.058
*C. albifrons*	1.769–1.917	0.0579–0.064	1.787	0.062	1.77–1.88
*C. olivaceus*	1.727–1.948	0.058–0.0653	1.764	0.062	1.72–1.87
*C. apella s.s.*	1.854–2.13	0.0506–0.0578	1.939	0.0547	1.97–2.13
*C. libidinosus*	1.876–2.126	0.054–0.0605	1.925	0.058	1.83–2.15
*C. nigritus*	1.853–2.045	0.053–0.0603	1.957	0.057	1.85–2.06

The distribution of the cranial ICV values with regard to the mean CV values obtained from the eigen analysis of 190 inter-landmark cranial distances indicates that not only the face of *C. albifrons*, but also its cranium is less integrated than the cranium of any other examined capuchin, and specifically of *C. apella s.s.*

An interspecific comparison of the ICV 95% CI values at a mean CV level of 0.041 indicates that *C. albifrons* has the lowest ICV range non-overlapping with this of any other species except for a slight overlap with *C. nigritus,* while *C. olivaceus’* integration index range is very similar to this of the tufted species (Table 13, Figure 8, Table S6, Figure S1). The actual mean CV and ICV values also indicate that *C. albifrons* is associated with the lowest ICV values, while *C. apella s.s.* has the highest ICV values.

**Table 13:** Inter-specific variation in cranial ICV integration indices.

Species	95% CI ICV	95% CI Mean CV	Actual ICV	Actual mean CV	ICV at a mean CV of 0.041
*C. albifrons*	3.433–3.698	0.04025–0.0434	3.37	0.042	3.39–3.65
*C. olivaceus*	3.682–4.003	0.0394–0.0417	3.63	0.040	3.72–4.04
*C. apella s.s.*	3.738–4.073	0.0389–0.0415	3.71	0.040	3.77–4.12
*C. libidinosus*	3.574–3.891	0.0383–0.041	3.5	0.039	3.72–3.91
*C. nigritus*	3.685–4	0.0404–0.043	3.63	0.042	3.64–3.98

**Figure 9:**
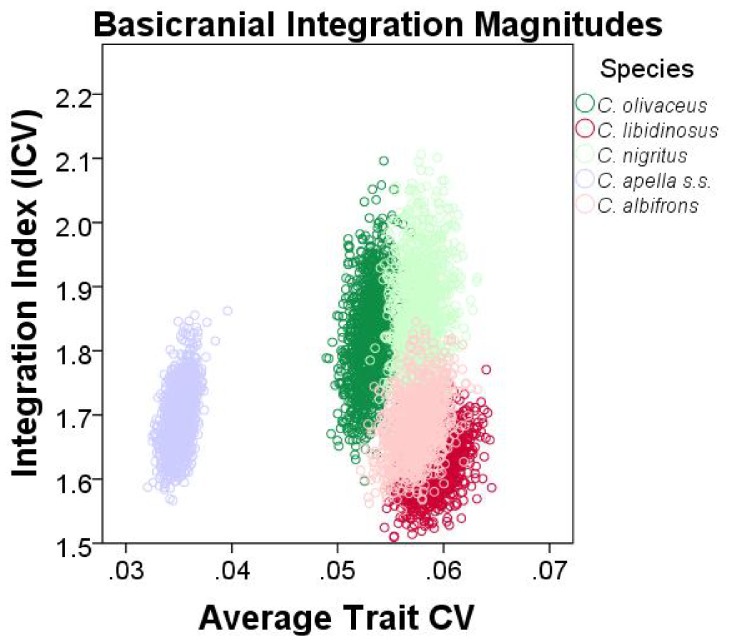
Inter-specific variation in basicranial integration indices values (ICVs) with regard to sample average trait CVs.

When the 28 basicranial inter-landmark distances are analyzed as a separate module, they indicate that *C. apella s.s.* has an unusually high ICV 95% CI for its mean CV range. *C. apella s.s.’s* mean CV range is markedly lower than this of *C. albifrons,* yet both species have almost identical ICV ranges (Figure 9, Table 14, Figure S2, Table S7). The ICV 95% CIs overlap between all species, and indicate that *C. olivaceus*’ and *C. nigritus*’ ICV values are higher than these of *C. albifrons,* whose ICV range is higher than this of *C. libidinosus* (Table 14, Figure 9, Table S7, Figure S2). These results indicate that the likely diet-driven variation in integration magnitudes within the capuchins is not valid for the basicranium. It is further noticeable that ICVs may vary independently of their associated average trait CVs in the basicranial block of *Cebus nigritus* and *Cebus olivaceus* and in the molar block of several species including *C. olivaceus*, *C. nigritus* and *C. albifrons*, which may be indicative of low within-block correlations.

Furthermore, the slightly higher integration indices associated with the oral block in comparison with the basicranial configuration containing very similar numbers of analyzed inter-landmark distances indicates that segments of the face consistently co-vary more strongly than structures within the basicranium. This finding is consistent with previous cited findings underlining the primacy of the face in cranial integration (e.g., [30]).

**Table 14:** Inter-specific variation in basicranial ICV integration indices.

Species	95% CI ICV	95% CI Mean CV	Actual ICV	Actual mean CV
*C. albifrons*	1.591–1.79	0.054–0.0607	1.63	0.057
*C. olivaceus*	1.678–1.973	0.0505–0.056	1.77	0.053
*C. apella s.s.*	1.606–1.802	0.033–0.0367	1.64	0.035
*C. libidinosus*	1.54–1.70	0.0558–0.0628	1.56	0.059
*C. nigritus*	1.72–2.03	0.0549–0.061	1.82	0.058

### III. Testing the *FMP* Hypotheses and the Cranial Modularity (*CMOD*) Hypotheses: Results from Eigenvalue Variance (EV)

Unlike the situation with the adjusted 95% confidence intervals of the ICVs, the adjusted 95% confidence intervals of the EVs differ to a certain extent from their corresponding unadjusted EV confidence intervals, due to the different nature of the integration index and due to the different relationship between the range of the values within the bootstrap sample and the relative magnitude of the difference between the actual mean and the bootstrap mean from block to block and from species to species.

#### 1. Unadjusted confidence intervals

**Figure 10:**
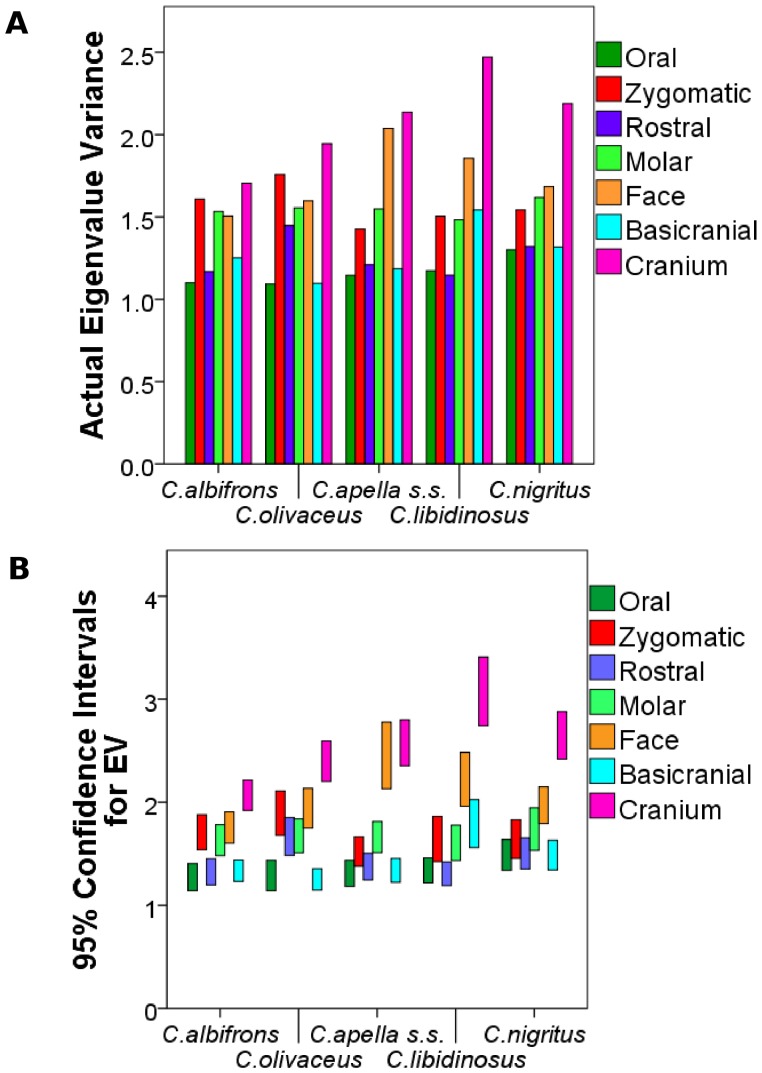
Variation in integration magnitude as measured by EV between species and between modules. A. Distribution of actual EV values; **B.** Distribution of the 95% confidence intervals for EVs.

An interspecific comparison of the eigenvalue variances of the actual samples and their 95% confidence intervals obtained from a sample with 1000 bootstrap replicates indicates that the 95% CI of the facial eigenvalue variance of *C. albifrons* is significantly lower than these of *C. apella s.s.* and *C. libidinosus. C. olivaceus*’ facial EV 95% CI is almost identical to this of *C. nigritus* and overlaps with the 95% EV ranges of all species except for *C. apella s.s.* Both *C. olivaceus* and *C. nigritus* are characterized by significantly lower facial EVs than *C. apella* s.s. (Figure 10). These findings lend support to the H_(FMP1)_ hypothesis.

The integration among the cranial landmarks is lowest in *C. albifrons*, followed by *C. olivaceus*, *C. apella*, *C. nigritus* and *C. libidinosus*. The cranial 95% CIs are non-overlapping between *C. albifrons* and the apelloids, and overlapping between *C. olivaceus* and all species except for *C. libidinosus.* The *FMP1* trend is not observed in the rostral, molar and zygomatic facial subunits or within the basicranium.

A within-species comparison of eigenvalue variances from block to block allows defining the modular architecture of the cranium. The 95% CIs of the cranial EVs are non-overlapping with the facial EV CIs in all species but *C. apella s.s.,* which is characterized by an exceptionally high facial integration. Thus, in all species but *C. apella s.s.*, the cranium as a structure is significantly more integrated than the face. The largest difference in intensity between the facial and the cranial blocks is seen in *C. libidinosus*.

In *C. olivaceus* and *C. albifrons*, the zygomatic block has a very similar integration magnitude to the face (the zygomatic is slightly more integrated than or as integrated as the face under the actual eigenvalue variance comparison and the 95% CIs comparison), while in the apelloids the face is significantly (or marginally significantly in *C. nigritus*) more integrated than the zygomatic. The zygomatic 95% CIs are overlapping between species (except for the zygomatics of *C. olivaceus* and *C. apella s.s.*).

The 95% EV ranges of the molar, zygomatic and facial blocks overlap in all comparison pairs in the gracile species and in *C. nigritus* (although, as noted, the distinction between the facial and the zygomatic EVs in the latter species is marginally significant). The zygomatic and the molar 95% CIs EVs are higher than the oral EVs in all species, and significantly higher than the oral EVs in the gracile species, consistent with the finding that the oral block is composed by more than one module. The rostral-zygomatic 95% CIs for EVs are lower and non-overlapping with the molar 95% CIs in *C. albifrons*, *C. apella s.s.* and *C. libidinosus*. In *C. olivaceus*, the rostral-zygomatic EV 95% CI includes a range of values almost identical to its molar 95% CI. When the EVs from the actual species samples are compared, they indicate that the zygomatic and the molar blocks are more integrated than the rostral and the oral blocks.

The basicranial EV 95% CIs are lower and non-overlapping with those of facial 95% CIs in all species but *C. libidinosus*. Thus, in all species except for *C. libidinosus*, the face is significantly more integrated than the basicranium. The 95% CIs indicate that the basicranium is significantly less integrated than the molar block in *C. albifrons*, *C. olivaceus* and *C. apella s.s.* The 95% CIs for EVs of the basicranium overlap with these of the zygomatic block in the apelloids, while basicranial integration is significantly lower than zygomatic integration in the gracile species.

**Figure 11:**
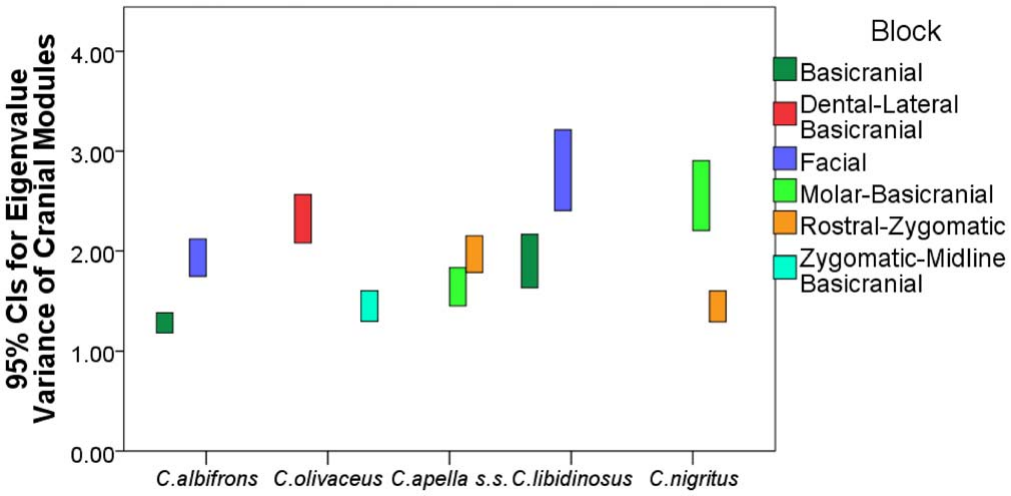
Variation in integration intensity of maximum cranial modularity blocks measured by EVs 95% confidence intervals.

No specific trend is observed when interspecific variation in integration magnitudes within cranial modules maximizing modularity are examined (Figure 11), with the exception that although the cranial landmark partition is approximately identical in *C. albifrons* and *C. libidinosus*, both the facial and the basicranial modules of *C. albifrons* are significantly less integrated.

#### 2. Adjusted confidence intervals

The adjusted EV values produce more important overlap between the 95% confidence intervals of different cranial blocks, but further demonstrate that the face is not more integrated than the cranium, that facial integration is particularly high and not significantly different from cranial integration in *C. apella s.s.*, that all facial modules in *C. apella s.s.* are significantly less integrated than the face while in the rest of the capuchins the bootstrap distribution of the facial and molar blocks are almost identical (except for *C. libidinosus* in which the overlap is limited) (Figures S3).

The adjusted 95% confidence intervals for the EV values further suggest that the oral and the rostral blocks are significantly less integrated than the face and the cranium in all species (except for the rostral block of *C. olivaceus*), that the molar block 95% CIs are markedly higher and non-overlapping or marginally overlapping with the 95% CIs of the oral and the rostral blocks in all species except for *C. olivaceus*, that all purported facial sub-modules are significantly less integrated than the cranium in all species except for the zygomatic and the molar blocks in the untufted species and that the basicranium is significantly less integrated than the face and the cranium in all species except for *C. libidinosus*, in which the basicranial and the facial 95% confidence intervals overlap. It is further suggested that the basicranial 95% confidence interval generally extensively overlaps with the 95% confidence intervals of the oral and the rostral blocks (with the exception of the high rostral integration in *C. olivaceus* producing non-overlapping rostral and basicranial confidence intervals, and the high basicranial integration in *C.libidinosus* producing a basicranial confidence interval that does not overlap with the rostral and oral intervals). Finally, an examination of the adjusted EVs of the maximum cranial modularity blocks further confirms that the facial and the basicranial blocks of *C. albifrons* are characterized by significantly lower integration magnitudes than the corresponding blocks of *C. libidinosus* (Figure S4).

### IV. Testing the Cranial Modularity (*CMOD*) Hypotheses: Results from Cluster Analysis Using Ward’s Method

**Figure 12:**
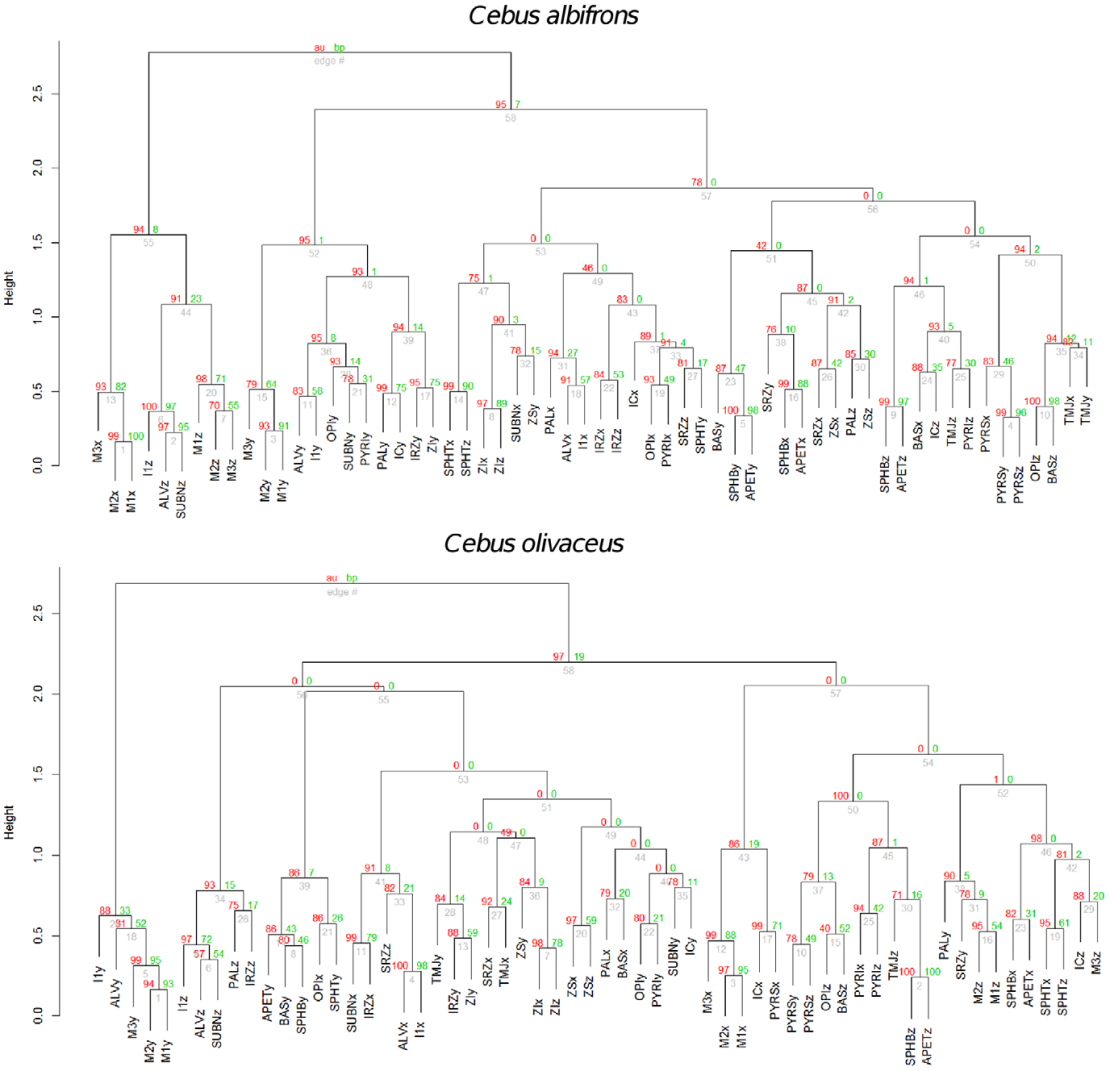
Association between cranial landmark coordinates as assessed by cluster analysis using Ward’s method of linkage. Coordinate clusters in the gracile capuchins. Legend: x signifies medio-lateral displacement of the coordinate, y signifies supero-inferior displacement of the coordinate, z signifies antero-posterior displacement of the coordinate.

**Figure 13:**
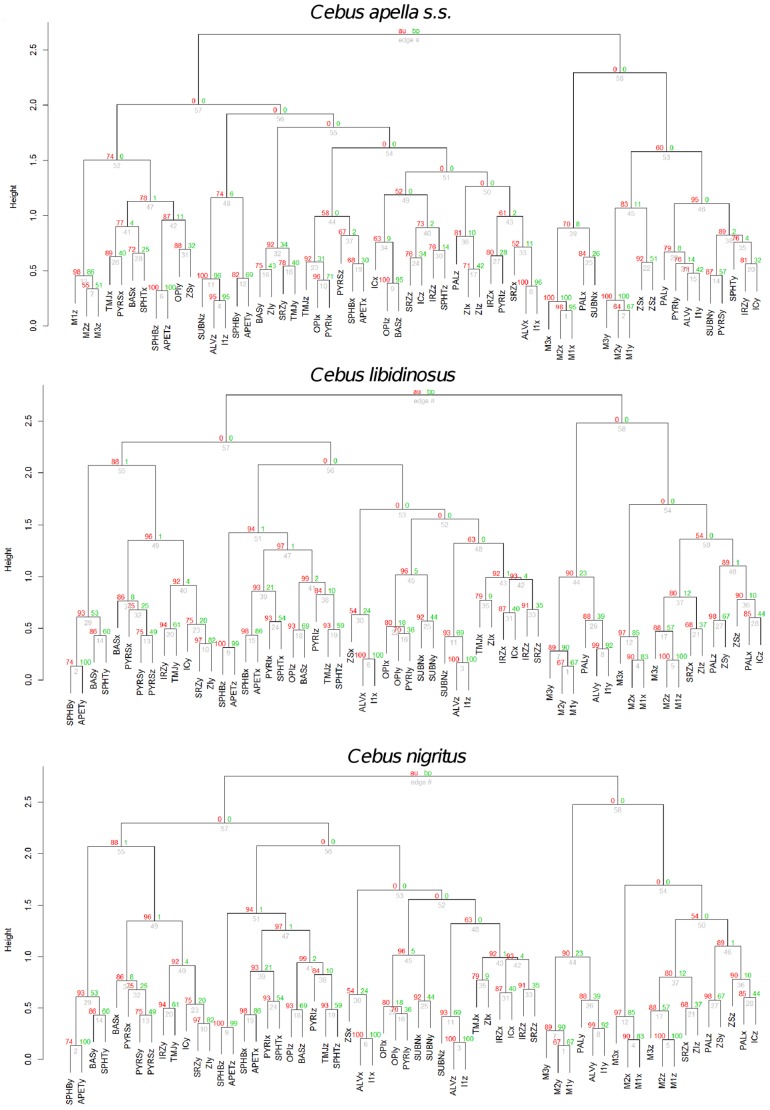
Association between cranial landmark coordinates as assessed by cluster analysis using Ward’s method of linkage. Coordinate clusters in the apelloid capuchins. Legend: x signifies medio-lateral displacement of the coordinate, y signifies supero-inferior displacement of the coordinate, z signifies antero-posterior displacement of the coordinate.

There is no evidence supporting the segregation of facial and basicranial landmarks into two statistically significant clusters in any *Cebus* species, when the Ward’s method of linkage is applied to distance matrices obtained from correlation matrices of the pooled within subspecies and sex residuals regressed on centroid size. Facial coordinates variably co-vary with basicranial coordinates. The majority of the basicranial landmarks cluster in a distinct module along with some facial coordinates, but vary independently from other facial coordinates in *Cebus olivaceus*, *Cebus albifrons*, *Cebus libidinosus* and *Cebus apella s.s*. (Figure 12, 13). In *Cebus albifrons*, the existence of the larger of the two most inclusive clusters is supported by statistical tests and includes all basicranial landmarks and the majority of the facial landmarks, while the other cluster is nearly significant and includes the x and z coordinates of all molar landmarks in addition to the z coordinates of nasospinale, alveolare and the lingual incisor alveolar landmark. Within the larger cluster, a smaller cluster including the y coordinates of the molar landmarks, opisthion, the inferior petrous pyramid, several rostral landmarks and the palatal landmark is also significant. There are no other large and significant cranial modules.

In *Cebus olivaceus*, the larger of the two clusters produced by the basic subdivision of the variables includes all basicranial landmarks and most facial landmarks and is statistically significant, but it does not include any reasonably large significant submodules with the exception of a block combining nine variables including the z coordinates of all basicranial landmarks except for the inferior sphenotemporal landmark in addition to some x and y basicranial coordinates, and a second block combining other basicranial coordinates and two dental coordinates. In *Cebus apella s.s.*, no large module is significant. The largest significant coordinate subdivision includes nine y coordinates of the following landmarks: inferior petrous pyramid, superior petrous pyramid, palate, alveolare, lingual incisor, nasospinale, sphenotemporal inferior, inferior zygomatic root and incisor/canine landmark. The next three largest significant modules include three landmarks: the z coordinates of the molar landmarks, the y coordinates of the molar landmarks and the x coordinates of the molar landmarks, respectively. As in *Cebus apella s.s.*, in *Cebus libidinosus*, no large module is significant either. The largest significant module includes nine basicranial coordinates, these of the inferior petrous pyramid, basion, opisthion, the pyramidal apex, sphenotemporal inferior, and sphenobasion. It is interesting to note that within this cluster, the x coordinates along with one z coordinate cluster in a smaller module significantly different from the block combining the y coordinates. The next largest block includes seven coordinates representative of zygomaxillare superior, the superior zygomatic root, basion, opisthion and the palatal landmark.

As in the other apelloids, in *Cebus nigritus*, no larger block is significant either. The largest significant cluster includes nine mostly y coordinates of the incisor/canine landmark, the superior zygomatic root, zygomaxillare inferior, the TMJ, the inferior zygomatic root, the superior and inferior petrous pyramid and basion. A nearly significant module including eleven variables unites exclusively the coordinates of all but seven of the rest of the basicranial coordinates.

Thus, in no capuchin species does the composition of the significant clusters represent compelling evidence for the existence of facial modules (i.e., molar, zygomatic, rostral). Furthermore, within the cluster containing most basicranial coordinates, these basicranial coordinates do not form modules to the exclusion of the facial coordinates, which again indicates that the face and the basicranium are not strictly modules.

### V. Testing the Heterochrony (*HET*) Hypotheses: Integration Magnitudes in Males and Females as Measured by RV Coefficients

**Figure 14:**
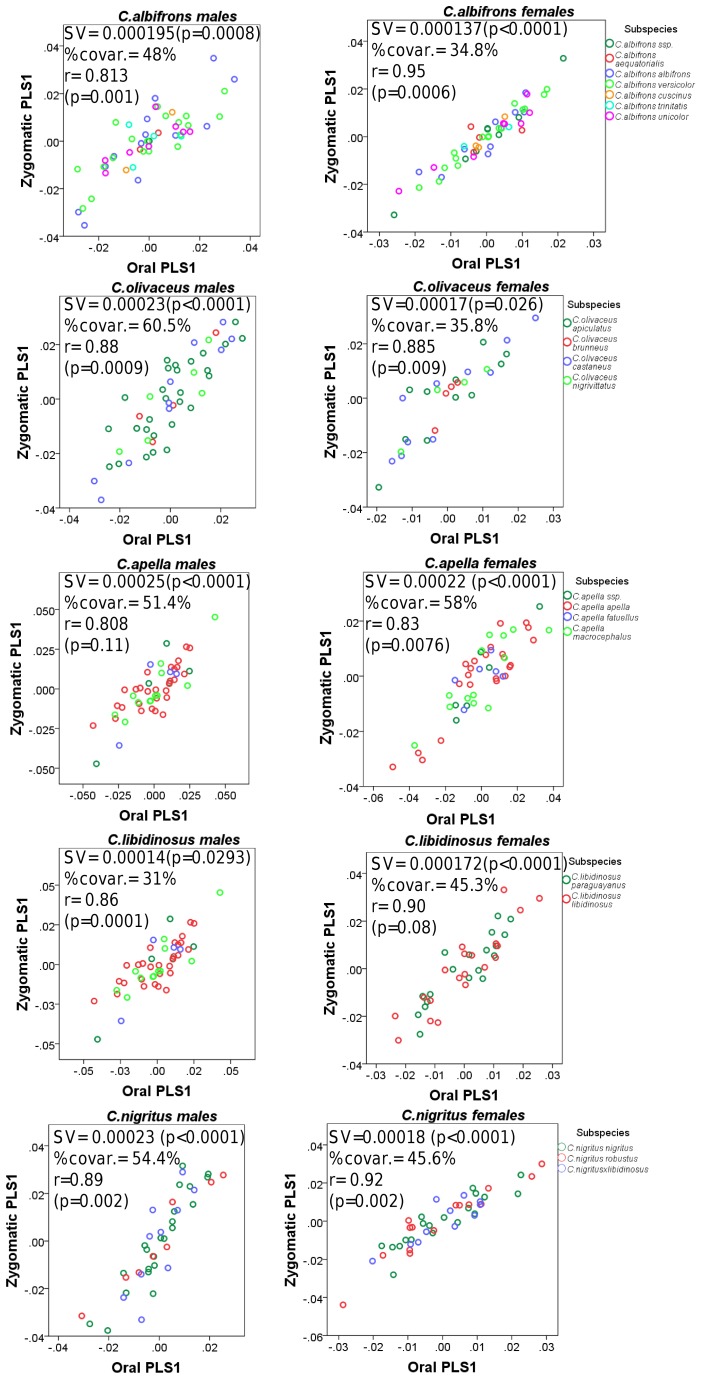
Differences in between-block association between males and females. Distribution of the individuals’ loadings along the PLS1 axis of each block under an *allometric* oral-zygomatic PLS. Abbreviations: SV = singular value, %covar. = percentage of between-block total squared covariance explained by the set of PLS1 axes, r = between-block correlation coefficient.

**Figure 15:**
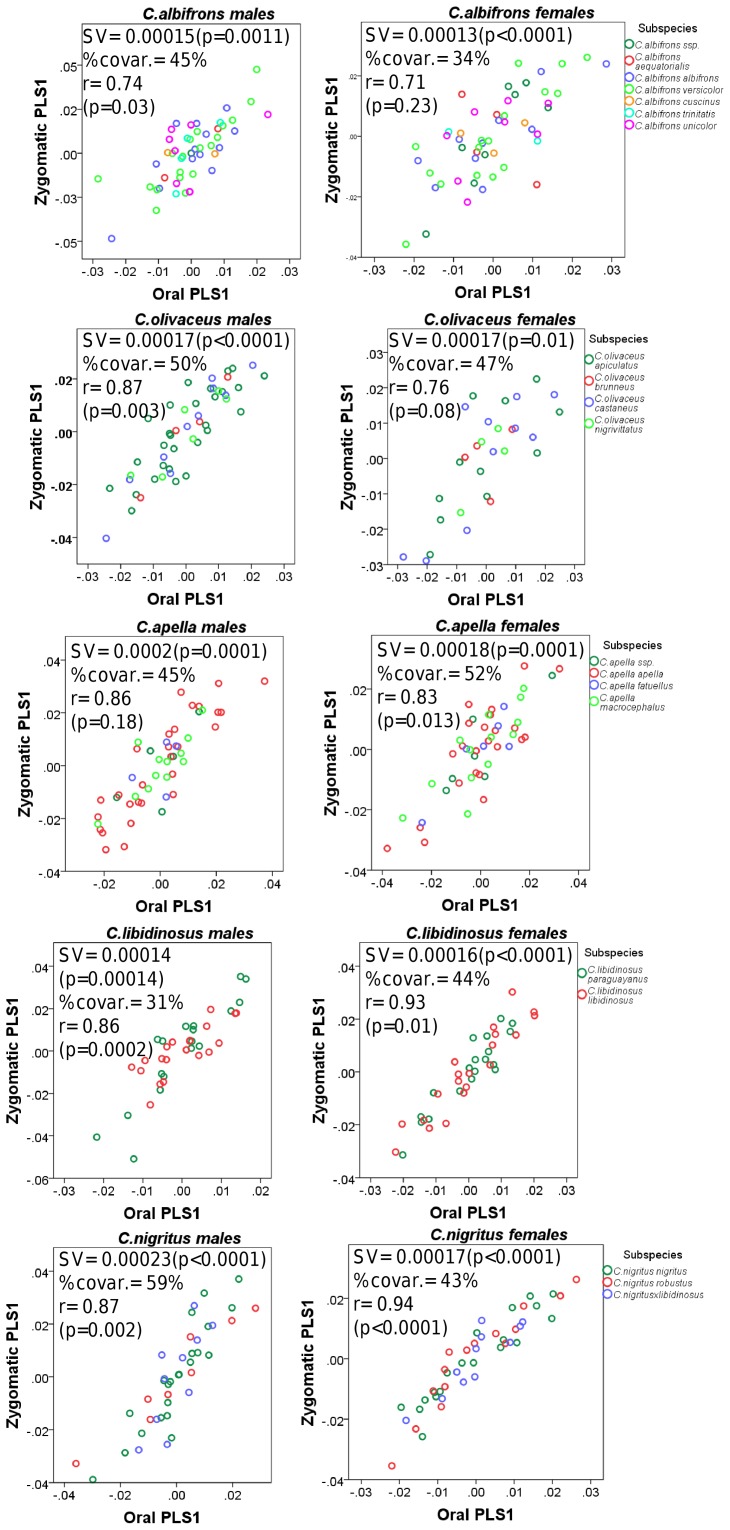
Differences in between-block association between males and females. Distribution of the individuals’ loadings along the PLS1 axis of each block under an *non-allometric* oral-zygomatic PLS. Abbreviations : SV = singular value, %covar. = percentage of between-block total squared covariance explained by the set of PLS1 axes, r = between-block correlation coefficient.

The *HET* alternative hypotheses are not supported when oral-zygomatic RV coefficients (containing or not allometric variation), percentages of squared between-block covariance explained by PLS1 or PLS1 between-block correlation coefficients are considered (Table 15, Figure 14, 15).

**Table 15:** Distribution of integration magnitudes under an allometric and a non-allometric PLS, as measured by the RV coefficient.

Species/sex	Allometric Face(oral-zygomatic)	p-value	Non-allometric Face(oral-zygomatic)	p-value
*C. albifrons ♂*	0.40	<0.0001	0.39	<0.0001
*C. albifrons ♀*	0.45	<0.0001	0.43	<0.0001
*C. olivaceus ♂*	0.41	= 0.0001	0.37	= 0.0001
*C. olivaceus ♀*	0.434	= 0.002	0.40	= 0.0045
*C. apella s.s. ♂*	0.505	<0.0001	0.50	<0.0001
*C. apella s.s. ♀*	0.41	<0.0001	0.40	<0.0001
*C. libidinosus ♂*	0.426	<0.0001	0.43	<0.0001
*C. libidinosus ♀*	0.526	<0.0001	0.52	<0.0001
*C. nigritus♂*	0.47	<0.0001	0.47	<0.0001
*C. nigritus♀*	0.45	<0.0001	0.46	<0.0001

### VI. Testing the Heterochrony (*HET*) Hypotheses: Integration Magnitudes in Males and Females as Measured by ICVs

**Figure 16:**
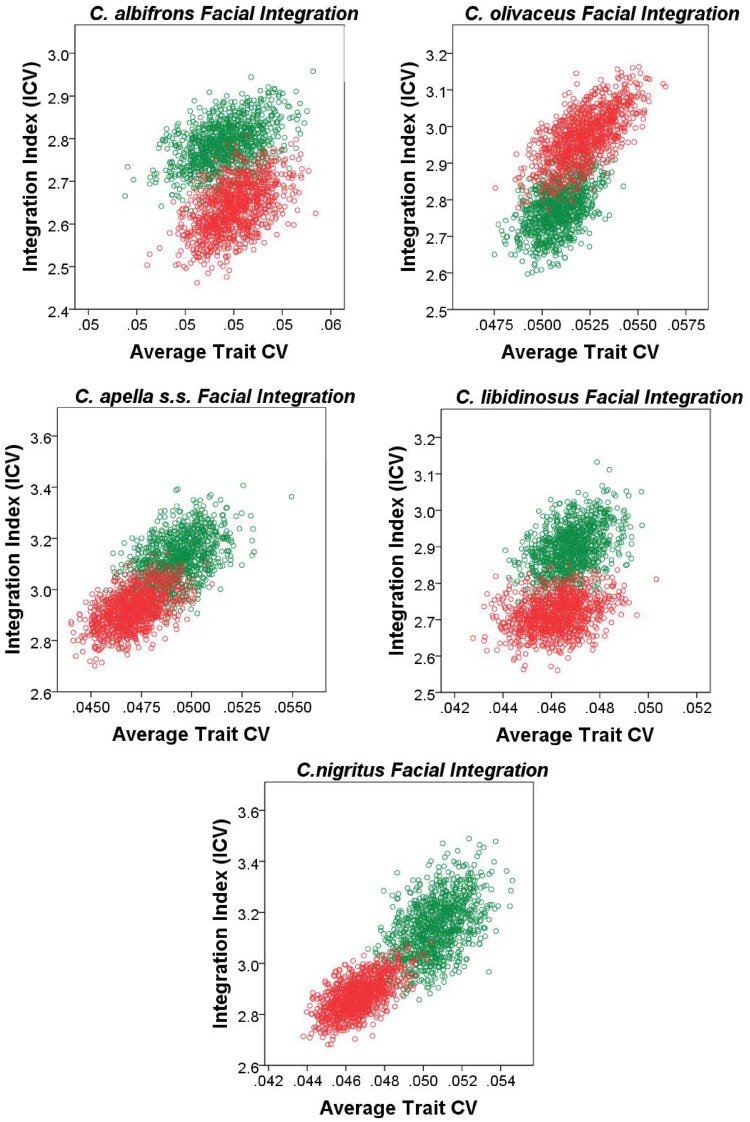
Variation in facial integration (ICVs) between males and females with regard to average trait CVs. Legend: Males in green, females in red.

**Figure 17:**
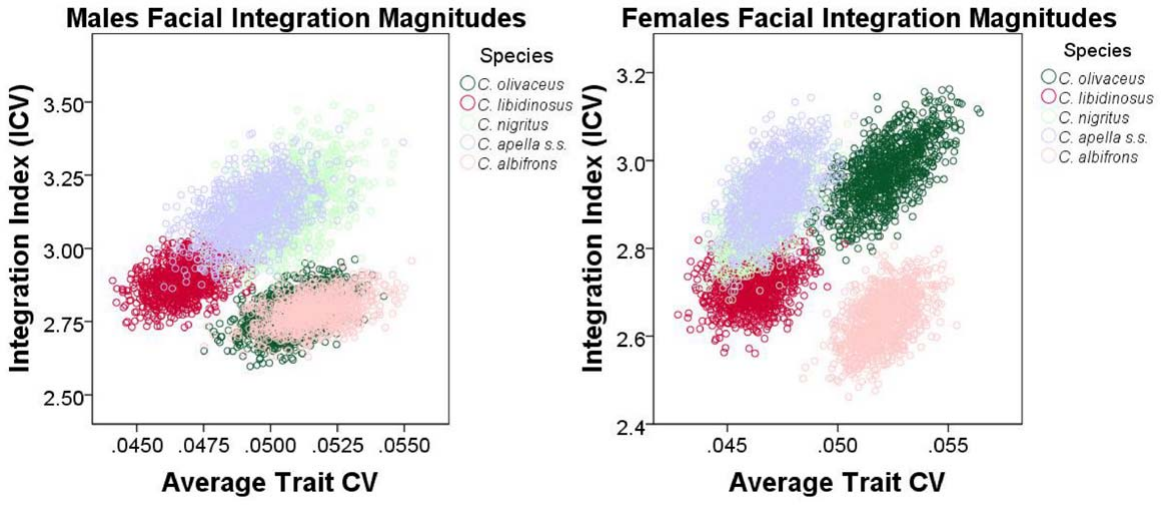
Interspecific variation in facial ICVs within males and females with regard to average trait CVs.

The actual ICV values indicate that in all species but *C. olivaceus* males have larger ICV values than females and that all apelloids have lower mean CV values than the gracile species (Table 16). Yet, a comparison of the integration indices at a common level of sampled mean CV of 0.049 derived from both adjusted and unadjusted distributions does not lend support to any of the *HET* alternative hypotheses (Table 16, Table S8). The ICV ranges overlap between males and females in all species except for *C. libidinosus* and *C. albifrons*, in which males have significantly higher ICVs (Figure 16, Figure S5). In *C. olivaceus*, the female ICV range is higher and almost non-overlapping with this of its corresponding male ICV range. When the 95% distributions of facial ICVs with regard to mean trait CVs are examined within each sex inter-specifically, it is shown that males *C. albifrons* and *C. olivaceus* and females *C. albifrons* are characterized by clearly lower ICVs and higher mean trait CVs than the other species in each sex category (Figure 17, Figure S6).

**Table 16:** Variation in facial ICV integration indices between the sexes of the different species.

Species	Actual ICV	Actual mean CV	ICV at a mean CV of 0.049
*C. albifrons ♂*	2.63	0.0514	2.67–2.80
*C. albifrons ♀*	2.48	0.0517	2.55 at CV of 0.0497
*C. olivaceus ♂*	2.62	0.0508	2.63–2.84
*C. olivaceus ♀*	2.79	0.0508	2.82–2.92
*C. apella s.s. ♂*	2.928	0.042	2.91–3.32
*C. apella s.s. ♀*	2.745	0.0468	2.87–3.1
*C. libidinosus ♂*	2.731	0.0491	2.91–3.0
*C. libidinosus ♀*	2.572	0.046	2.75
*C. nigritus ♂*	2.92	0.0504	2.87–3.28
*C. nigritus ♀*	2.704	0.0465	2.9–3.3

### VII. Testing the Heterochrony (*HET*) Hypotheses: Integration Magnitudes in Males and Females as Measured by EVs

**Figure 18:**
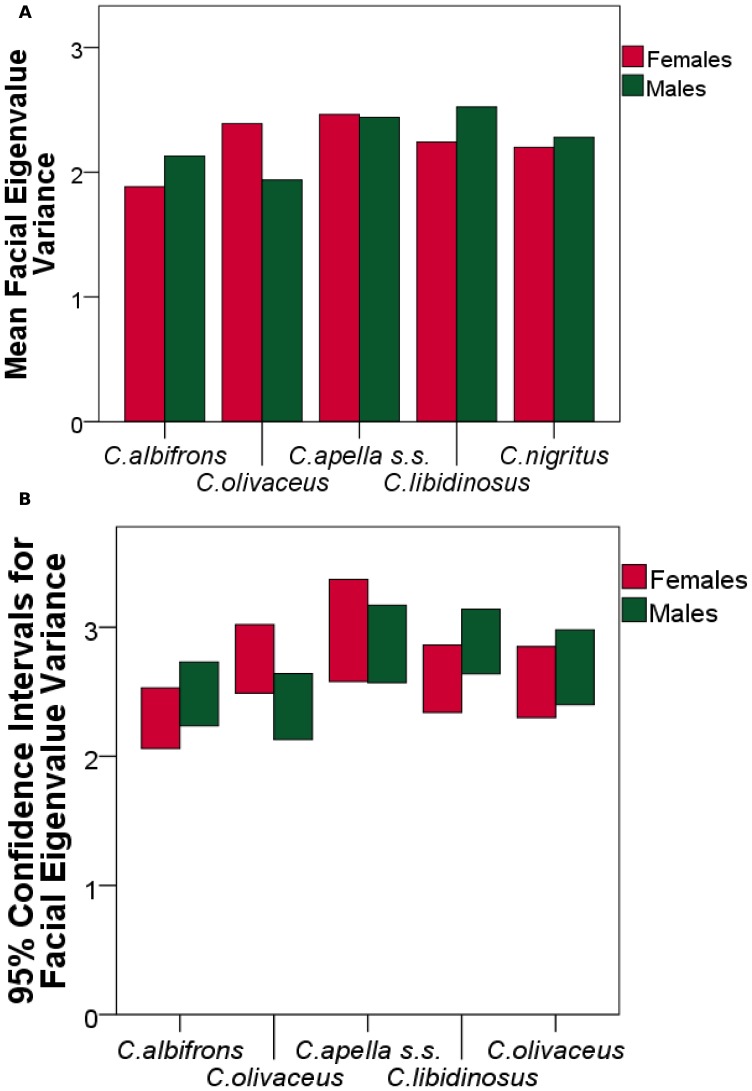
Variation in integration magnitude as measured by EV between males and females. Legend: Males in green, females in red. A. Distribution of actual EV values; B. Distribution of the 95% confidence intervals for EVs.

The distribution of the actual EV values and of their 95% confidence intervals between males and females within each species does not support the idea that sexual dimorphism influences integration magnitudes and does not produce any statistically significant differences in facial integration magnitudes between the males and females within each species (Figure 18).

The adjusted 95% confidence intervals of the EVs do not modify these conclusions (Figure S7).

### VIII. Similarity in Facial Integration Patterns

#### 1. Allometric PLS1 shape change

In all species, the distribution of the specimens’ scores in the PLS1(oral) - PLS1(zygomatic) shape space after controlling for the effect of subspecies, defines a male morph and a female morph. The males-to-females transformation follows an identical pattern in *C. apella s.s.*, *C. nigritus*, *C. libidinosus* and *C. albifrons*.

**Figure 19:**
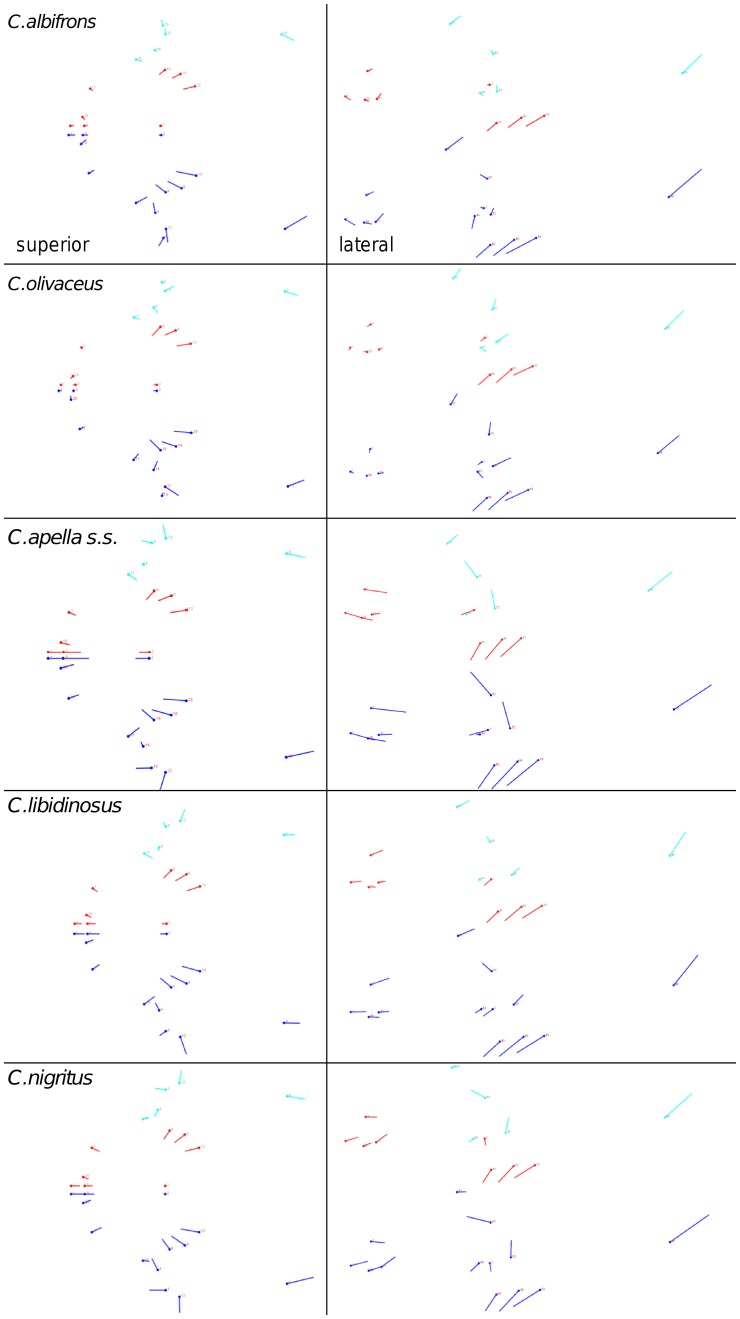
Shape change contained in the allometric oral-zygomatic PLS1 and the allometric PC1. Legend: oral-zygomatic PLS1 blocks colored in red and turquoise; PC1: landmark configuration colored in dark blue.

In comparison with the average species pattern, the characteristics of the male pattern in the allometrically driven trait relationships include a narrower dental arcade at the level of the molars, more anteriorly shifted molars relative to the TMJs, relatively smaller cheek teeth and more posteriorly located incisor arc (Figure 19). Other characteristics defining this pattern include more laterally flaring zygomatic roots, a taller lower face, a slightly taller midface due to a more superior location of the infraorbital margin, and a higher position of the TMJs. The shape of the males’ zygomatic roots also places the zygomaxillare inferior in a more lateral position.

In all tufted capuchin species, the males’ and females’ configurations along the allometry-containing PLS1 axes are distinct with little overlap between the sexes, unlike the situation in *C. albifrons* and *C. olivaceus* (Figure 4). This characteristic likely stems from the observed little overlap in centroid sizes between apelloid males and females, indicative of an important degree of facial dimorphism.

Each species’ extreme configurations define ranges in centroid sizes indicating that cranial size variation cannot account for the higher RV coefficients in the tufted species (centroid size ranges: *C. apella s.s.*: 95–127 units (range = 32), *C. libidinous*: 95–118 units (range = 23), *C. nigritus*: 95–119 (range = 24), *C. olivaceus*: 95–121 units (range = 26), *C. albifrons*: 87–114 units (range = 27). Thus, centroid size ranges are not wider in the apelloids compared to the gracile capuchins despite the more extensive overlap in cranial size between gracile males and females. In the same time, the crania of *C. albifrons* are on average smaller than the apelloid and the *C. olivaceus* crania, which indicates that the starting point for the allometric transformation in *C. albifrons* is different from this of the tufted species and *C. olivaceus*.

Despite the similarity in transformation patterns between *C. albifrons* and *C. olivaceus* and the apelloids, the morphology of the male and female configurations differs. Males belonging to the gracile species are characterized by more anteriorly projected rostrums relative to the average pattern in comparison with their apelloid counterparts (Figure 19). Consequently, rostral projection is more sexually dimorphic in the tufted capuchins.

The *C. olivaceus* PLS1 shape change pattern is very similar to this of the tufted species and *C. albifrons*. Contrarily to the other species, in *C. olivaceus* the PLS1 does not involve notable variation in the shape or position of the incisor arc, combined with posteriorly migrating TMJs. This observation is consistent with the high ICV and EV values of the rostral block of *C. olivaceus*, indicating little covariation with other units. The marked allometric covariation between the rostrum and the TMJ in the apelloids ensuring that both landmarks are concurrently displaced either anteriorly or posteriorly might be adaptive.

Compared to apelloid males and similarly to *C. albifrons* males, the zygomatic roots of *C*. *olivaceus* males are less laterally flaring, as indicated by the position of the zygomaxillare inferior, which creates a face, narrower than this of similarly-sized *C. albifrons* individuals.

**Figure 20:**
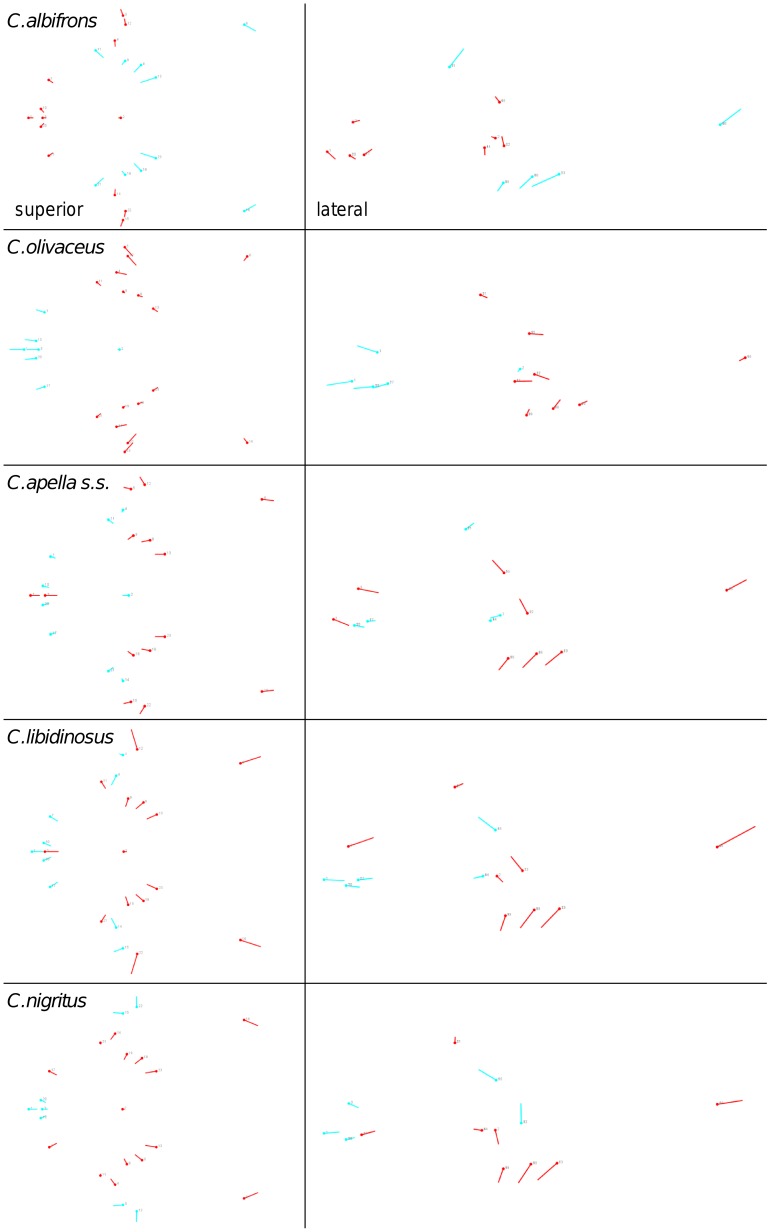
Shape change contained in the allometric maximum facial modularity PLS1.

Shape change contained in the allometric PLS1 under an oral-zygomatic subdivision and under a maximum modularity subdivision is very similar in each species (Figure 20). Differences between the two modularity scenarios are expressed in the magnitudes of the vectors at landmarks. In particular, shape change at the molar landmarks and the TMJs are the principal driver of covariation under the oral-zygomatic modularity hypothesis, while the maximum modularity scenario grouping the molar landmarks and the TMJs in a single block allows for the expression of rostral and zygomatic shape change.

**Figure 21:**
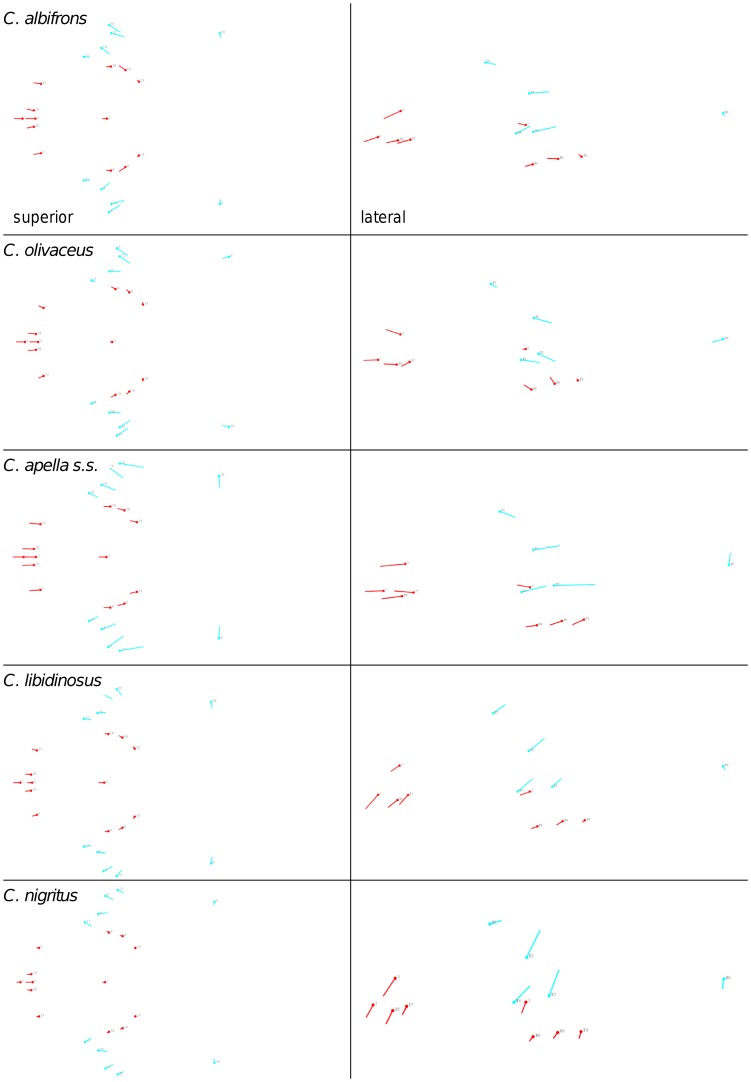
Shape change contained in the allometric oral-zygomatic PLS2.

Slight distinctions in the orientation and the magnitude of the vectors at landmarks between the oral-zygomatic integration hypothesis and the maximum modularity scenario are noticeable despite the general similarity in pattern. In *C. albifrons*, *C. apella*, *C. libidinosus* and *C. nigritus*, the similarity between shape change under the oral-zygomatic hypothesis and the maximum modularity scenario is almost full. In *C. olivaceus,* the maximum modularity PLS2 contains a shape change more similar to the PLS1 of the oral-zygomatic patterning, and, as previously noted, also contains a higher proportion of allometric change (Figure 21). The *C. olivaceus* PLS2 pattern includes a molar segment of the larger individuals (there is a complete overlap between males and females) that is anteriorly shifted and slightly narrower; however, unlike the situation in the PLS1 of the other capuchins, the PLS2 extreme morph of the larger individuals does not have a taller lower face (molar alveoli not too inferiorly located), the infraorbital margins do not change their supero-inferior position, the superior zygomatic root and the zygomatic arch migrate inferiorly rather than superiorly, there is no variation in the position of the zygomaxillare inferior, and the TMJs are more posterior but not more medial. Non-allometric shape change associated with the maximum modularity PLS1 of *C. olivaceus* (under the allometric scenario) includes a posterior shift of the rostrum and more laterally flaring, slightly more anterior zygomatics, and slightly taller lower face and mid face.

#### 2. Non-allometric PLS1 shape change: comparison with the allometric PLS1

**Figure 22:**
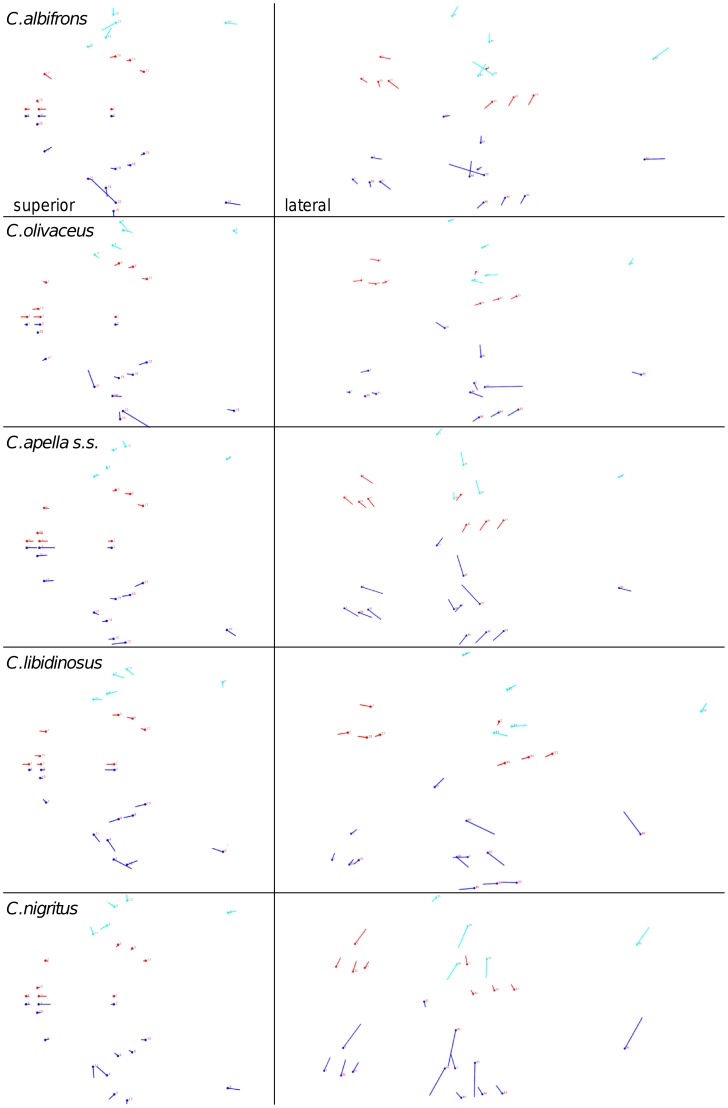
Shape change contained in the non-allometric oral-zygomatic PLS1 and the allometric PC1. Legend: oral-zygomatic PLS1 blocks colored in red and turquoise; PC1: landmark configuration colored in dark blue.

There is a globally good correspondence between the allometric PLS1 and the non-allometric PLS1 of *C. apella s.s.* and *C. albifrons*, the allometric PLS1 and the non-allometric PLS2 of *C. libidinosus* and the allometric PLS1 and the non-allometric PLS3 of *C. nigritus*. In these species, the similarities between non-allometric patterns and the allometric PLS1 pattern are extensive and consist of the typical shortening of the jaws via a posterior displacement of the rostral landmarks and an antero-inferior and to an extent medial displacement of the molar landmarks, a superior displacement of the zygomatic landmarks, a posterior displacement of the infraorbital margin as measured by zygomaxillare superior (except for *C. nigritus*), and a superior displacement of the TMJs accompanied by a concomitant posterior shift in TMJs (except for *C. libidinosus*), a taller lower face (except for *C. libidinosus*) and a slight lateral displacement of the zygomatic landmarks (Figure 22).

In the four capuchins (except for *C. olivaceus*), in comparison to the allometric PLS1 pattern, the non-allometric PLS shape change consists of less medio-lateral variation in the molar landmarks (with the exception of M1 in *C. libidinosus*), markedly less or no antero-posterior variation in the TMJ position, and a narrower molar arcade not associated with markedly medially displaced TMJs.

In the non-allometric oral-zygomatic PLS1 of *C. nigritus*, one of the extreme patterns is mostly characterized by a shorter lower face, by TMJs more superior to the occlusal plane and more posterior relative to the molars.

**Figure 23:**
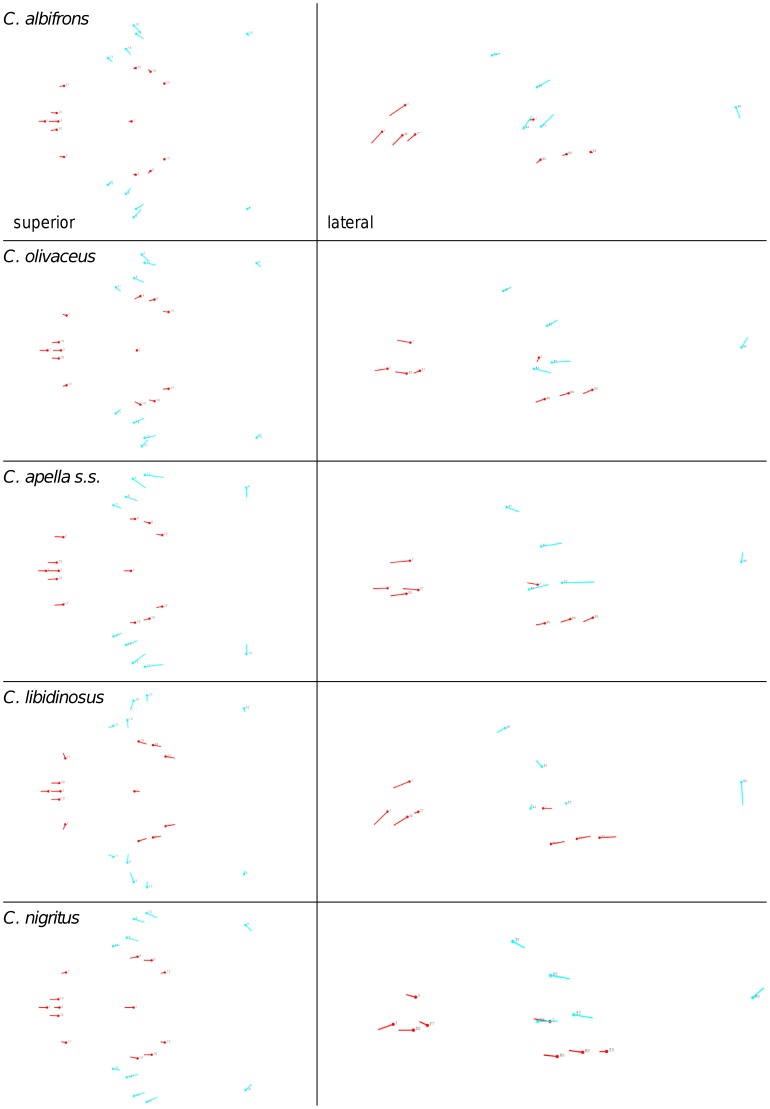
Shape change contained in the non-allometric oral-zygomatic PLS2.

Non-allometric oral-zygomatic shape change contained in the PLS1 of *C. olivaceus* and *C. libidinosus* corresponds to shape change contained in the PLS2 of *C. apella* and *C. nigritus*. One of the extreme configurations combines an anterior displacement of the dental landmarks and nasospinale with a shorter lower face, a posterior and slightly medial displacement of the zygomatic landmarks and a supero-medial displacement of the TMJs (Figure 23).

**Figure 24:**
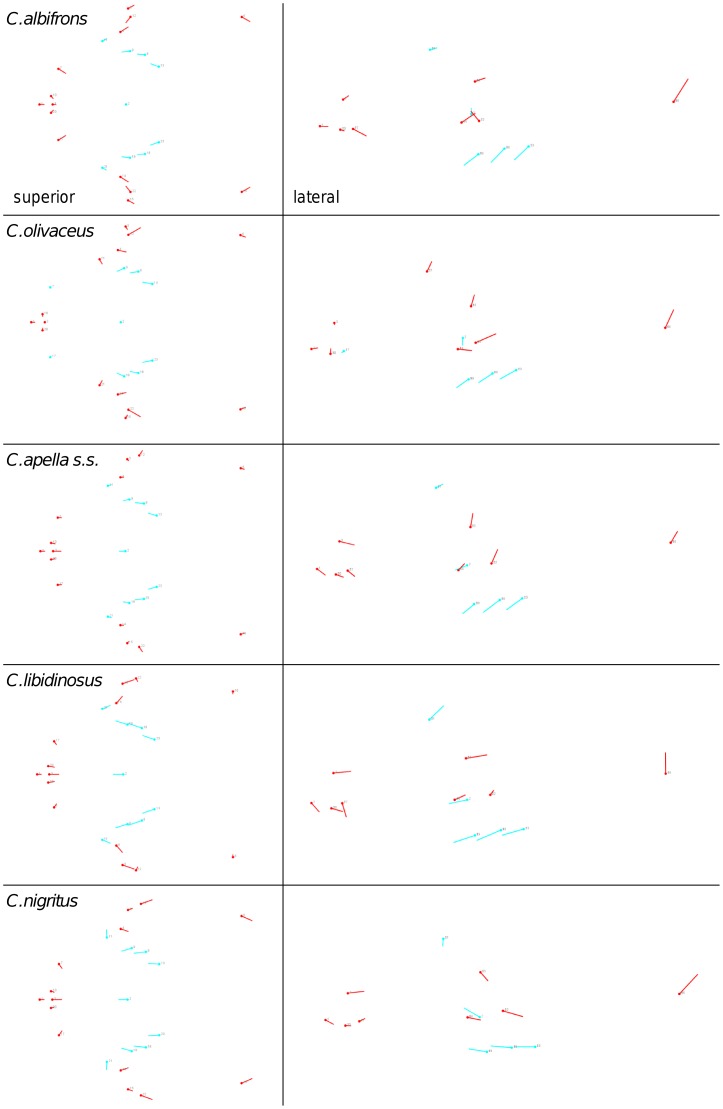
Shape change contained in the non-allometric maximum modularity PLS1.

The maximum modularity non-allometric PLS1 contains a pattern reminiscent of both the maximum modularity allometric PLS1 and the oral-zygomatic non-allometric PLS1. In all species, the maximum modularity non-allometric pattern differs from the oral-zygomatic non-allometric pattern by a more exaggerated anterior displacement of the molars, more posterior zygomatic roots (with the exception of *C. olivaceus*), higher position of the zygomatic roots, and higher TMJs (Figure 24).

One of the important conclusions to be drawn from these comparisons is that the displacements at the TMJs in the supero-inferior and antero-posterior directions, and thus gape, is governed by allometry. Furthermore, the non-allometric axes contain magnified shape change of the rostral and zygomatic landmarks, which is normally obscured by allometry at the expense of molar-TMJ variation. Overall, the absolute magnitude of shape change is higher along the allometric axis, in other terms there are individuals exhibiting more extreme degrees of the pattern.

#### 3. Comparison between PLS 1 and PC 1

Under an allometric scenario shape variation contained in the PC1 axes is almost indistinguishable from variation contained in the PLS1 axis (Figure 19).

Shape change contained in the non-allometric PC1 axis of *C. apella*, *C. nigritus* and *C. albifrons* is very similar to the pattern contained in the non-allometric PLS1 (Figure 22). The *C. libidinosus* PC2 shares extensive similarity with PLS1, and explains slightly lower percentage of the total variance contained in the landmark coordinates than PC1 (PC2∶ 13.13%).

In *C. olivaceus*, shape change contained in the non-allometric PLS1 is also fairly similar to the non-allometric PC2 shape change, which explains 12.43% of the total variance.

#### 4. PLS2: allometric and non-allometric oral-zygomatic integration

Under both the allometric and the non-allometric scenarios, shape change along PLS2 is of very low magnitude (Figure 21, Figure 23). In both the oral-zygomatic allometric PLS2 and the non-allometric PLS2, one of the extreme configurations of all species except for *C. libidinosus* includes a lengthening of the rostrum, a posterior migration of the zygomatic landmarks and the infraorbital margin and a medial migration of the TMJs. The allometric PLS2 of *C. libidinosus* contains similar shape change, while in its non-allometric PLS2 the displacement at the zygomatic landmarks is medial rather than posterior.

#### 5. Shape changes within sex-specific samples

**Figure 25:**
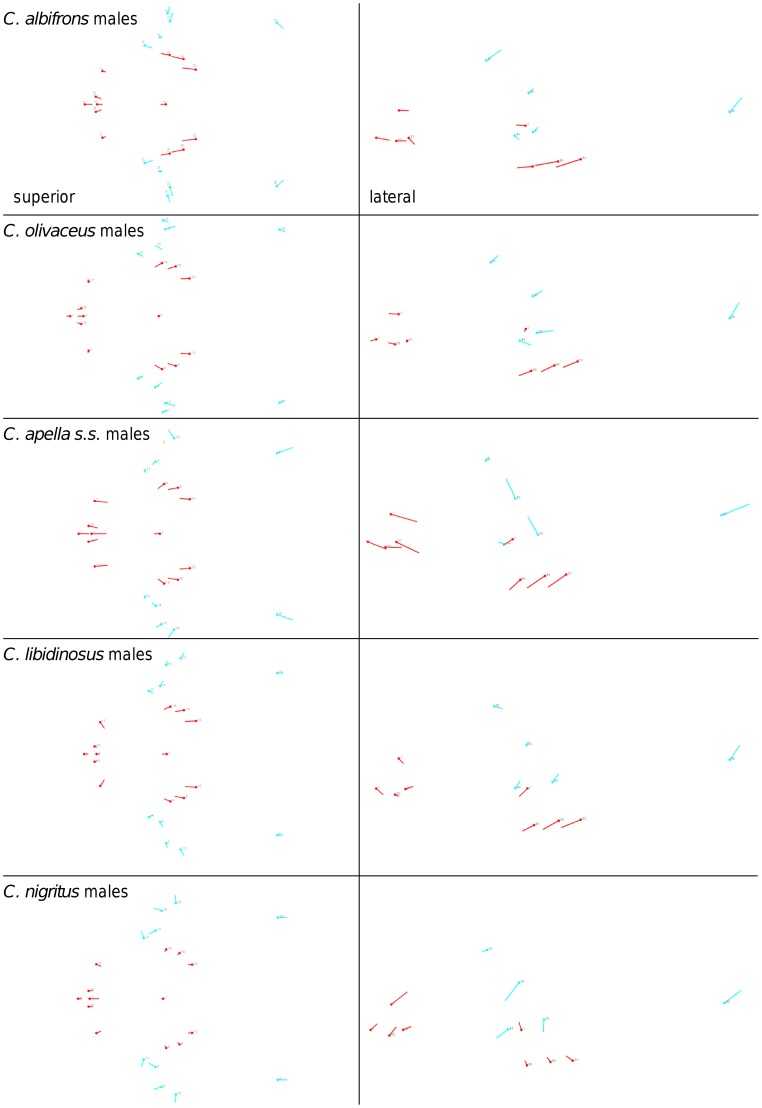
Shape change contained in the allometric oral-zygomatic PLS1 of males.

**Figure 26:**
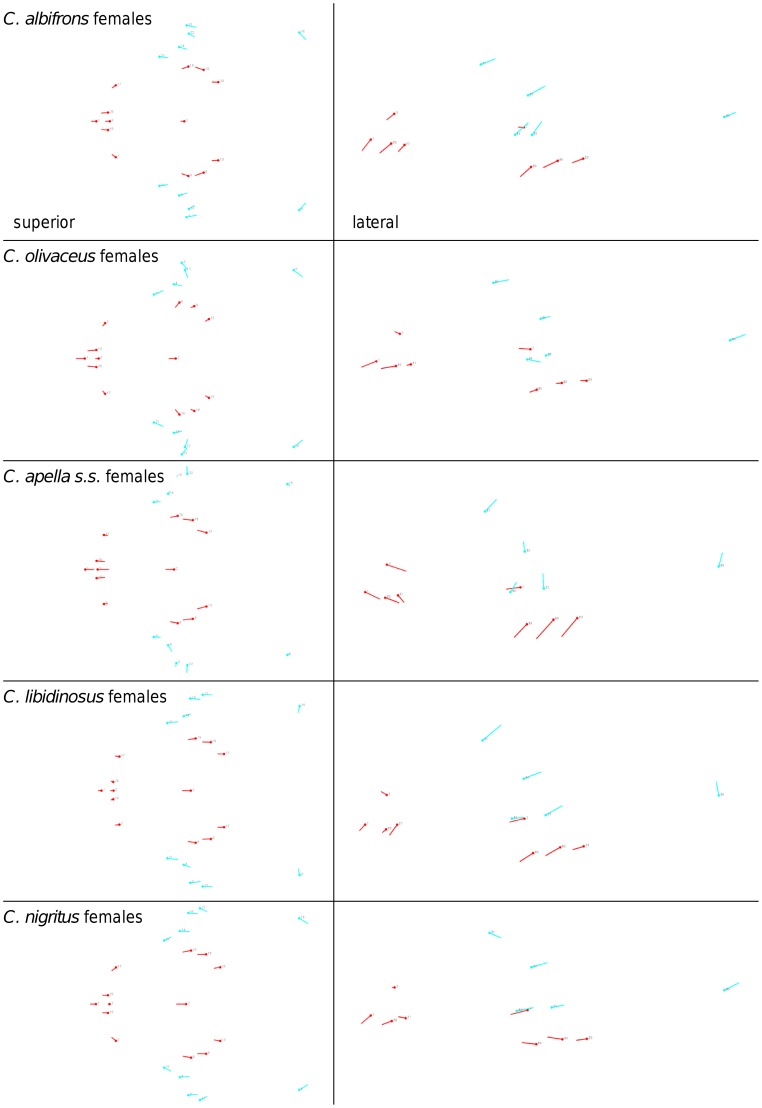
Shape change contained in the allometric oral-zygomatic PLS1 of females.

**Figure 27:**
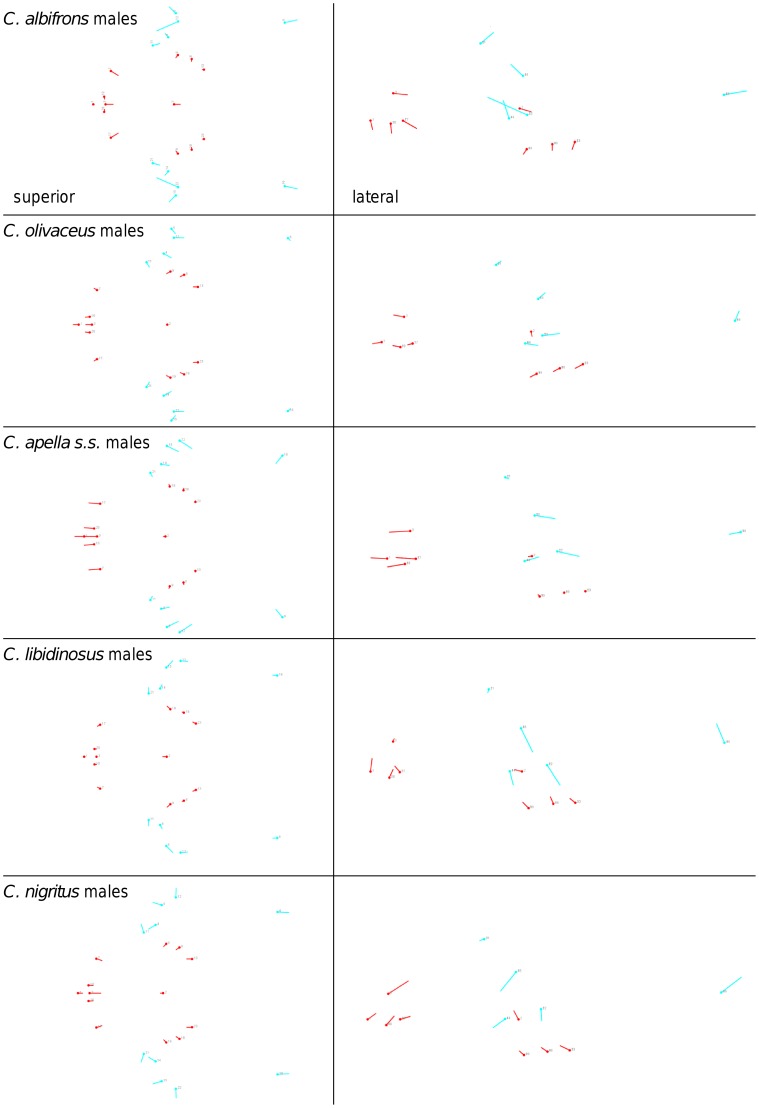
Shape change contained in the non-allometric oral-zygomatic PLS1 of males.

**Figure 28:**
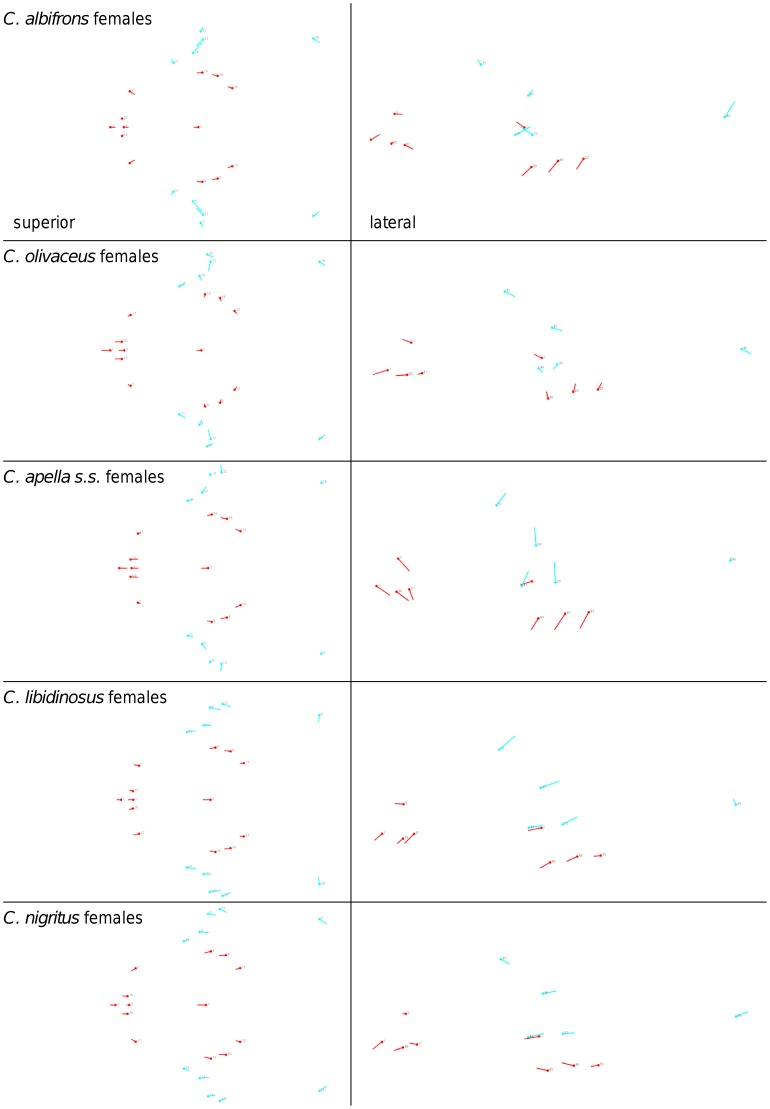
Shape change contained in the non-allometric oral-zygomatic PLS1 of females.

As expected, shape change contained in the allometric PLS1 of several samples, such as *C. albifrons* males, *C. apella* males, *C. apella* females, *C. libidinosus* males and *C. olivaceus* males, is almost identical or fairly similar to this contained in the common allometric PLS1 (Figure 25, Figure 26). Similarity between the non-allometric PLS1 of males and the non-allometric PLS1 of females cannot be established (Figure 27, Figure 28).

### IX. Similarity in Cranial Integration Patterns

#### 1. Shape change contained in the allometric PLS1 and its biomechanical consequences

**Figure 29:**
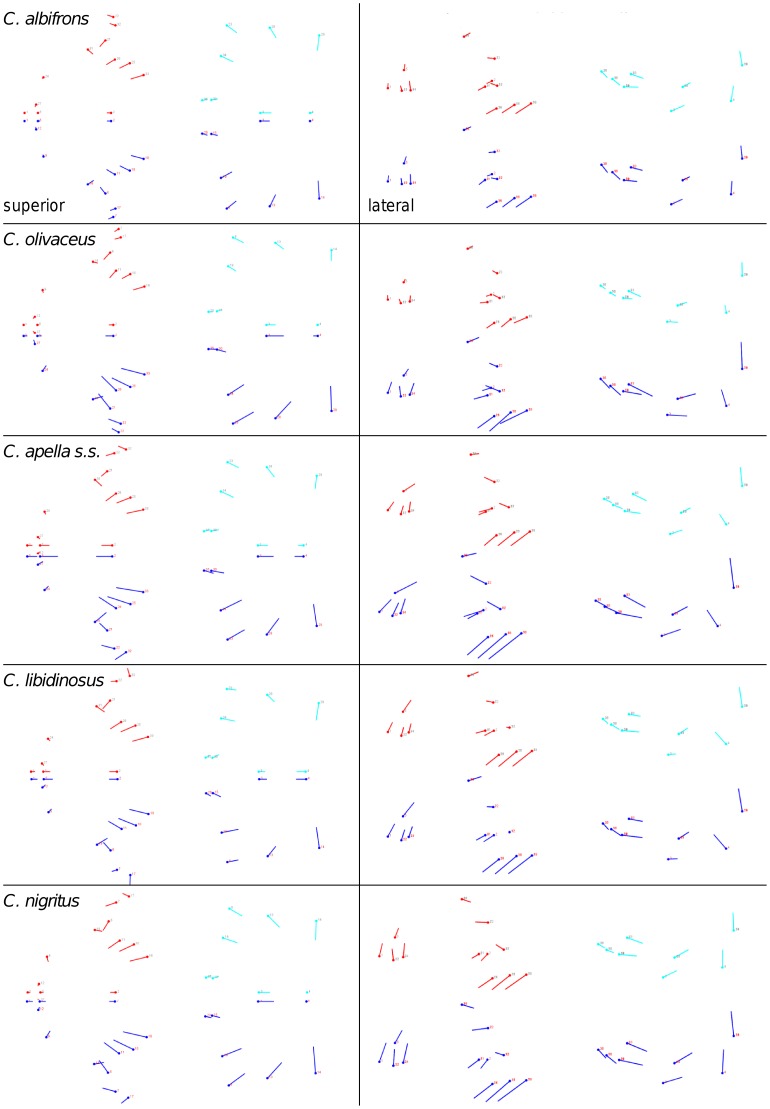
Shape change contained in the allometric face-cranial base PLS1 and the allometric PC1. Legend: oral-zygomatic PLS1 blocks colored in red and turquoise; PC1: landmark configuration colored in dark blue.

Shape change along the allometric PLS1 is similar in all species and differentiates a male cluster from a female cluster (Figure 29). The male extreme combines an anterior, inferior and medial migration of the molar teeth with anterior displacement of all zygomatic landmarks except for zygomaxillare superior which migrates posteriorly, a superior displacement of the anterior dentition and the subnasal segment, and a posterior, inferior and medial displacement of the landmarks from the middle cranial fossae and the midline basicranium, and a supero-medial displacement of the landmarks on the posterior cranial fossa. The final result is a male pattern combining a significantly narrower molar arcade and narrower cranial base, slightly more anteriorly located zygomatic roots, lower position of the molars resulting in a taller lower face, with slightly more posteriorly located infraorbital margins relative to the incisor teeth, posteriorly shorter jaws and a greater distance between the third molars and the landmarks found posteriorly to the sphenobasions, smaller petrous pyramids, sphenotemporal suture in a lower and more posterior position and posterior aspects of the pyramids and posterior end of foramen magnum in a higher position. The latter indicates that the posterior part of the cranial base faces more posteriorly rather than more inferiorly in larger crania.

Differences in mean shape configurations among the species include more posteriorly located zygomaxillare inferiors, relatively flat in their supero-inferior dimension middle cranial fossae and a relatively higher position of the anterior midline basicranium in the apelloids species and *C. olivaceus* compared to *C. albifrons*.

**Figure 30:**
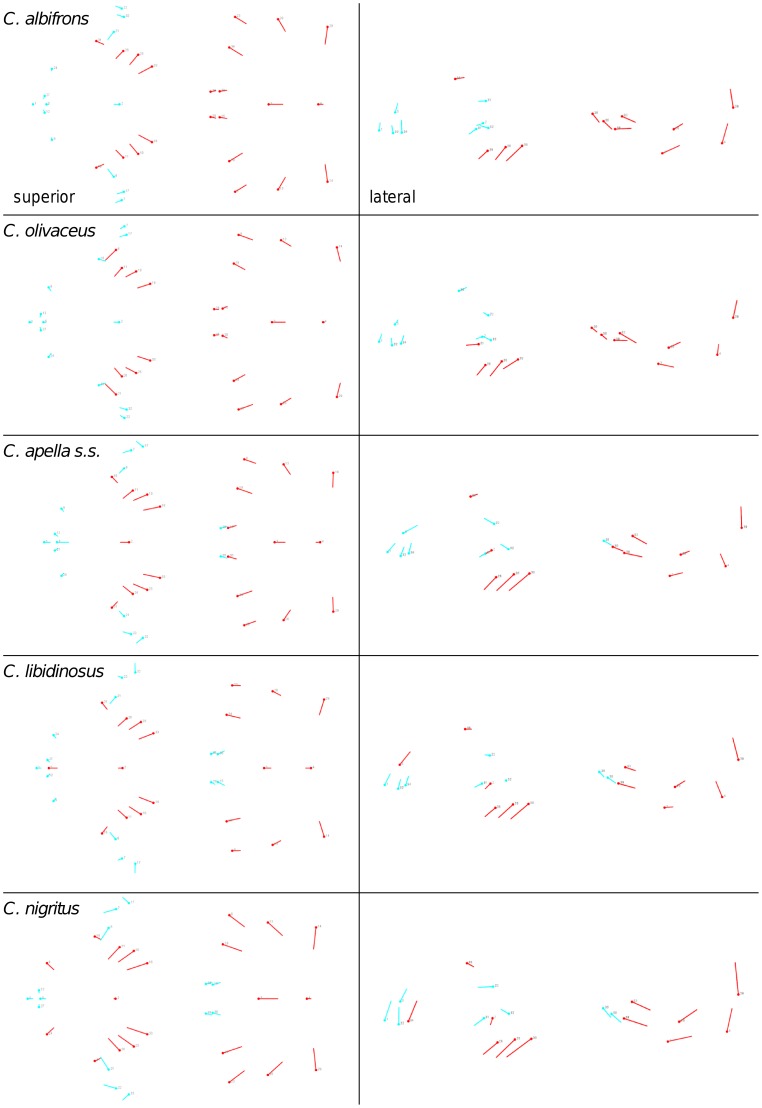
Shape change contained in the allometric maximum cranial modularity PLS1.

Shape change between the two allometric integration partitions (face-cranial base and maximum cranial modularity is very similar in all species (Figure 29, Figure 30).

#### 2. Shape change contained in the non-allometric PLS1

**Figure 31:**
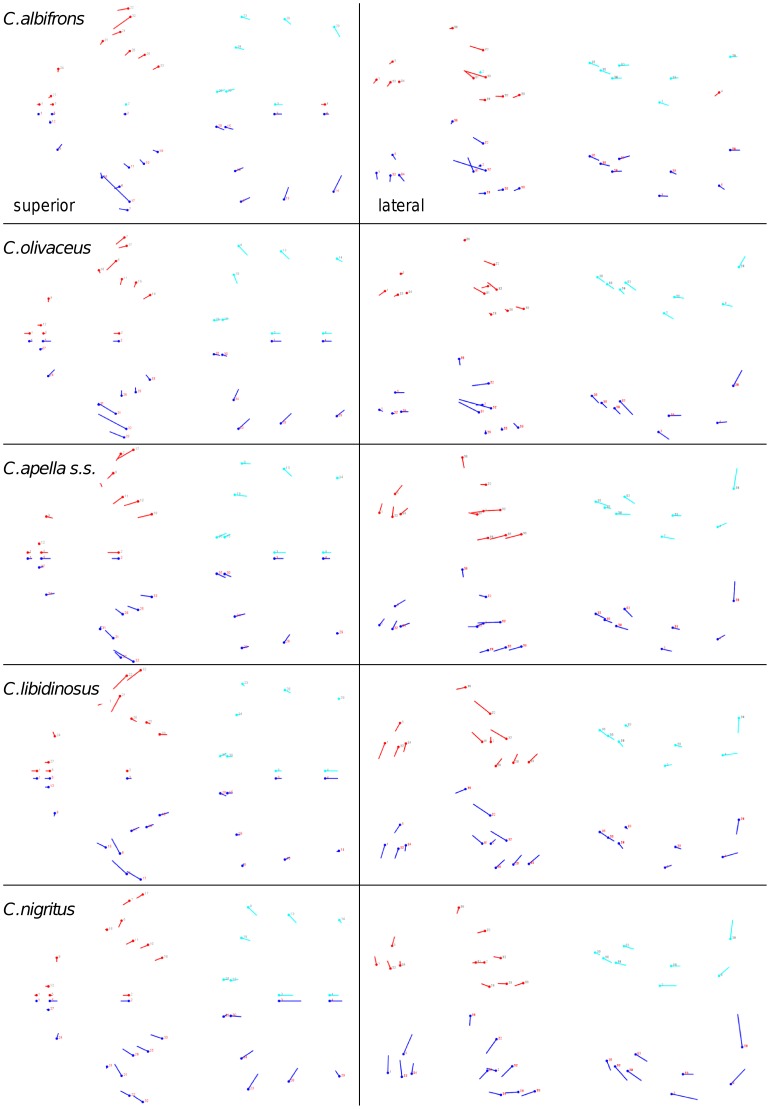
Shape change contained in the non-allometric face-cranial base PLS1 and the allometric PC1. Legend: oral-zygomatic PLS1 blocks colored in red and turquoise; PC1: landmark configuration colored in dark blue.

Similar to the situation in the face, the non-allometric face-cranial base PLS1 pattern is partly similar to its corresponding pattern in the allometric PLS1 (Figure 31). In *C. albifrons*, *C. apella s.s.*, *C. nigritus* and *C. olivaceus*, similarities include an antero-medial displacement of the molar landmarks, combined with an anterior displacement of the rostral landmarks, an antero-medial displacement of the zygomatic landmarks without zygomaxillare superior and a posterior displacement of the basicranial landmarks. In *C. libidinosus*, the displacement at the molars is postero-medial rather than antero-medial. Interspecific differences involve the supero-inferior orientation of the rostral landmarks and of the posterior petrous pyramid landmarks.

**Figure 32:**
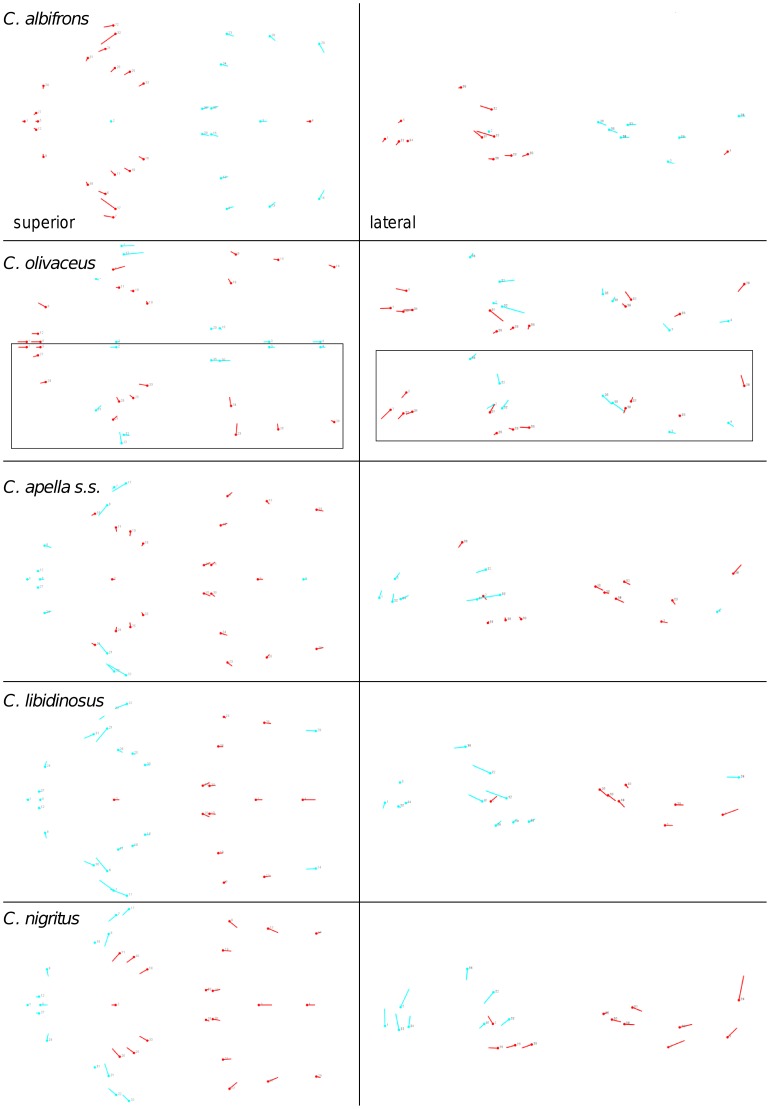
Shape change contained in the non-allometric maximum cranial modularity PLS1.

There is a correspondence in patterns between the non-allometric face-cranial base and maximum modularity PLS1 scenarios in all species but *C. apella s.s.* and *C. olivaceus* (Figure 21, Figure 32). In *C. apella s.s.*, the predominant shape change is concentrated at the inferior zygomatic root and the zygomaxillares, while the displacement at the molar landmarks is only lateral rather than postero-lateral. In *C. olivaceus*, the maximum modularity PLS1 summarizes a pattern, in which the predominant shape change is represented by an anterior displacement of the rostral landmarks and a posterior displacement of the zygomatic landmarks accompanied by a slight anterior movement of the rest of the landmarks. The *C. olivaceus* PLC2 contains shape change similar to this of the other species.

#### 3. Degree of similarity between the PLS1s and the PC1s

A nearly perfect correspondence exists between the allometric face-cranial base and the maximum cranial modularity PLS1 and the allometric PC1 in all species (Figure 29, Figure 30). Shape change is exaggerated in PC1. The same rule holds for the non-allometric face-cranial base PLS1 and the non-allometric PC1s (Figure 31, Figure 32).

#### 4. Shape pattern of the allometric and non-allometric PLS2

**Figure 33:**
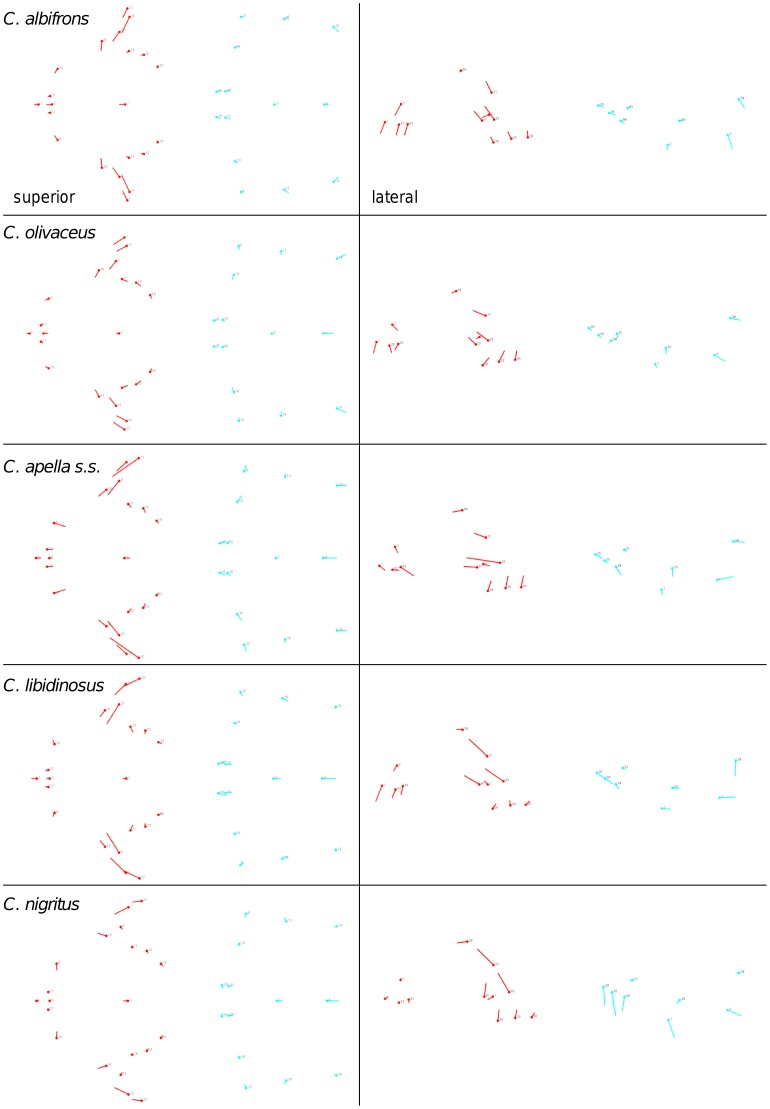
Shape change contained in the allometric face-cranial base PLS2.

In all species, the principal shape change contained in the allometric PLS2 creates an extreme morph combining an antero-medial displacement of all zygomatic landmarks (with the exception of the inferior zygomatic root in *C. nigritus*) (Figure 33). The displacement at the two zygomatic roots and zygomaxillare inferior is also superior, and it is combined with a smaller magnitude shape change concerning a superior displacement of the molars and an inferior displacement of the anterior basicranial landmarks including the sphenobasion, the pyramidal apex, the TMJs and the posterior point of the sphenotemporal suture (the latter is a high magnitude shape change in *C. nigritus*), and in some cases basion and the point at the junction between the anterior edge of the petrous pyramid and the external auditory meatus.

**Figure 34:**
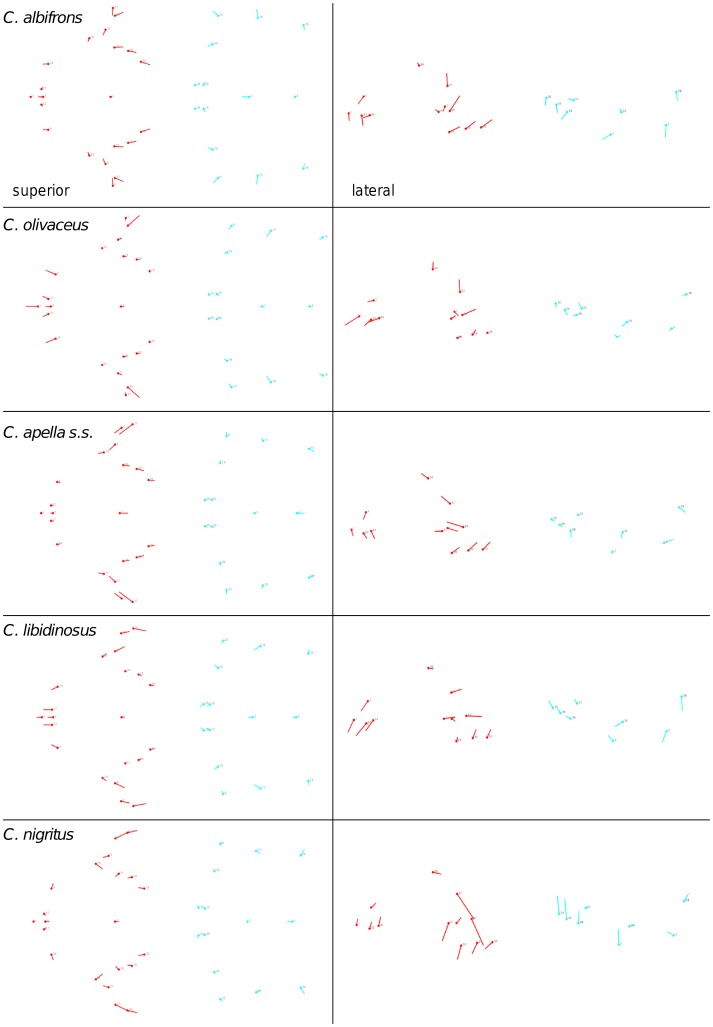
Shape change contained in the non-allometric face-cranial base PLS2.

A common interspecific pattern is not obvious in the non-allometric PLS2 (Figure 34).

### X. Inter-specific Average Facial Shape Differences

**Figure 35:**
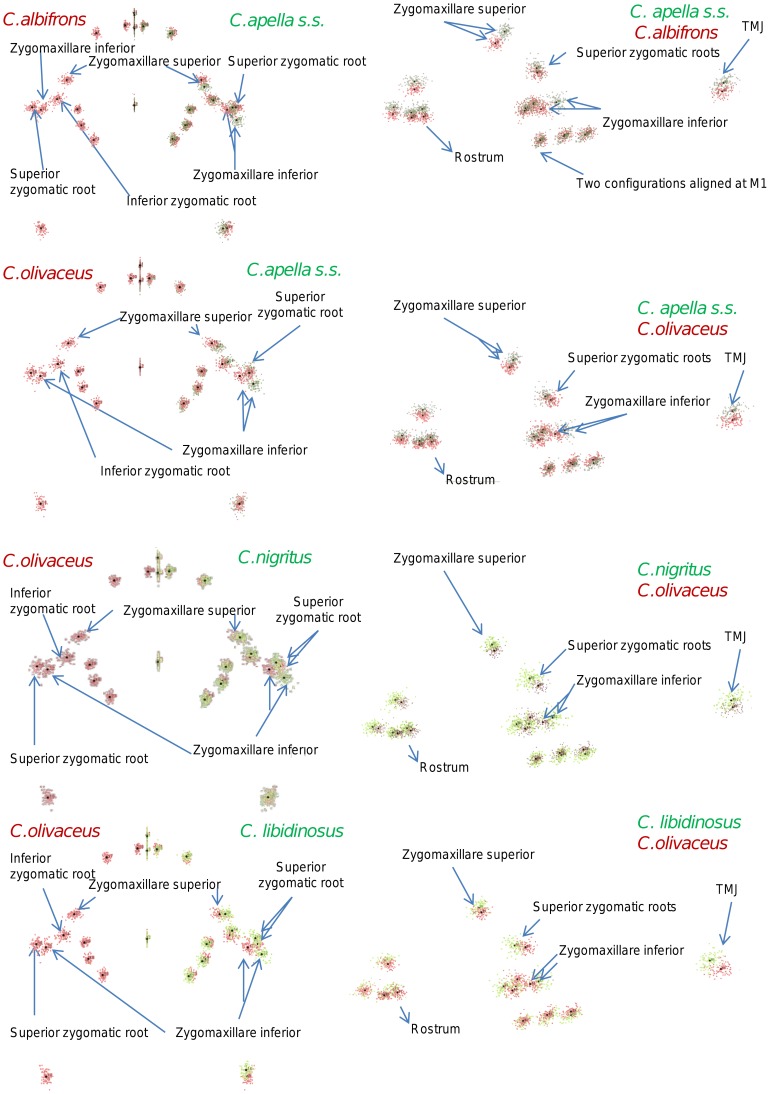
Interspecific differences in mean Procrustes configuration (containing allometry).

When comparing the average Procrustes configurations, the most conspicuous inter-specific difference between apelloid and gracile species includes the location of the zygomatico-maxillary suture (Figure 35). In *C. albifrons* and *C. olivaceus*, unlike all tufted capuchins, the zygomaxillare inferior is not significantly more posterior and lateral to the zygomatic roots, while in the apelloids the zygomatico-maxillary suture is markedly more laterally and more posteriorly positioned. Differences in mean configurations specifically distinguishing *C. apella s.s*. from *C. albifrons* include, ordered by decreasing magnitude, a zygomaxillare superior displaced in the posterior, lateral and superior direction, more superiorly shifted rostrum and TMJs, more medially located molars and TMJs, and very slightly lower position of the second and third molars combined with a slightly higher position of the superior zygomatic root in *C. apella s.s.*, resulting in a slightly taller lower face. The *C. apella s.s.* mean configuration possesses a rostrum that is placed further away from the bony orbits compared to *C. albifrons*. In addition, despite the fact that the TMJs of *C. apella s.s.* are in a more superior position relative to the molars than the TMJs of *C. albifrons,* this likely being a structural consequence of the need to create a taller lower face, the rostrum of *C. apella s.s.* is in a proportionately more superior position, restoring gape size at the incisors and the canines (Figure 35).

**Figure 36:**
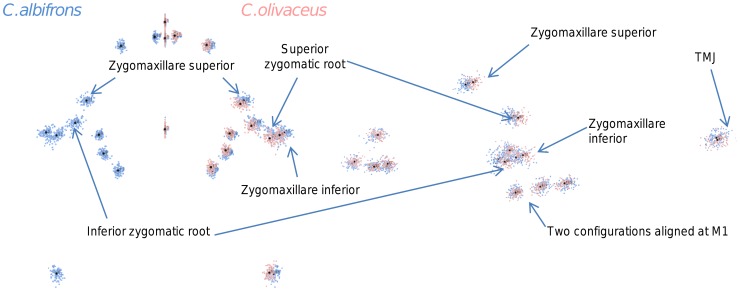
Differences in mean Procrustes configuration (containing allometry) between *C. albifrons* and *C. olivaceus*.

In comparison with *C. apella s.s.*, *C. olivaceus* and *C. albifrons* share several morphological characteristics (Figure 36). Thus, relative to *C. apella s.s.*, *C. olivaceus* displays a more anteriorly located zygomaxillare inferior but to a lesser extent than in *C. albifrons*, a more inferior, medial and anterior zygomaxillare superior, but to a lesser extent than in *C. albifrons*, more inferior TMJs, more inferiorly located rostral landmarks, but not as inferior as in *C. albifrons*, and a slightly shorter lower face than both *C. apella s.s.* and *C. albifrons*. *C. olivaceus* further differs from *C. apella s.s.* in characteristics in which *C. albifrons* does not. These include more posteriorly and inferiorly located zygomatic roots and a more medially located inferior zygomatic root.

Relative to *C. olivaceus*, *C. nigritus* resembles *C. apella s.s.* in that it possesses a more posterior and lateral zygomaxillare inferior, a more superior zygomaxillare superior that is also more anterior rather than more posterior (which is the case in *C. apella s.s.*), markedly more anterior and superior zygomatic roots (more so than *C. apella s.s.*), inferiorly shifted second and third molars, and a more laterally displaced inferior zygomatic root (but less so than in *C. apella s.s.*). Notably, *C. nigritus* possesses a rostrum that is more anteriorly projecting than these of all examined species, and an inferior zygomatic root in a more anterior position relative to *C. olivaceus* and *C. apella s.s.*

Similar to *C. nigritus* and unlike *C. olivaceus*, *C. libidinosus* possesses antero-superiorly displaced zygomatic roots and zygomaxillare superior. Again, the zygomaxillare inferior is in a more posterior position, the TMJs are more superior, and the molars are slightly more inferiorly located. Interestingly, this is the only species in which the TMJs are displaced anteriorly in comparison to *C. olivaceus*.

## Discussion

A fundamental challenge of evolutionary morphology is to identify the causes for the appearance of complexes of significantly correlated phenotypic characters. The comparative assessment of patterns and magnitudes of morphological integration and their potential association with environmental variables are essential for understanding the evolution of complex phenotypes. Research in the field of morphological integration has predominantly focused on the assessment of correlation structure and integration intensity across species and within ontogenetic trajectories and their compatibility with proposed hypotheses of modular organization. The testing of hypotheses relevant to the constraints that might be driving the structuring of morphological integration is limited. This study aimed to directly evaluate the adaptive significance of morphological integration by testing for the existence of a causal link between feeding biomechanics and patterned variation in the masticatory apparatus, with a focus on the effect of mechanical constraints imposed by diet.

### I. The Role of Diet in Structuring Integration

Our findings are consistent with reports indicating that the capuchin species known to habitually consume mechanically resistant foods tend to possess biomechanically advantageous facial characteristics compared to their untufted, gracile counterparts (e.g., longer lower face and midface, more anterior zygomatic roots). We further report that although some aspects of the face of *C. olivaceus* are reminiscent of the apelloids (which is expected given their closer phyletic relationship) and might be more biomechanically advantageous relative to *C. albifrons* (narrower dental arcade, slightly more anterior molars, more medially-located TMJs and postero-superior position of zygomaxillare superior), *C. olivaceus* is characterized by a shorter lower face and by more postero-medially positioned zygomatic roots. Consistent with the hypothesis predicting that the material properties of the food items consumed have a major impact on integration magnitudes, *C. albifrons* has a less integrated face than the members of the apelloid radiation, and even *C. olivaceus*. Furthermore, dietary adaptation appears to structure the relationship between the facial variation and cranial variation. Measures of overall multivariate correlation (RV coefficients) characterizing covariation of landmarks obtained through an allometric and a non-allometric oral-zygomatic PLS, an allometric and the non-allometric face-cranial base PLS, the allometric facial maximum modularity PLS and the allometric cranial maximum modularity PLS, strongly suggest that the apelloid species possess more integrated faces and crania compared to the soft-fruit eating and gracile *C. albifrons* (under all enumerated scenarios), or compared to both gracile species (under all enumerated scenarios excluding the non-allometric face-cranial base PLS and the non-allometric cranial modularity PLS, in which *C. olivaceus* has the highest RVs, followed by the apelloids, followed by *C. albifrons*), which lends support to the first alternative of the *FMP* hypothesis. The numeric differences in integration magnitude as measured by the RV coefficient under an allometric analysis are usually between 0.10 and 0.20 (graciles-apelloids), while differences between *C. albifrons* and the apelloids under a non-allometric scenario are usually less than 0.10.

Between-block PLS1 correlation coefficients also indicate that *C. albifrons* (allometric oral-zygomatic and maximum modularity PLS1, and non-allometric oral-zygomatic PLS1), or the two gracile species (non-allometric maximum modularity PLS1 and allometric face-cranial base and maximum cranial modularity PLS1) possess less coordinated facial and cranial shape change. *C. albifrons* possesses statistically non-significant between-block correlations in the allometric facial maximum modularity PLS1 and the non-allometric oral-zygomatic PLS1. The numeric differences in integration magnitude as measured by the PLS1 between-block correlations when they are significant for *C. albifrons* are usually rather small (between 0.05 and 0.10).

In addition, the non-allometric oral-zygomatic PLS2 and the allometric face-cranial base PLS2 distinguish *C. albifrons* and *C. olivaceus*, both characterized by non-significant axes, from all apelloids in the former analysis and from *C. apella s.s.* and *C. libidinosus* in the latter analysis, characterized by significant and in most cases high between-block correlations.

Results from the two integration indices whose calculation includes the distribution in eigenvalues among orthogonal axes (ICV on inter-landmark distances and EV on landmark coordinates) also lend support to the first alternative of the *FMP* hypothesis.

An interspecific comparison of the 95% confidence intervals of the eigenvalue variances (EV indices) obtained from a sample with 1000 bootstrap replicates indicates that facial integration of *C. albifrons* is significantly lower than these of *C. apella s.s.* and *C. libidinosus* (marginally significantly lower than this of *C. libidinosus* when adjusted EVs are considered), while both *C. olivaceus* and *C. nigritus* are characterized by marginally significantly and significantly lower facial EVs, respectively, than this of *C. apella s.s.* When adjusted EVs are considered, the facial 95% CIs of *C. apella s.s.* and *C. libidinosus* still include the highest values. Furthermore, cranial integration as measured by EVs is significantly lower in *C. albifrons* compared to the apelloids. Although the *FMP* trend as assessed by the EVs appears to apply to the cranium as a whole, it does not hold for the molar, rostral, zygomatic, oral or the basicranial blocks.

Interspecific differences in ICV integration indices calculated as the standard deviation in eigenvalues divided by the mean eigenvalue and compared between species at a common level of average trait CV indicate that the face of *C. albifrons* is significantly less integrated than the faces of the apelloids (and marginally significantly less integrated than the face of *C. nigritus* when the ICVs are adjusted) and that the cranium of *C. albifrons* is significantly less integrated than the crania of *C. olivaceus*, *C. apella s.s.* and *C. libidinosus* and marginally significantly less integrated than the cranium of *C. nigritus*. Furthermore, when sample variance is kept into account, *C. albifrons*’ and *C. olivaceus’* rostrums are significantly less integrated than the *C. apella s.s.’s* rostrum, and marginally significantly less integrated than the rostrum of *C. libidinosus* (significantly less integrated than the rostrum of *C. libidinosus* when the ICVs are adjusted). There is a non-significant trend according to which the bootstrap sample 95% CIs for the oral ICVs of both *C. albifrons* and *C. olivaceus* are lower than these of *C. apella s.s.* and *C. libidinosus* (the difference between the oral ICV range of *C. albifrons* and *C. apella s.s.* is significant). The basicranial ICVs are not distinguished on the basis of dietary groups and indicate that the likely diet-driven variation in integration magnitudes in the capuchins is not valid for the basicranium.

This is the first study to identify a potential explanatory variable (food material properties) exerting a selection pressure on facial and cranial integration magnitudes. These results are further intriguing because in most of the cases *C. apella s.s.*, the capuchin that is best known to apply considerable bite forces with their anterior dentition during palm nut cracking and tearing of the nuts’ tough fibrous husks has the highest facial integration magnitude of all apelloid species, that is significantly higher in magnitude than these of *C. albifrons* or of both gracile species (EV, ICV, respectively). *C. libidinosus*, a species who uses its anterior dentition to open hard-shelled nuts and tear their tough outer coverings, but employs stones as tools to initiate cracks in the shells, thereby alleviating the selective pressures on facial form, is characterized by a lower facial integration magnitude (ICV, EV), although not significantly lower than this of *C. apella s.s.* Finally, *C. nigritus*, a species known to extensively rely on leaves during selectively important episodes (e.g., [117], [118]) and which presumably does not primarily rely on opening hard nuts with its anterior dentition, possesses a facial integration magnitude that, in some cases is marginally significantly lower than this of *C. apella s.s.* (unadjusted EV). It is curious to note that the only two capuchin species whose geographic distributions are fully overlapping (i.e., the species are truly sympatric), *C. apella s.s.* and *C albifrons* [64], [118], are the most distinct in their facial integration magnitudes.

### II. Similarity of Integration Patterns

Results from the PLS and PCA analyses obtained in this study are consistent with previous analyses indicating a widely conserved covariation architecture at least among mammals (e.g., [31], [45]). This study reports the existence of inter-specifically shared allometric and non-allometric shape changes, or conserved integration patterns, in all examined species.

Furthermore, curiously and importantly, this study documents a general redundancy of covariation patterns between the allometric and the non-allometric PLS1, in the face and in the cranium. This study also indicates that in one of the gracile capuchin species, *C. olivaceus*, allometry plays a substantially smaller role in facial shape change compared to the other examined capuchins. Furthermore, *C. olivaceus* possesses integration magnitudes and some morphological characteristics similar to these of most tufted capuchins. Consistent with the comparison of the biomechanical parameters of *C. apella* and *C. olivaceus* with these of other platyrhines [42], this likely indicates that the common ancestor of the *C. olivaceus*-apelloid clade was already characterized by structurally buttressed crania and jaws capable of generating higher bite forces.

### III. Allometry as an Adaptive Property of Organisms

After assessing the suitability of several factors to describe the dynamics of phenotypic integration in the limb and skull in a single population of laboratory rats, Zelditch [25] reported that “growth was the principal developmental explanation of observed phenotypic covariation” (p.28). Given this, allometry cannot be simply “controlled for” in integration studies as if it represents a confounding variable, as it explains most of the covariance contained in the data, and is largely responsible for the generation of the facial phenotype, which is presented to selection. Moreover, it is often inappropriate to ignore this covariation because species may differ in the strength of allometric covariation, and arguably, some of these differences may be adaptive. Here we report that allometric shape change creates a larger size distinction between the sexes and explains a higher proportion of overall between-block squared covariation in the apelloids. Thus, allometry plays a more canalizing role on facial covariation in the apelloid capuchins. This study also reports regional variation, from one facial block to another, in the amount of the variation which may be accurately predicted by facial size.

In particular, the analysis of maximum modularity identifies a molar-TMJ block in the face and a molar-basicranial block in the cranium, which are markedly better predicted by size than the rostral-zygomatic blocks by approximately 30% in all capuchin species except for *C. olivaceus*. The allometric TMJ-molar shape change, involving a postero-lateral displacement of the molars combined with an antero-lateral displacement of the TMJs, is among the most pronounced shape changes in the facial PLS1 under both the oral-zygomatic and the maximum facial modularity hypothesis analyses. Thus, allometry governs the inter-relationship between variables of biomechanical importance and thus its interspecific variation may reflect adaptation. That the amount of allometry as a factor organizing coordinated shape change varies from species to species is suggested by the situation in *C. olivaceus*, whose integration in maximum facial modularity blocks depend very little on size-related shape change (variation in the PLS1 blocks is non-allometric, while very little allometric change in contained in PLS2). Interestingly, allometry does not play a role in integration within identified facial modules in *C. olivaceus*, but the species is not distinguished from the other capuchins on the basis of size-related shape change contained in the cranial modules. It is further found that facial and likely cranial sexual dimorphism is much less pronounced in the gracile species compared to the apelloids. In the former species, the PLS1 loadings of males and females overlap extensively or completely for the females. Centroid-size ranges are reported to be rather similar between species, yet, the overlap in facial size between the apelloid males and females is notably smaller than in the gracile species.

### IV. No Effect of Sexual Dimorphism on Integration Magnitude

The performed analyses do not yield support to any of the *HET* alternative hypotheses, indicating that the prolonged growth of male individuals does not favor the action of potential epigenetic stimuli with integrating consequences.

### V. No Evidence for Facial Modularity

Within the face, the maximum modularity scenarios on coordinates preserving allometry and on residuals from regression on centroid size do not support the existence of distinct oral and zygomatic modules. Under an allometric scenario, the three molar landmarks are always associated with the TMJs. The molar-TMJ block variably includes some zygomatic landmarks (all in *C. olivaceus*), and, in the apelloids but not in *C. albifrons* and *C. olivaceus*, some rostral landmarks. This finding underscores both a common trend shared among all capuchins and inter-specific variation possibly reflecting dietary niche differences. Under a non-allometric scenario, all capuchins contain a block including coordinated shape change between the three molar landmarks and, in all species except for *C. olivaceus*, zygomaxillare superior (ZS). This molar-ZS block is quite independent from variation in the rostral and the zygomatic landmarks (including TMJ). This finding along with comparable results reported in Goswami [45] indicates that the oral block is not a module and contains an anterior oral module and a posterior oral-nasal module.

A comparison of RV coefficients derived from facial and cranial maximum modularity PLS analyses carried out on residuals from regression on centroid size, indicates that between-block correlation is slightly stronger between the facial modules rather than between the cranial modules in all species except for *C. olivaceus*. Integration indices carried out on residuals from regression on centroid size and based on the 1) variance in eigenvalues obtained from a correlation matrix, which are thus standardized by total shape variance in the sample as well as on 2) clusters of individual landmark coordinates grouped using Ward’s method, however, do not support the existence of facial modules, or of a facial module within the cranium.

A within-species comparison of eigenvalue variances from block to block allowed assessing whether or not the cranium exhibits a modular architecture. The cranium as a structure is significantly more integrated than the face in all species but *C. apella s.s.,* which is characterized by an exceptionally high facial integration (the difference between cranial and facial EVs is not significant whether or not the EVs are adjusted; this does not support the existence of cranial modularity either). Intriguingly, integration magnitudes of the molar, zygomatic and facial blocks do not differ significantly in the gracile species and in *C. nigritus* (although the distinction between the facial and the zygomatic EVs in *C. nigritus* is marginally significant when unadjusted EVs are considered), while the face is significantly more integrated than the molar and the zygomatic blocks in *C. apella s.s.* and in *C. libidinosus* (when the EVs are adjusted, the molar and the zygomatic EVs of *C.libidinosus* are markedly lower but not significantly so than its facial EV).

When the EVs are not adjusted, the zygomatic and the molar blocks are significantly more integrated than the oral block in the gracile species, marginally significantly more integrated than the oral block in *C. apella s.s*. and *C. libidinosus* (the difference between the oral and the molar block in *C. apella s.s.* is significant) and characterized by 95% CIs higher than the 95% CI associated with the oral block in *C. nigritus*, consistent with our maximum modularity results and findings by other researchers suggesting that the oral block is composed of more than one module. When adjusted EVs are considered, the molar and the zygomatic blocks are significantly more integrated than the oral block in *C. albifrons*, *C. olivaceus* and *C. apella s.s.* and characterized by markedly higher 95% CIs than the oral block in *C. libidinosus* and *C. nigritus*. In *C. albifrons*, *C. apella s.s.* and *C. libidinosus*, the rostral-zygomatic block is significantly less integrated than the molar block, while the molar block EV is lower on average than the rostral-zygomatic EV in *C. nigritus* and *C. olivaceus*. The basicranium is significantly less integrated than the face in all species but *C. libidinosus*. The basicranium is significantly less integrated than the zygomatic block in the gracile species.

Traditionally, researchers have been focusing on widely conserved trends in modularity, while the sources of variation in modularity remain unexplored. One of the strong points of this study is that it explores and identifies inter-specific trends in within-module integration.

Results from the cluster analysis on cranial landmark coordinates using Ward’s method of linkage also do not support the existence of a basicranial module distinct from a facial module, or of individualized facial modules. Furthermore, results suggest that the cranium of the hard-object feeding apelloid capuchins might be less modular than the cranium of the gracile species.

The lack of an individualized facial module is consistent with results reported by Goswami [45], who identified the presence of six cranial modules at the order-level of most placental mammals, which generally correspond to the oro-nasal, molar, zygomatic, orbital, cranial vault and basicranial regions, using a cluster analysis with Ward’s method of linkage. However, our study does not identify the consistent presence of an anterior oral (i.e., rostral), zygomatic or basicranial module in all capuchin species, but, as explained above, hints that *C. albifrons* and *C. olivaceus* might possess more modular crania. Porto et al. [31] investigated the modular architecture of the cranium in a large number of mammalian orders and genera using matrix correlation and Mantel’s tests to assess statistically significant similarities between taxon correlation matrices and theoretical matrices representative of various modularity hypotheses. Within each taxon, the distinctiveness of a proposed phenotypic module in relation to the rest of the traits was estimated as the ratio of the average correlation of integrated traits and the average correlation of non-integrated traits.

The authors found that compared to marsupial mammals, placentals are characterized by more variable modular pattern, with most groups presenting significant total integration and significant facial-neurocranial integration, a result in agreement with the findings reported here. Further differences between Goswami’s [45] and Porto et al.’s [31] results and our results might be due to our assessment of modularity at the specific rather than at the ordinal and generic level, and possibly, on our reliance on landmarks not located at suture intersections.

Within the cranium, the face is not an individualized module under an allometric PLS scenario either. On the other hand, the maximum modularity analysis of residuals from regression on centroid size yields modules that do not unambiguously indicate the existence of a facial and a basicranial block in the cranium in all species, although this hypothesis appears to be well-supported in *C. albifrons* and *C. libidinosus* (however in the latter species graphical results indicate a notable coordinated shape change involving the zygomatics and the posterior ends of the petrous pyramids). In the other capuchins, the molar landmarks form a module with the lateral basicranial landmarks, or with all basicranial landmarks. At the same time it is interesting to note that the RV coefficients obtained from a non-allometric face-cranial base PLS are numerically very similar to the RV coefficients obtained from a non-allometric PLS implementing the maximum modularity partition.

These results suggest that the different embryological origins (face: neural crest, cranial base: paraxial mesoderm) and ossification modes (face: dermic, cranial base: endochondral) do not produce clearly regionally divergent growth and development patterns, and thus do not produce clearly distinct modules in the cranium. In addition, as shown, the axis containing the most allometric change accounts for a large proportion of cranial including face-cranial base covariance, and for a small percentage of the overall variance. Thus, allometry further obscures the effect of embryological and ossification factors. This further suggests that allometric trajectories, producing the bulk of phenotypic trait covariation, are plausibly subjected to high selective pressures.

### VI. Concluding Remarks on Integration and Modularity

An extensive analysis of the patterns and magnitudes of cranial integration encompassing numerous mammalian groups carried out by Porto et al. [31] suggests that while trait inter-relationship patterns are characterized by a relative constancy, covariance magnitudes differ markedly across groups. The distribution of the magnitudes of trait inter-relationships between modules of the entire cranium between and within mammalian orders and infraclasses led Porto et al. [31] to conclude that the evolutionary history of the mammalian skull can be viewed as one of inter-module parcellation with more clearly marked modules in lineages characterized by lower overall magnitude of integration, such as primates. The major evolutionary trend discerned by Porto et al. [31] can be explained by a decrease of the constraints on evolution via the promotion of a more modular architecture.

The constraints that might trigger the establishment of new connections among developmentally-distinct regions when they are co-opted for a common function, and thus the pathways in which advantageous inter-trait relationships might be established in a population remain unexplored. Results from this study, and interpretation of findings by other authors in the introduction of this paper (e.g., Goswami’s [47] analysis of carnivoran crania) indicate that selective factors such as food material properties may induce the structuring of covariation among functionally-related traits in the cranium.

In species relying on foods that are selectively important but mechanically challenging to ingest and masticate, whose feeding apparatuses are subjected to strong selective pressures, an advantageous combination of features conferring mechanical advantage is conceivably expected to have a positive impact on fitness; in addition, high reaction forces incurred by hard food feeding are expected to affect a greater proportion of the face.

The results of this study suggest that functionally-linked systems of structures experiencing selective pressures for a particular morphotype might behave in essentially the same way as individual traits. A biological population subjected to stabilizing selection is characterized by a reduced number of genetic variants and, by extension, by reduced phenotypic diversity. When dealing with multiple traits, such phenomena can be visualized as a reduction in the spread of the specimens’ distribution (or the size of the residuals) along the best-fit plane in a multidimensional space.

There is no *a priori* reason to exclude the possibility that morphological integration reflects adaptation and is thus at least in part the product of evolution, implying the co-selection of heritable cranial characteristics. It is unknown if integration magnitudes are affected by genotype-by-environment interactions, a phenomenon implying a coordinated shape change elicited by external stimuli during the individual’s lifespan. Young et al.’s [57] investigation on the integration intensity, structuring, and individual-character variance or the degree of canalization in two lines of active and sedentary mice (lines selected for increased voluntary activity and control lines) suggests no significant differences in fore-to-hind limb integration (including stylopods, zeugopods and autopods) between lines, leading the authors to conclude that postnatal activity levels, and thus differing lifestyles, do not significantly affect the individual trait variation or integration of limb lengths.

Following an analysis of functional integration in the rodent mandible led Zelditch et al. [159] to report that in prairie mice, which eat grass, the molar alveolus is connected to more parts, and the incisor alveolus to fewer, than in fox squirrels, which rely on gnawing hard nuts with their incisors. The authors conclude “It is possible that all those correlations depend heavily on the magnitudes and directions of strain vectors generated by gnawing relative to chewing. … The material properties of food may thus predict both the geometry of muscle arrangements and skeletal form and patterns of integration ….” [159] (p.84). Here we report results suggesting a likely situation in which feeding biomechanics plays a key role in structuring facial integration.

In conclusion, our results indicate that constraints to feeding performance might structure the intensity of integration among functionally-linked facial elements. Hard-object-feeding capuchins appear to be characterized by more tightly structured faces than the gracile *C. albifrons*, and in some cases than both *C. albifrons* and *C. olivaceus*. At this point we are unable to ascertain if the observed species-specific differences in integration intensity result from the action of selection pressures or from genotype-by-environment interactions, although, as indicated above, such results are generally consistent with a scenario of evolutionary change.

## Supporting Information

Figure S1 **Inter-specific variation in integration indices values (ICVs) with regard to sample average trait CVs.**
(TIF)

Figure S2 **Inter-specific variation in basicranial integration indices values (ICVs) with regard to sample average trait CVs.**
(TIF)

Figure S3 **Variation in integration magnitude as measured by EV between species and between modules.** Distribution of the 95% confidence intervals for EVs.
(TIF)

Figure S4 **Variation in integration intensity of maximum cranial modularity blocks measured by EVs 95% confidence intervals.**
(TIF)

Figure S5 **Variation in facial integration (ICVs) between males and females with regard to average trait CVs.** Legend: Males in green, females in red.
(TIF)

Figure S6 **Interspecific variation in facial ICVs within males and females with regard to average trait CVs.**
(TIF)

Figure S7 **Variation in integration magnitude as measured by EV between males and females.** Distribution of the 95% confidence intervals for EVs. Legend: Males in green, females in red.
(TIF)

Table S1 **Inter-specific variation in facial ICV integration indices.**
(DOCX)

Table S2 **Inter-specific variation in oral ICV integration indices.**
(DOCX)

Table S3 **Inter-specific variation in zygomatic ICV integration indices.**
(DOCX)

Table S4 **Inter-specific variation in molar ICV integration indices.**
(DOCX)

Table S5 **Inter-specific variation in rostral-zygomatic ICV integration indices.**
(DOCX)

Table S6 **Inter-specific variation in cranial ICV integration indices.**
(DOCX)

Table S7 **Inter-specific variation in basicranial ICV integration indices.**
(DOCX)

Table S8 **Variation in facial ICV integration indices between the sexes of the different species.**
(DOCX)
