# Correction: The Effect of Dietary Adaption on Cranial Morphological Integration in Capuchins (Order Primates, Genus *Cebus*)

**DOI:** 10.1371/journal.pone.0101378

**Published:** 2014-06-27

**Authors:** The PLOS ONE Staff

The word “Adaptation” is misspelled in the article title. The correct title is: The Effect of Dietary Adaptation on Cranial Morphological Integration in Capuchins (Order Primates, Genus “*Cebus*”). The correct citation is: Makedonska J, Wright BW, Strait DS (2012) The Effect of Dietary Adaptation on Cranial Morphological Integration in Capuchins (Order Primates, Genus “*Cebus*”). PLoS ONE 7(10): e40398. doi:10.1371/journal.pone.0040398
