# Supplementary figures and images for: The Effect of Dietary Adaption on Cranial Morphological Integration in Capuchins (Order Primates, Genus Cebus)
Source: PLoS One. 2012 Oct 26;7(10):e40398. doi: 10.1371/journal.pone.0040398 (PMC3482247; doi:10.1371/journal.pone.0040398)

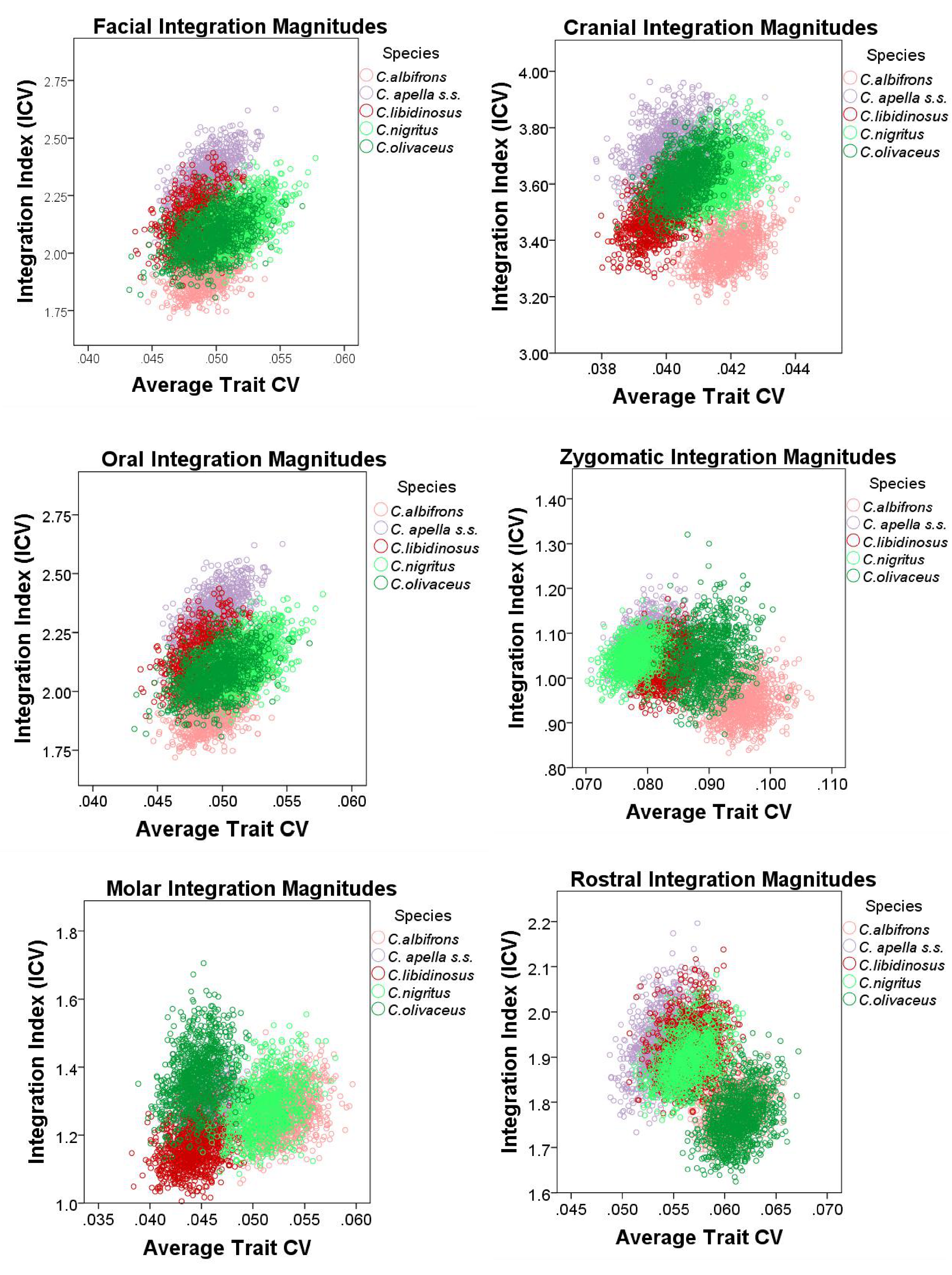

Supplement: Figure S1 — Inter-specific variation in integration indices values (ICVs) with regard to sample average trait CVs. (TIF) [file pone.0040398.s001.tif]

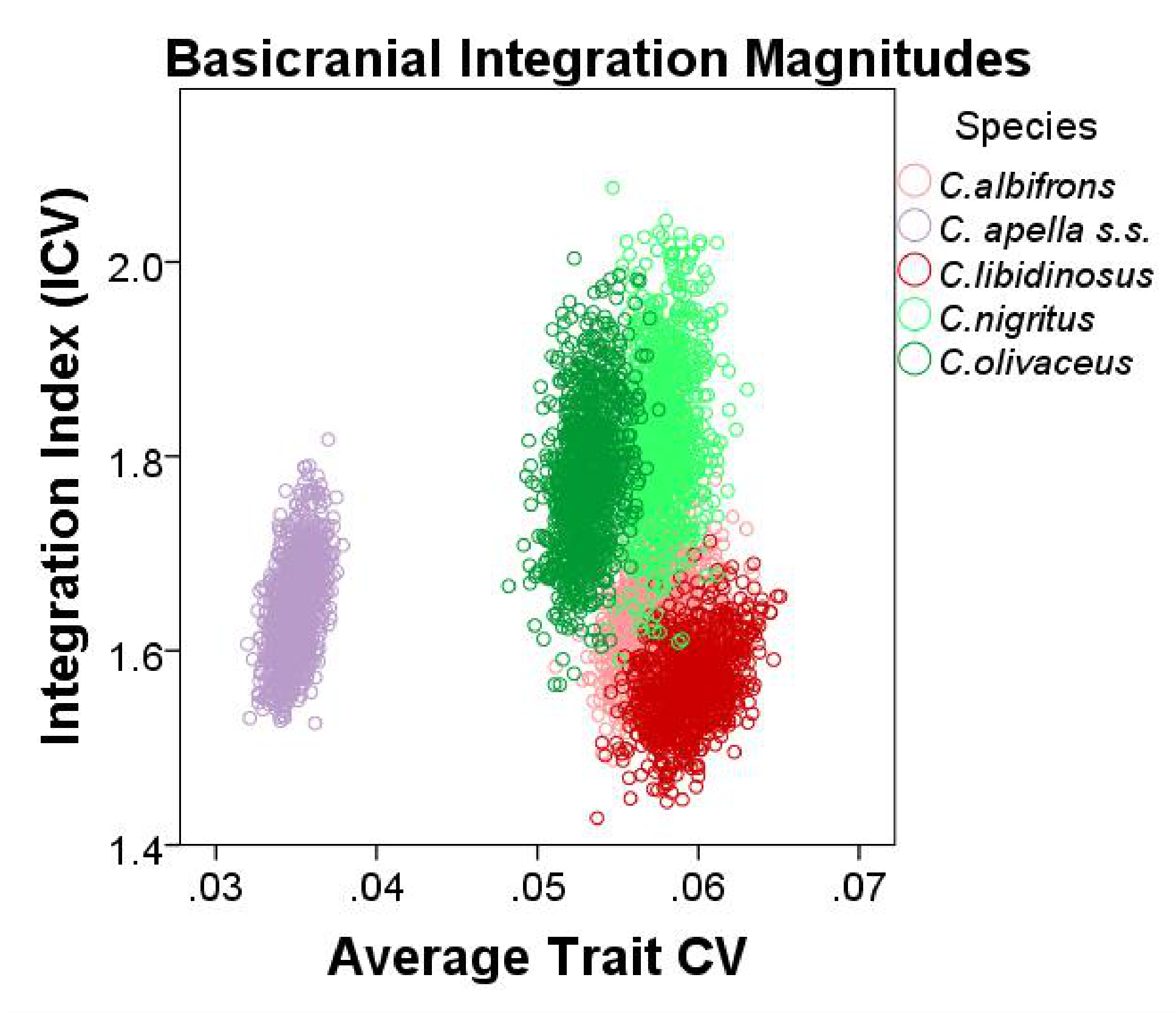

Supplement: Figure S2 — Inter-specific variation in basicranial integration indices values (ICVs) with regard to sample average trait CVs. (TIF) [file pone.0040398.s002.tif]

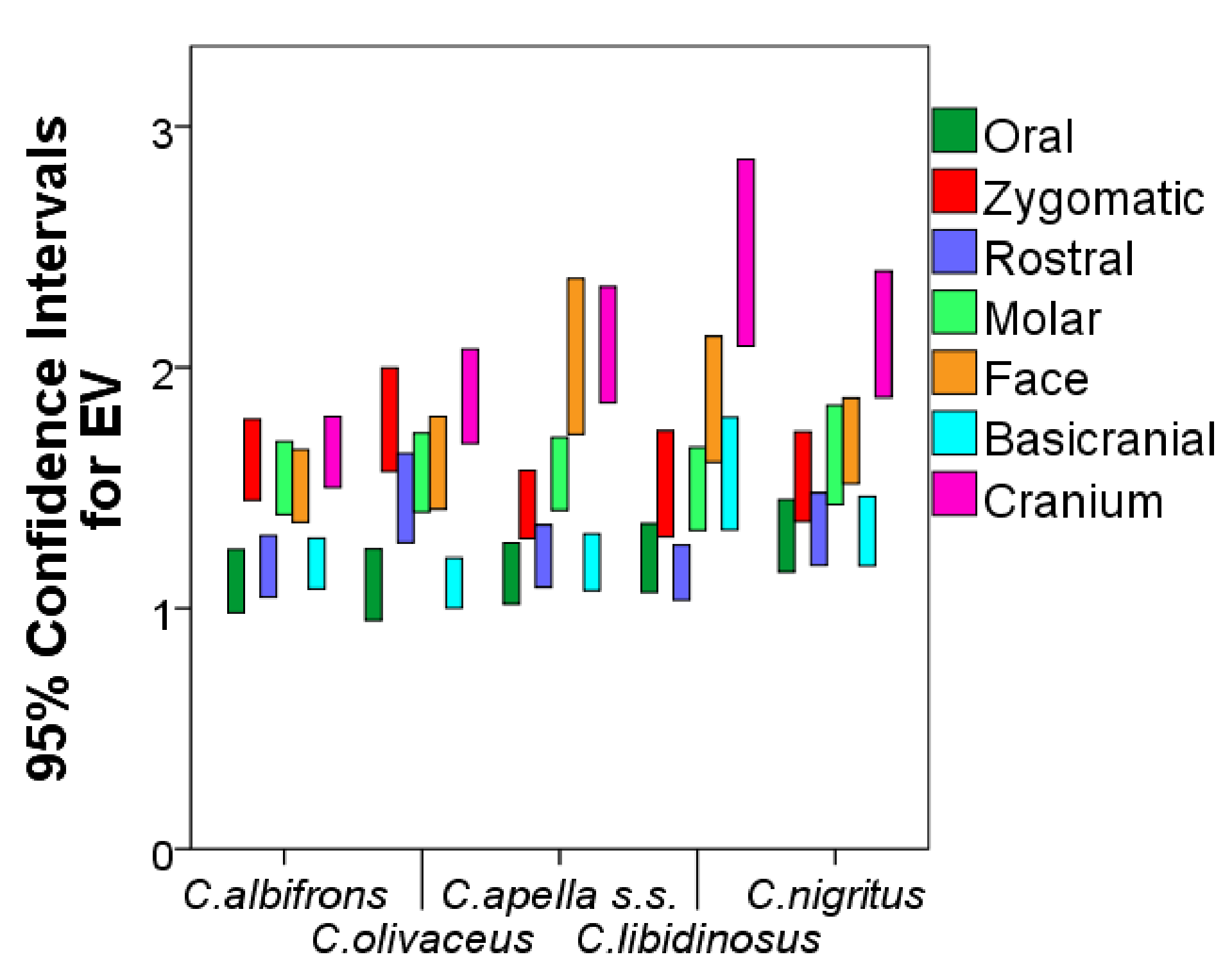

Supplement: Figure S3 — Variation in integration magnitude as measured by EV between species and between modules. Distribution of the 95% confidence intervals for EVs. (TIF) [file pone.0040398.s003.tif]

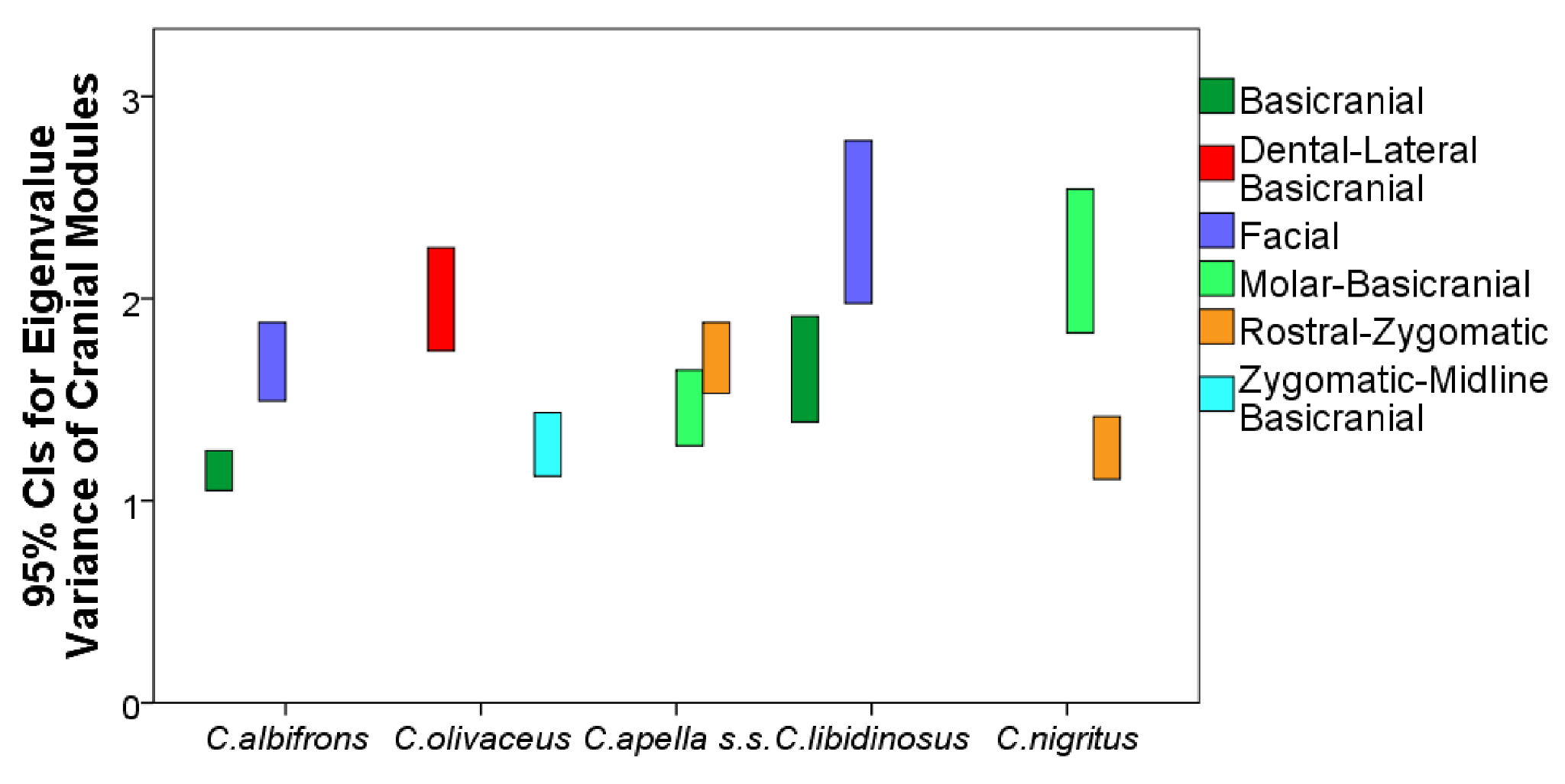

Supplement: Figure S4 — Variation in integration intensity of maximum cranial modularity blocks measured by EVs 95% confidence intervals. (TIF) [file pone.0040398.s004.tif]

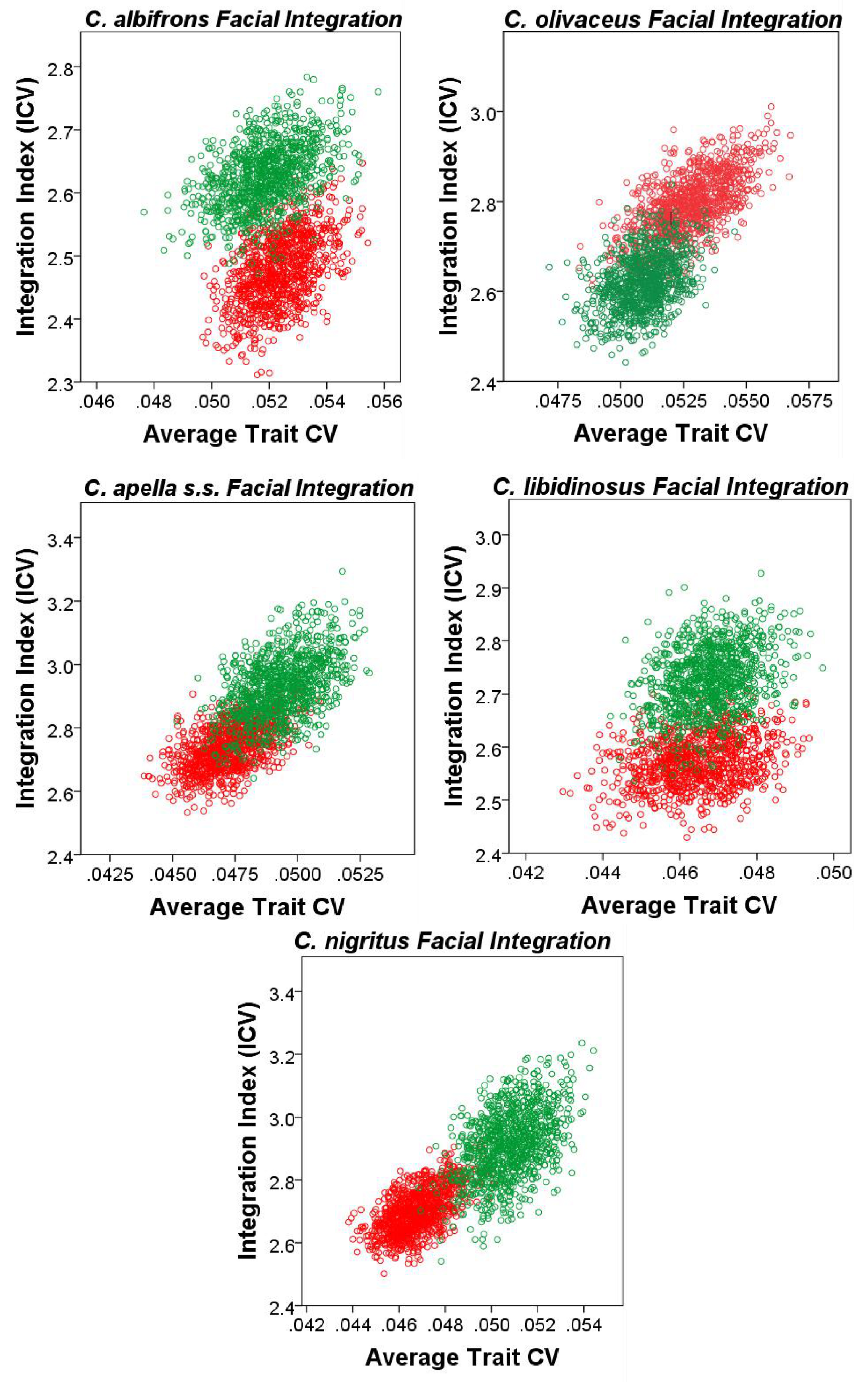

Supplement: Figure S5 — Variation in facial integration (ICVs) between males and females with regard to average trait CVs. Legend: Males in green, females in red. (TIF) [file pone.0040398.s005.tif]

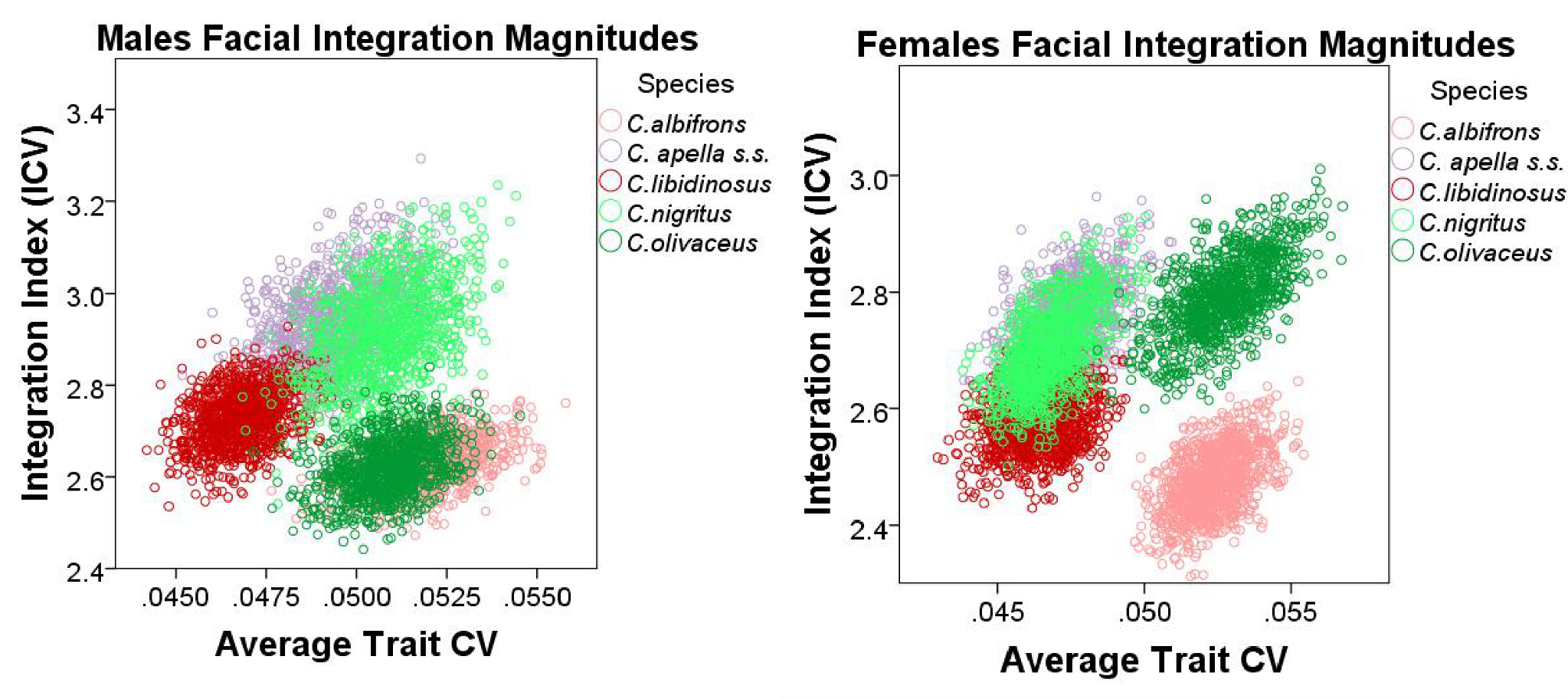

Supplement: Figure S6 — Interspecific variation in facial ICVs within males and females with regard to average trait CVs. (TIF) [file pone.0040398.s006.tif]

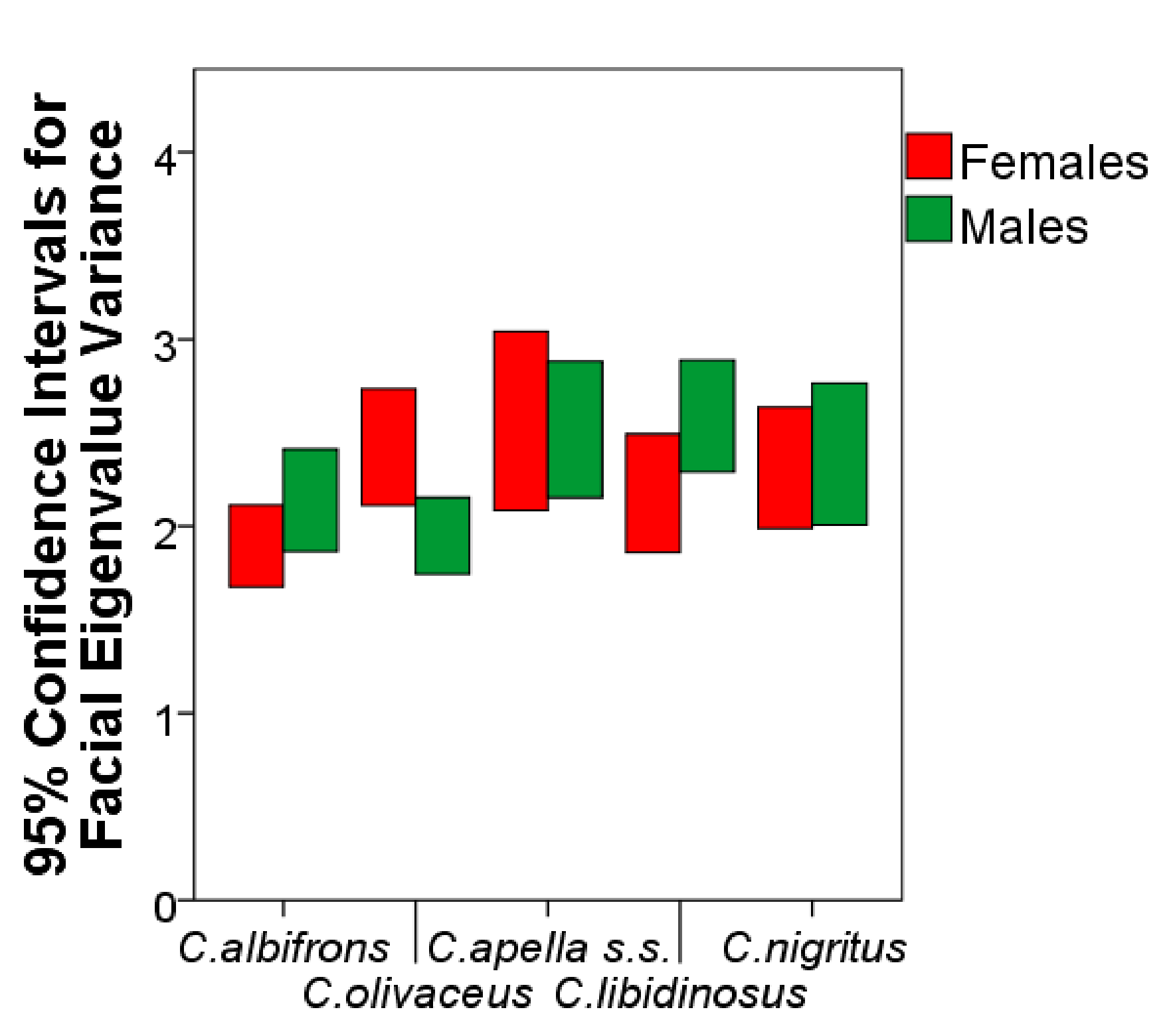

Supplement: Figure S7 — Variation in integration magnitude as measured by EV between males and females. Distribution of the 95% confidence intervals for EVs. Legend: Males in green, females in red. (TIF) [file pone.0040398.s007.tif]
