# Supplementary material for: The Effect of Dietary Adaption on Cranial Morphological Integration in Capuchins (Order Primates, Genus Cebus)
Source: PLoS One. 2012 Oct 26;7(10):e40398. doi: 10.1371/journal.pone.0040398 (PMC3482247; doi:10.1371/journal.pone.0040398)
Supplement: Table S1 — Inter-specific variation in facial ICV integration indices. (DOCX) [file pone.0040398.s008.docx]

**Table S1.** Inter-specific variation in facial ICV integration indices.

| Species | 95% CI ICV | 95% CI Mean CV | Actual ICV | Actual mean CV | ICV at a mean CV of 0.051 |
| --- | --- | --- | --- | --- | --- |
| *C. albifrons* | 2.3-2.47 | 0.051-0.0551 | 2.385 | 0.053 | 2.31-2.42 |
| *C. olivaceus* | 2.39-2.60 | 0.0506-0.0547 | 2.49 | 0.049 | 2.37-2.55 |
| *C. apella s.s.* | 2.53-2.784 | 0.0476-0.0519 | 2.653 | 0.05 | 2.56-2.81 |
| *C. libidinosus* | 2.387-2.613 | 0.047-0.051 | 2.498 | 0.049 | 2.48-2.6 |
| *C. nigritus* | 2.44-2.682 | 0.049-0.0533 | 2.56 | 0.052 | 2.41-2.69 |
