# Supplementary material for: The Effect of Dietary Adaption on Cranial Morphological Integration in Capuchins (Order Primates, Genus Cebus)
Source: PLoS One. 2012 Oct 26;7(10):e40398. doi: 10.1371/journal.pone.0040398 (PMC3482247; doi:10.1371/journal.pone.0040398)
Supplement: Table S2 — Inter-specific variation in oral ICV integration indices. (DOCX) [file pone.0040398.s009.docx]

**Table S2.** Inter-specific variation in oral ICV integration indices.

| Species | 95% CI ICV | 95% CI Mean CV | Actual ICV | Actual mean CV | ICV at a mean CV of 0.05 |
| --- | --- | --- | --- | --- | --- |
| *C. albifrons* | 1.78-2.09 | 0.0464-0.052 | 1.92 | 0.0498 | 1.77-2.12 |
| *C. olivaceus* | 1.913-2.25 | 0.0462-0.05299 | 2.07 | 0.0498 | 1.9-2.27 |
| *C. apella s.s.* | 2.135-2.51 | 0.0462-0.0522 | 2.32 | 0.0499 | 2.18-2.52 |
| *C. libidinosus* | 1.96-2.331 | 0.0457-0.0521 | 2.13 | 0.049 | 1.94-2.37 |
| *C. nigritus* | 1.963-2.312 | 0.0488-0.055 | 2.13 | 0.052 | 1.92-2.25 |
