# Supplementary material for: The Effect of Dietary Adaption on Cranial Morphological Integration in Capuchins (Order Primates, Genus Cebus)
Source: PLoS One. 2012 Oct 26;7(10):e40398. doi: 10.1371/journal.pone.0040398 (PMC3482247; doi:10.1371/journal.pone.0040398)
Supplement: Table S3 — Inter-specific variation in zygomatic ICV integration indices. (DOCX) [file pone.0040398.s010.docx]

**Table S3.** Inter-specific variation in zygomatic ICV integration indices.

| Species | 95% CI ICV | 95% CI Mean CV | Actual ICV | Actual mean CV |
| --- | --- | --- | --- | --- |
| *C. albifrons* | 0.87-1.03 | 0.0889-0.0103 | 0.952 | 0.097 |
| *C. olivaceus* | 0.935-1.17 | 0.0836-0.0969 | 1.045 | 0.09 |
| *C. apella s.s.* | 0.998-1.167 | 0.0753-0.0856 | 1.08 | 0.082 |
| *C. libidinosus* | 0.954-1.117 | 0.0779-0.0879 | 1.036 | 0.0084 |
| *C. nigritus* | 0.979-1.115 | 0.073-0.0832 | 1.048 | 0.08 |
