# Supplementary material for: The Effect of Dietary Adaption on Cranial Morphological Integration in Capuchins (Order Primates, Genus Cebus)
Source: PLoS One. 2012 Oct 26;7(10):e40398. doi: 10.1371/journal.pone.0040398 (PMC3482247; doi:10.1371/journal.pone.0040398)
Supplement: Table S4 — Inter-specific variation in molar ICV integration indices. (DOCX) [file pone.0040398.s011.docx]

**Table S4.** Inter-specific variation in molar ICV integration indices.

| Species | 95% CI ICV | 95% CI Mean CV | Actual ICV | Actual mean CV | ICV at a mean CV of 0.048 |
| --- | --- | --- | --- | --- | --- |
| *C. albifrons* | 1.146-1.40 | 0.0492-0.0571 | 1.264 | 0.054 | 1.18-1.23 |
| *C. olivaceus* | 1.209-1.552 | 0.0412-0.0484 | 1.375 | 0.0455 | 1.28-1.57 |
| *C. apella s.s.* | 1.165-1.401 | 0.043-0.0483 | 1.278 | 0.0463 | 1.18-1.40 |
| *C. libidinosus* | 1.05-1.297 | 0.0407-0.0478 | 1.167 | 0.045 | 1.12-1.38 |
| *C. nigritus* | 1.143-1.453 | 0.0471-0.0553 | 1.286 | 0.052 | 1.1-1.38 |
