# Supplementary material for: The Effect of Dietary Adaption on Cranial Morphological Integration in Capuchins (Order Primates, Genus Cebus)
Source: PLoS One. 2012 Oct 26;7(10):e40398. doi: 10.1371/journal.pone.0040398 (PMC3482247; doi:10.1371/journal.pone.0040398)
Supplement: Table S5 — Inter-specific variation in rostral-zygomatic ICV integration indices. (DOCX) [file pone.0040398.s012.docx]

**Table S5.** Inter-specific variation in rostral-zygomatic ICV integration indices.

| Species | 95% CI ICV | 95% CI Mean CV | Actual ICV | Actual mean CV | ICV at a mean CV of 0.058 |
| --- | --- | --- | --- | --- | --- |
| *C. albifrons* | 1.71-1.861 | 0.0577-0.0641 | 1.787 | 0.062 | 1.70-1.86 |
| *C. olivaceus* | 1.668-1.883 | 0.0579-0.0651 | 1.764 | 0.062 | 1.64-1.82 |
| *C. apella s.s.* | 1.8052-2.07 | 0.0502-0.057 | 1.939 | 0.0547 | 1.95-2.07 |
| *C. libidinosus* | 1.81-2.0514 | 0.53-0.060 | 1.925 | 0.058 | 1.88-2.07 |
| *C. nigritus* | 1.803-2.006 | 0.053-0.065 | 1.957 | 0.057 | 1.80-2.045 |
