# Supplementary material for: The Effect of Dietary Adaption on Cranial Morphological Integration in Capuchins (Order Primates, Genus Cebus)
Source: PLoS One. 2012 Oct 26;7(10):e40398. doi: 10.1371/journal.pone.0040398 (PMC3482247; doi:10.1371/journal.pone.0040398)
Supplement: Table S6 — Inter-specific variation in cranial ICV integration indices. (DOCX) [file pone.0040398.s013.docx]

**Table S6.** Inter-specific variation in cranial ICV integration indices.

| Species | 95% CI ICV | 95% CI Mean CV | Actual ICV | Actual mean CV | ICV at a mean CV of 0.041 |
| --- | --- | --- | --- | --- | --- |
| *C. albifrons* | 3.244-3.509 | 0.0406-0.0432 | 3.37 | 0.042 | 3.21-3.45 |
| *C. olivaceus* | 3.461-3.782 | 0.0395-0.042 | 3.63 | 0.040 | 3.49-3.82 |
| *C. apella s.s.* | 3.549-3.883 | 0.0388-0.0414 | 3.71 | 0.040 | 3.59-3.93 |
| *C. libidinosus* | 3.344-3.66 | 0.0385-0.041 | 3.5 | 0.039 | 3.46-3.68 |
| *C. nigritus* | 3.477-3.79 | 0.0404-0.04308 | 3.63 | 0.042 | 3.44-3.78 |
