# Supplementary material for: The Effect of Dietary Adaption on Cranial Morphological Integration in Capuchins (Order Primates, Genus Cebus)
Source: PLoS One. 2012 Oct 26;7(10):e40398. doi: 10.1371/journal.pone.0040398 (PMC3482247; doi:10.1371/journal.pone.0040398)
Supplement: Table S7 — Inter-specific variation in basicranial ICV integration indices. (DOCX) [file pone.0040398.s014.docx]

**Table S7.** Inter-specific variation in basicranial ICV integration indices.

| Species | 95% CI ICV | 95% CI Mean CV | Actual ICV | Actual mean CV |
| --- | --- | --- | --- | --- |
| *C. albifrons* | 1.536-1.729 | 0.0541-0.061 | 1.63 | 0.057 |
| *C. olivaceus* | 1.647-1.931 | 0.051-0.0559 | 1.77 | 0.053 |
| *C. apella s.s.* | 1.552-1.749 | 0.033-0.037 | 1.64 | 0.035 |
| *C. libidinosus* | 1.481-1.648 | 0.056-0.0632 | 1.56 | 0.059 |
| *C. nigritus* | 1.673-1.99 | 0.0547-0.0606 | 1.82 | 0.058 |
