# Supplementary material for: The Effect of Dietary Adaption on Cranial Morphological Integration in Capuchins (Order Primates, Genus Cebus)
Source: PLoS One. 2012 Oct 26;7(10):e40398. doi: 10.1371/journal.pone.0040398 (PMC3482247; doi:10.1371/journal.pone.0040398)
Supplement: Table S8 — Variation in facial ICV integration indices between the sexes of the different species. (DOCX) [file pone.0040398.s015.docx]

**Table S8.** Variation in facial ICV integration indices between the sexes of the different species.

| Species | Actual ICV | Actual mean CV | ICV at a mean CV of 0.049 |
| --- | --- | --- | --- |
| *C. albifrons ♂* | 2.63 | 0.0514 | 2.55-2.58 |
| *C. albifrons ♀* | 2.48 | 0.0517 | At mean CV of 0.05 : 2.38-2.45 |
| *C. olivaceus ♂* | 2.62 | 0.0508 | 2.48-2.67 |
| *C. olivaceus ♀* | 2.79 | 0.0508 | At mean CV of 0.05 : 2.63-2.78 |
| *C. apella s.s. ♂* | 2.928 | 0.042 | 2.72-3.12 |
| *C. apella s.s. ♀* | 2.745 | 0.0468 | 2.68-2.92 |
| *C. libidinosus ♂* | 2.731 | 0.0491 | 2.78 |
| *C. libidinosus ♀* | 2.572 | 0.046 | 2.57-2.64 |
| *C. nigritus ♂* | 2.92 | 0.0504 | 2.65-3.05 |
| *C. nigritus ♀* | 2.704 | 0.0465 | 2.72-2.87 |
